# Supplementary material for: Ribogenesis boosts controlled by HEATR1-MYC interplay promote transition into brain tumour growth
Source: EMBO Rep. 2024 Jan 15;25(1):14. doi: 10.1038/s44319-023-00017-1 (PMC10897169; doi:10.1038/s44319-023-00017-1)
Supplement: Supplementary file 1 — Appendix revised [file 44319_2023_17_MOESM1_ESM.pdf]

Appendix

Supplemental figures and legends

Appendix Figure S1 - pg 1
Appendix Figure S2 - pg 2
Appendix Figure S3 - pg 3
Appendix Figure S4 - pg 4
Appendix Figure S5 - pg 5
Appendix Figure S6 - pg 6
Appendix Figure S7 - pg 7

Supplemental tables and legends

Appendix Table S1 - pg 8
Appendix Table S2 - pg 10

Appendix Figure S1

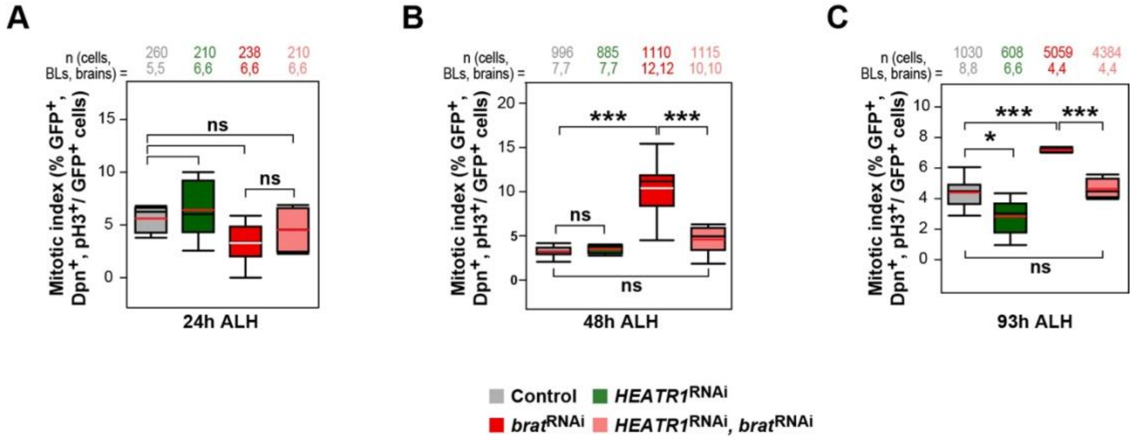

Appendix Figure S1. Mitotic cell index of *brat*-deficient tumour and control brains following *HEATR1* knockdown

A-C Mitotic cell index quantifications (% $GFP^+Dpn^+pH3^+$/ GFP^+ cells) of type II NSC lineages expressing *CD8-GFP* (control) and *HEATR1*^{RNAi}, *brat*^{RNAi} (tumour) or both *HEATR1*^{RNAi}, *brat*^{RNAi} (*HEATR1*-deficient tumour) immunostained with GFP, Dpn and pH3 at 24 h (A; biological replicates: 5-6), 48 h (B; biological replicates: 7-12) and 93 h (C; biological replicates: 4-8) ALH. Unpaired two-tailed t-tests, except in B (Mann-Whitney test, *HEATR1*^{RNAi} versus control). Representative images in Figures 2A-D, L-O and Q-T for 24 h, 48 h and 93 h ALH, respectively.

Data Information: Box plots represent 25th and 75th percentiles, central black bands indicate medians, central red or white bands specify means, whiskers indicate 10th and 90th percentiles. ***$p \leq 0.001$; *$p \leq 0.05$; $p > 0.05$, ns (non-significant).

Appendix Figure S2

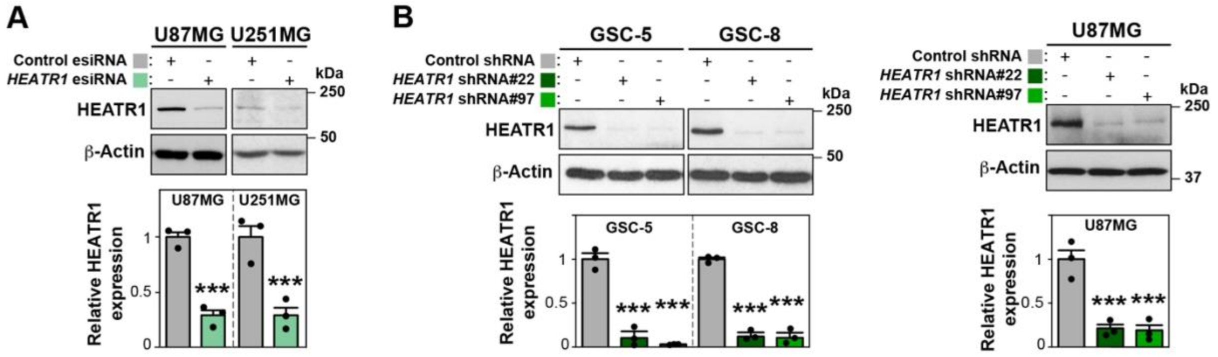

Appendix Figure S2. Efficient *HEATR1* knockdown in GBM cell lines and GSCs

A, B. Immunoblots of HEATR1 in GBM cell lines (U87MG; U251MG) 48 hpt with *HEATR1*-esiRNA or control *GFP*-esiRNA (A) and of GSCs (GSC-5; GSC-8) and U87MG cells 168 hpi with *HEATR1*-shRNAs or control shRNAs (B). β-Actin: loading control. HEATR1 signal quantifications, error bars: s.e.m, biological replicates: 3. Unpaired two-tailed t-tests (***p ≤ 0.001).

Appendix Figure S3

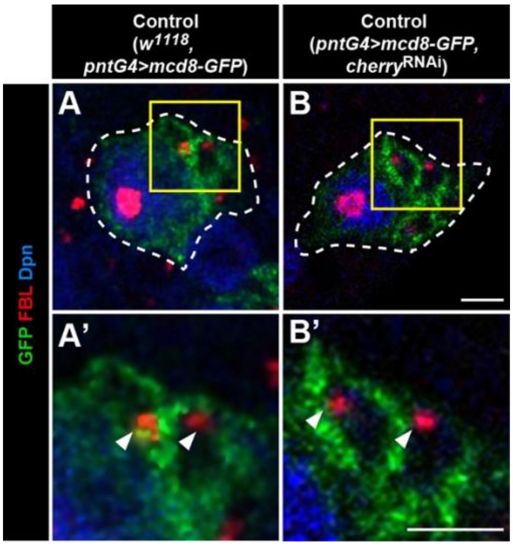

Appendix Figure S3. Representative images of control *Drosophila* RNAi expression assays

A-B' Immunostainings of GFP, FBL and Dpn in control type II NSC lineages expressing *CD8-GFP* in the GD RNAi library host isogenic background (A) or simultaneously expressing *cherry*^{RNAi} (B) at 24 h ALH. Insets: higher magnification in A', B'. Arrowheads: nucleoli. Scale bars: 5 µm. Quantification of nucleoli sizes and sample numbers in Figure 4E.

Appendix Figure S4

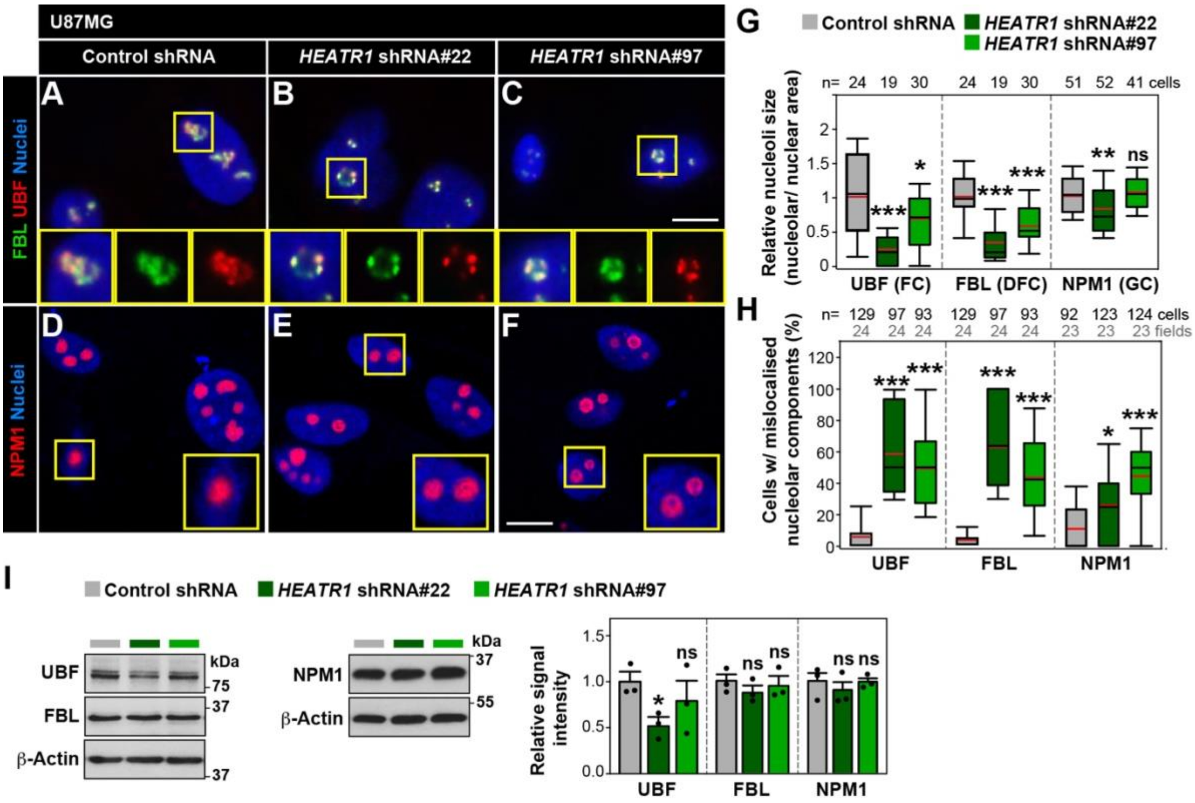

Appendix Figure S4. *HEATR1* inhibition impairs nucleoli size and architecture in GBM cells

A-H. Immunostainings of FBL, UBF (A-C) and NPM1 (D-F) in U87MG GBM cells 168 hpi with control shRNAs (A, D) or *HEATR1*-shRNAs (B-C, E-F). FBL, UBF and NPM1 mark dense fibrillar centres (DFCs), fibrillar centres (FCs) and granular components (GCs) of nucleoli functional domains, respectively. Nuclei (DAPI). Insets: higher magnification. Scale bars: 10 µm. Nucleoli size quantification relative to nucleoli domain areas (maximum areas; G): 19-30 (UBF), 19-30 (FBL) and 41-52 (NPM1) cells from 3 biological replicates. Number of cells (%) with mislocalised nucleolar components (H): 24 (UBF), 24 (FBL) and 23 (NPM1) cell images (fields) from 3 biological replicates. Unpaired two-tailed t-tests except in G (Mann-Whitney tests: UBF, FBL and NPM1 analysis upon *HEATR1*-shRNA #22 versus control) and in H (Mann-Whitney tests: UBF and FBL analysis upon *HEATR1*-shRNA #22 or #97 versus controls).

I. Immunoblots of U87MG cells 168 hpi with *HEATR1*-shRNAs or control shRNAs with indicated antibodies. β-Actin: loading control. Quantifications of relative signals. Error bars: s.e.m., biological replicates: 3, unpaired two-tailed t-tests. UBF and FBL blots share the same loading β-Actin controls as in Appendix Figure S6A since immunoblot membrane was cut and probed with the different indicated antibodies (see data source files).

Data Information: Box plots represent 25th and 75th percentiles, central black bands indicate medians, central red bands specify means, whiskers indicate 10th and 90th percentiles. ***p ≤ 0.001; **p ≤ 0.01; *p ≤ 0.05; p > 0.05, ns (non-significant).

Appendix Figure S5

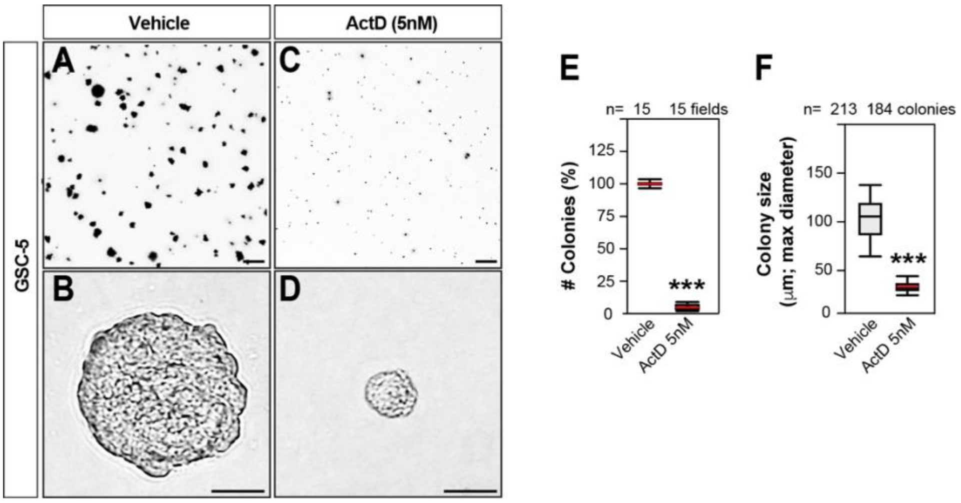

Appendix Figure S5. RNA Pol I inhibition by ActD abolishes GSC tumourigenic potential

A-F Soft agar colony formation assay of GSC-5 treated with vehicle (A, B) or ActD (C, D). Colony number (%) quantification (E): 15 fields per condition from 3 biological replicates. Colony size quantification (F): 184-213 colonies from 3 biological replicates. Mann-Whitney tests.
Scale bars: 400 µm (A, C); 50 µm (B, D).

Data Information: Box plots represent 25th and 75th percentiles, central black bands indicate medians, central red bands specify means, whiskers indicate 10th and 90th percentiles. ***p ≤ 0.001.

Appendix Figure S6

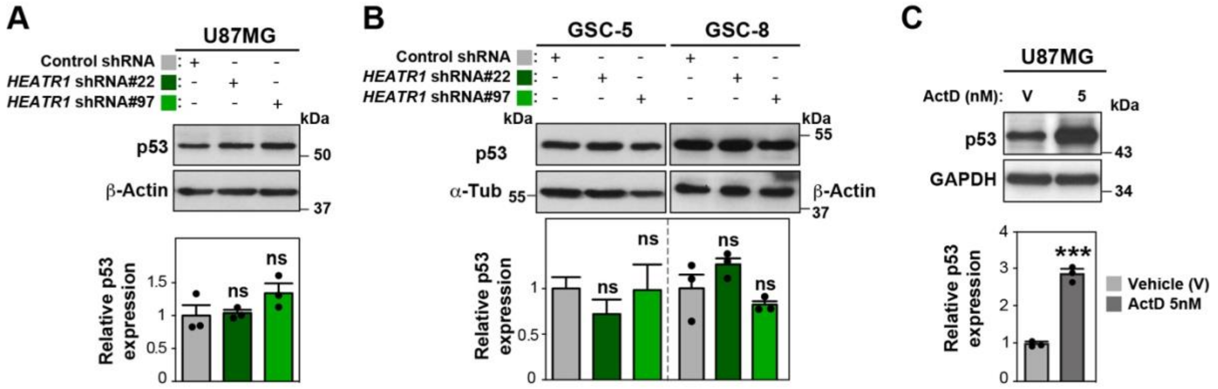

Appendix Figure S6. p53 total levels are not affected by *HEATR1* inhibition in GBM cells or GSCs

A, B Immunoblots of p53 in U87MG cells (A) and GSCs (GSC-5; GSC-8; B) 168 hpi with *HEATR1* or control shRNAs. β-Actin, α-Tubulin: loading controls. p53 signal quantifications. Biological replicates: 3 (U87MG; GSC-8), 6 (GSC-5). Error bars: s.e.m. Unpaired two-tailed t-tests except in B (Mann-Whitney test: GSC-8 upon *HEATR1* shRNA #97 versus control).

C Immunoblots of p53 in U87MG cells treated with ActD (5 nM) or vehicle for 24 h. GAPDH: loading control. p53 signal quantifications, error bars: s.e.m, biological replicates: 3, unpaired two-tailed t-test.

Data Information: ***$p \leq 0.001$; $p > 0.05$, ns (non-significant).

Appendix Figure S7

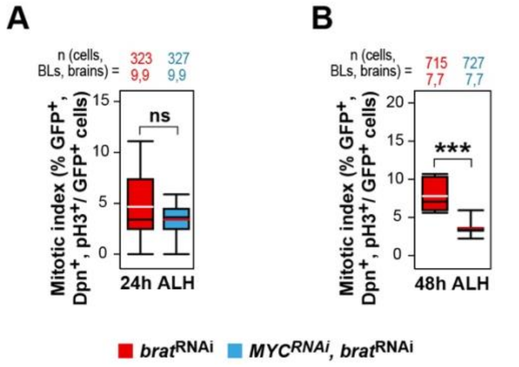

Appendix Figure S7. Mitotic cell index of *brat*-deficient tumour brains following *MYC* knockdown

A, B Mitotic cell index quantifications (% $GFP^{+}Dpn^{+}pH3^{+}$/ GFP^{+} cells) of type II NSC lineages expressing *CD8-GFP* and *brat*^{RNAi} (tumour) or both *MYC* ^{RNAi}*, brat*^{RNAi} (*MYC*-deficient tumour) immunostained with GFP, Dpn and pH3 at 24 h (A; biological replicates: 9) and 48 h (B; biological replicates: 7) ALH. Unpaired two-tailed t-tests. Representative images in Figure 6A, B and E, F for 24 h and 48 h ALH, respectively.

Data Information: Box plots represent 25th and 75th percentiles, central black bands indicate medians, central red or white bands specify means, whiskers indicate 10th and 90th percentiles. ***$p \leq 0.001$; $p > 0.05$, ns (non-significant).

Appendix Table S1

Sample	Age (years)	Gender	Clinical Diagnosis (WHO grade)	Brain region	*IDH* status	RT-qPCR	WB	IHC
CB9	33	M	Normal*	Fr	N/A	+	+	-
CB10	32	M	Normal	Fr	N/A	+	+	-
CB18	61	M	Normal*	Fr	N/A	+	-	-
CB19	70	M	Normal	Te	N/A	+	-	-
CB20	57	F	Normal	Fr	N/A	+	+	-
CB21	57	M	Normal	Fr	N/A	+	-	-
CB77	77	M	Normal	Fr	N/A	-	+	-
CB78	61	M	Normal	Ce	N/A	-	+	-
CB79	65	M	Normal	Ce	N/A	-	+	-
CB84	36	M	Normal	Te	N/A	-	-	+
CB85	52	F	Normal	Pa	N/A	-	-	+
CB86	62	M	Normal	Oc	N/A	-	-	+
CB87	72	M	Normal	Fr	N/A	-	-	+
CB88	37	M	Normal	Fr	N/A	-	-	+
CB89	34	M	Normal	Fr	N/A	-	-	+
CB90	34	M	Normal	Fr	N/A	-	-	+
CB91	70	M	Normal	Te	N/A	-	-	+
CB92	73	M	Normal	Te	N/A	-	-	+
CB93	52	F	Normal	Te	N/A	-	-	+
CB55	38	F	DA (II)	Fr	mut	+	+	+
CB56	38	M	DA (II)	Fr	mut	+	+	+
CB57	44	M	DA (II)	Iv/Pv	mut	+	+	+
CB58	53	F	DA (II)	Oc	mut	+	-	+
CB60	34	F	DA (II)	Te	mut	+	-	+
CB61	31	M	DA (II)	Fr	mut	+	+	+
CB62	63	M	DA (II)	Te	mut	+	-	+
CB63	37	F	DA (II)	N/A	mut	+	+	+
CB76	25	M	DA (II)	Fr	mut	+	-	+
CB13	40	F	GBM (IV)	Fr	wt	+	-	-
CB17	69	F	GBM (IV)	Fr	wt	+	-	-
CB22	73	F	GBM (IV)	Te	wt	-	+	-
CB23	53	M	GBM (IV)	Fr	wt	+	-	-
CB24	53	F	GBM (IV)	Fr	wt	+	-	-
CB29	68	M	GBM (IV)	Pa	wt	-	+	-
CB30	73	M	GBM (IV)	Fr	wt	+	-	-
CB31	64	M	GBM (IV)	Fr	wt	+	-	-
CB32	63	M	GBM (IV)	Fr	wt	+	-	-
CB33	58	M	GBM (IV)	Fr	wt	+	-	-
CB34	70	F	GBM (IV)	Fr	wt	+	-	-
CB35	65	F	GBM (IV)	Fr	wt	+	+	-
CB42	73	F	GBM (IV)	Fr	wt	-	+	-
CB43	69	F	GBM (IV)	Fr	wt	-	+	-
CB44	41	F	GBM (IV)	Fr	wt	-	+	-
CB45	54	M	GBM (IV)	Fr	wt	-	+	-
CB65	23	F	GBM (IV)	Fr	wt	+	+	+
CB66	62	F	GBM (IV)	Te	wt	+	-	+
CB67	70	M	GBM (IV)	Oc	wt	+	-	+
CB68	77	M	GBM (IV)	Fr	wt	+	+	+
CB69	66	M	GBM (IV)	Te	wt	+	-	+
CB70	61	M	GBM (IV)	Te	wt	+	+	+
CB72	47	M	GBM (IV)	Te	wt	+	-	+
CB73	51	F	GBM (IV)	Tpa	wt	+	+	+
CB80	84	M	GBM (IV)	Pa	wt	+	-	-
CB82	40	M	GBM (IV)	Fr	wt	-	-	+

Appendix Table S1. Human tissue samples used for gene and protein expression analysis

M: male. F: female. DA: Diffuse astrocytoma (grade II). GBM: glioblastoma (grade IV). *Normal appearing brain from adjacent to glioma. Fr: frontal. Ce: cerebellum. Te: temporal. Pa: parietal. Oc: occipital. Iv: intraventricular. Pv: periventricular. Co: cortex. Ft: frontotemporal. Tpa: temporoparietal. N/A: not available. WB: Western blotting. IHC: immunohistochemistry.

Appendix Table S2

Gene (symbol)	Forward Primer (5' → 3')	Reverse Primer (5' → 3')	Source
Drosophila			
ase	CACCTACCAACTGCTGACG	GCTGCTGCTGCTAATGTTG	This paper
CG6724	ACACGGAGACACATTCCACC	GGTGGACTGGATATCGCTGG	This paper
Ilk	TTCTCGCCGTCAGATCATCG	TGTTGCGAGAGGAATGGCTT	This paper
CG2126	GGCAGGATCCGTGAAACGTA	AGCTGTGCACTGGATGTTGT	This paper
Hsf	ATGCCGATACCAATCGCTTGA	CTTGGCGAATTGCGCTTGA	This paper
nop5	GCCTCTGGTTGGTAGGTGAA	GCGGCTCACAAGGTTAAACT	This paper
NS1	AAGCACAAGATCGAAAAGAAGGT	TGTCATCCTTGAAGGGACAGA	This paper
Prp3	AATAGCTTCTTCCTGGGCCG	AACATACGCGCCAAGAAACG	This paper
en	AGCGATTCTCGTTGAACTCC	CAGCCAAAGGACAAGACCA	This paper
SC35	CCGTCTAGCATGCGACCAT	CGGATTCGCATTTGTTCGCT	This paper
Kap-α1	AGAGGCTCTAGAATGCCGGA	GCTCTCCAGTCCCCATGATG	This paper
link	TTTCAAGCCAGCAGGAGGAG	CTGTGGTCTGGTCAGGTCAC	This paper
ATPsynγ	CCCACGCAGAACACCTTAGT	CTCATCGCAGTGACTTCGGA	This paper
GC3499	TCTTCGTCCGCTCTTCTCACA	CGCTTTTGTTCCGCCTCAA	This paper
gus	GGCAAGGGTTCGGGATCTAA	TTGCGTTTAGAGGACTGCGA	This paper
Mob3	GGGCAAGACCTTTAGGCCAAA	CGATTGAAGAAGTCCACAACGTG	This paper
Nop60B	CGCAGCATAGACTCGTCCTT	AGTCTGAGCGCGATGGTATG	This paper
mRpL12	CGAACTTGACCAGCTTGACCT	AAACTGAACCTGCCCGAGAC	This paper
Obp44a	GTTCTTGTCGGCGCACTT	GGGCTTCAAGGTGGAGAAC	This paper
Fis1	TAGTCACGCCTTCCATCTGG	TACGCATTCTGTCTGGTCCG	This paper
Kr-h2	GCTACGTGCTGCCCATCTTTA	GGCAATCTCTGGTGCAAACG	This paper
Prosβ7	ATTTGGCTTCGGTTTGGCTG	GTAAACAAGTGGGAGGGCGA	This paper
eIF3-S9	CTTTCACCTTCCATGACGAGAC	GGACTCCACCAACGATTACATC	This paper
Smox	GATCGCCGCATTGTAATCGG	TCCATCCTGGTGCCCAAATC	This paper
U2af50	AATTTCCACGCTCCGCAC	CCGACTGAGGTTCTTTGCTTG	This paper
blw	TACCGAAGACCACAACACCG	GCCGATGAGATGGTGGAGTT	This paper
ana1	AGGCAGTGGAGTTGGAATCA	TCACGGAAAGCGACCATTCT	This paper
SPARC	TGCAGCACAATCTACTCAATCC	GTAGAATCGAGACCGAGGAGAG	(Gaddam *et al*, 2013)
mtrm	ATGCCAGTTCCACCAGATCG	CGTCGTCGTTCCATCAAAGC	This paper
l(2)k09022	GCAGTTGCCTTCTCTGGTTC	CAGCGCAAGAGTGAGTTGAG	This paper
Nopp140	CTTGCCGTTTGTTAGTGCAG	AGATCCCAGCAATCAAGGC	This paper
RpL5	GAGCTGCAATCTTAGTCTTCCG	CACCAGGCAATTCGTAACG	This paper
l(2)35Di	TTGATGGGGTTGTTCAGCTC	TGAAGCCGATGGTAATTGCG	This paper
Dredd	CGCCTCCACATTGTATCCCA	CCAGCAGAAGTTTCACCGGA	This paper
Hakai	GAAGGTGCTGAACCCGATGAT	GAGGCAAAATACATGCTTGCAG	This paper
CG12262	CTCCACAATGAACCCGGTGA	AGTGGGTAATCAACGGCCAG	This paper
CG2862	GCATGAGATGTCCCAGCA	AAATGCGTTGCCTTCCAC	This paper
Spn42Da	GAACCTGGGCAATTTGAGCG	GATCGTACTGCCCAACACCA	This paper
rp49 (calibrator)	GCTAAGCTGTCGCACAAATG	GTTCGATCCGTAACCGATGT	(Kohyama-Koganeya *et al*, 2008)
Human			
MOB3A	ATCCAGTCCATCAGCAGGTC	ACCTCATCTACGGCACCATC	This paper
JKAMP	AACCAGAGGAAGCATTGCCA	GGACAGAGAACGAATGCACAG	This paper
SPARC	TCCAGGCGCTTCTCATTCTC	GCTCAAGAACGTCCTGGTCA	This paper
FIS1	CGTACTCAAACTGCGTGCTC	CTGAACGAGCTGGTGTCTGT	This paper
HEATR1	GGTCTGAACACTTCGCTCCA	TACTGGCCTGGAAGAGTTGC	This paper
YME1L	ATTCTCCAACTCTGCTCCG	GCAAGTTACAGTTCCAAGGC	This paper
SMAD3	TGGTGGTCACTGGTTTCTCC	CACTGGACGACTACAGCCAT	This paper
KPNA6	CTTGCCCTCTTGCTCTCCAA	TCTCACTGGGCTGCATCAAA	This paper

WDR12	CCATGTGTTTGTCCAAGGGC	CTGCTGCCTCTGAAATTGCC	This paper
ILK	ATGAGGAGCAGGTGGAGACT	TGCTGAAGGTTCGAGACTGG	This paper
ATP5A1	TGAAGAGGACAGGAGCCATTG	ACCAAGGGCATCAACTACAC	This paper
SPSB1	AGAACCAGCCAAGCAAAACA	AAGCCACTCCCATGTACTGT	This paper
TMEM33	TGCTCTGAGGCTGCATCAAA	AACAGGTAGTGGCAGCTGTC	This paper
HINT1	TTTGGCTGGTATTTCCTTGC	GCGGGTTCCTCCCTTCTT	(Hua *et al*, 2011)
SERPINI1	GAGCTGGGCACTCAGAATGT	GGACACCCTCAGCATGTTCA	This paper
PRPF3	ACAGATGATGGAAGCAGCAA	GGTTGGGAGGATGAAGGAGT	This paper
ATP5C1	GAGCTGCAGCACCAGAGATA	GAGCAGAGTGCCAGGATGA	This paper
ACADM	AAATCATCCCAGTGGCTGCA	ACCAAGTTCCCAGGCTCTTC	This paper
CBLL1	CGCAGACGAATTCCTATAAAGC	CCTTCTTCATCACCAGGTGG	This paper
**TRIM3*	GGCTGACTGGGGCAACAGCCGCATC	ATCTGCCAGAACCACTGTATGGTCCA	(Chen *et al*, 2014)
47S pre-rRNA (5'ETS)	CCTGCTGTTCTCTCGCGCGTCCGAG	AACGCCTGACACGCACGGCACGGAG	(Grandori *et al*, 2005)
18S	AAACGGCTACCACATCCAAG	CCTCCAATGGATCCTCGTTA	(Karahan *et al*, 2015)
5.8S	CTCTTAGCGGTGGATCACTC	GACGCTCAGACAGGCGTAG	(Karahan *et al.*, 2015)
28S	CAGGGGAATCCGACTGTTTA	ATGACGAGGCATTTGGCTAC	(Karahan *et al.*, 2015)
MYC	CCTCTCAACGACAGCAGCT	CAGAAGGTGATCCAGACTCTG	(Rossetti *et al*, 2018)
RRN3	CGGAAACCTGAAAGAAGGTTTGC	CTGGCGATTGTTCCTCTCAATG	(Rossetti *et al.*, 2018)
POLR1B	TATGGAAGATGCCATGATTGTGA	TGTAATACGGATCTCCGTACTG	(Rossetti *et al.*, 2018)
RPL32 (calibrator)	CATCTCCTTCTCGGCATCA	AACCCTGTTGTCAATGCCTC	(Reber *et al*, 2006)

Appendix Table S2. Primers used for PCR and RT-qPCR gene expression analysis.
*Reverse primer contains an extra cytosine.
