# Supplementary figures and images for: Ribogenesis boosts controlled by HEATR1-MYC interplay promote transition into brain tumour growth
Source: EMBO Rep. 2024 Jan 15;25(1):14. doi: 10.1038/s44319-023-00017-1 (PMC10897169; doi:10.1038/s44319-023-00017-1)

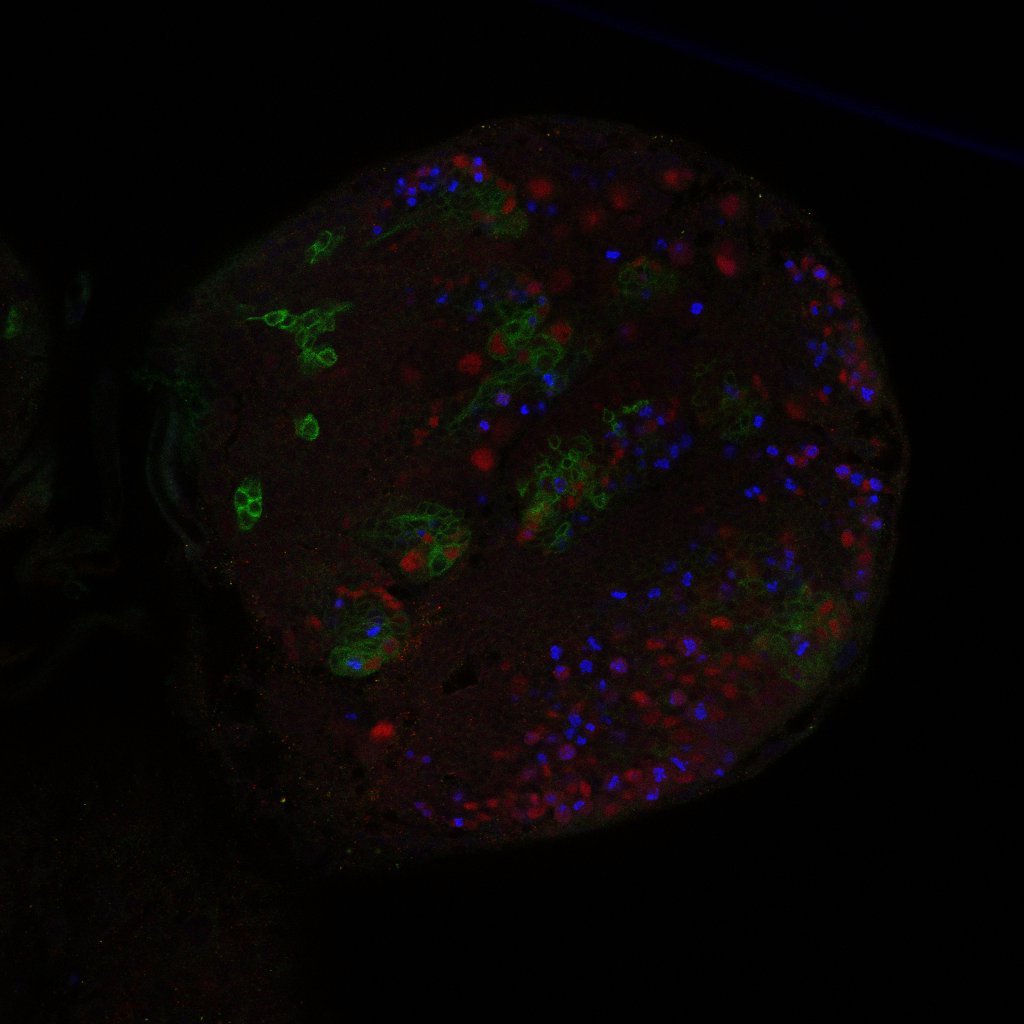

Supplement: Supplementary file 7 — Source Data Fig. 2 [file 44319_2023_17_MOESM7_ESM.zip › Fig2_source data/Fig2R_source data.jpg]

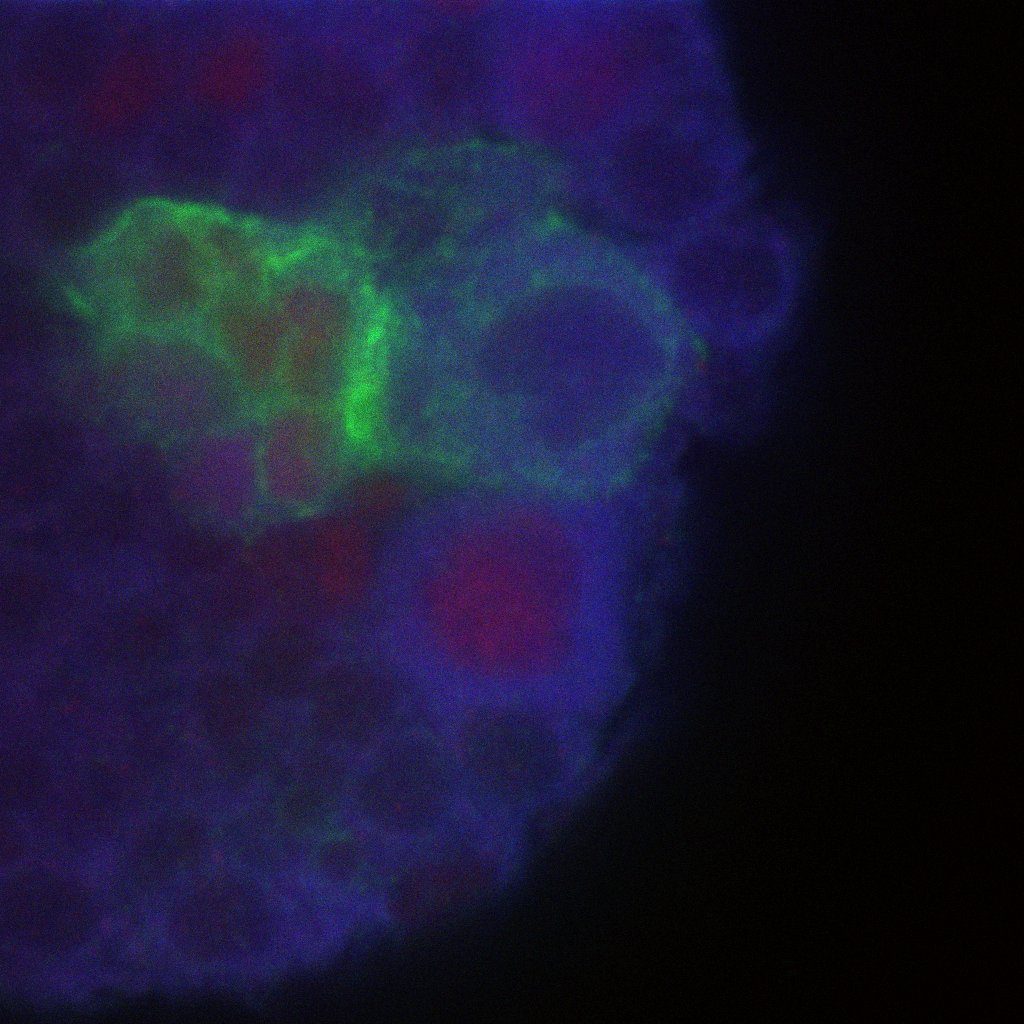

Supplement: Supplementary file 7 — Source Data Fig. 2 [file 44319_2023_17_MOESM7_ESM.zip › Fig2_source data/Fig2G_source data.jpg]

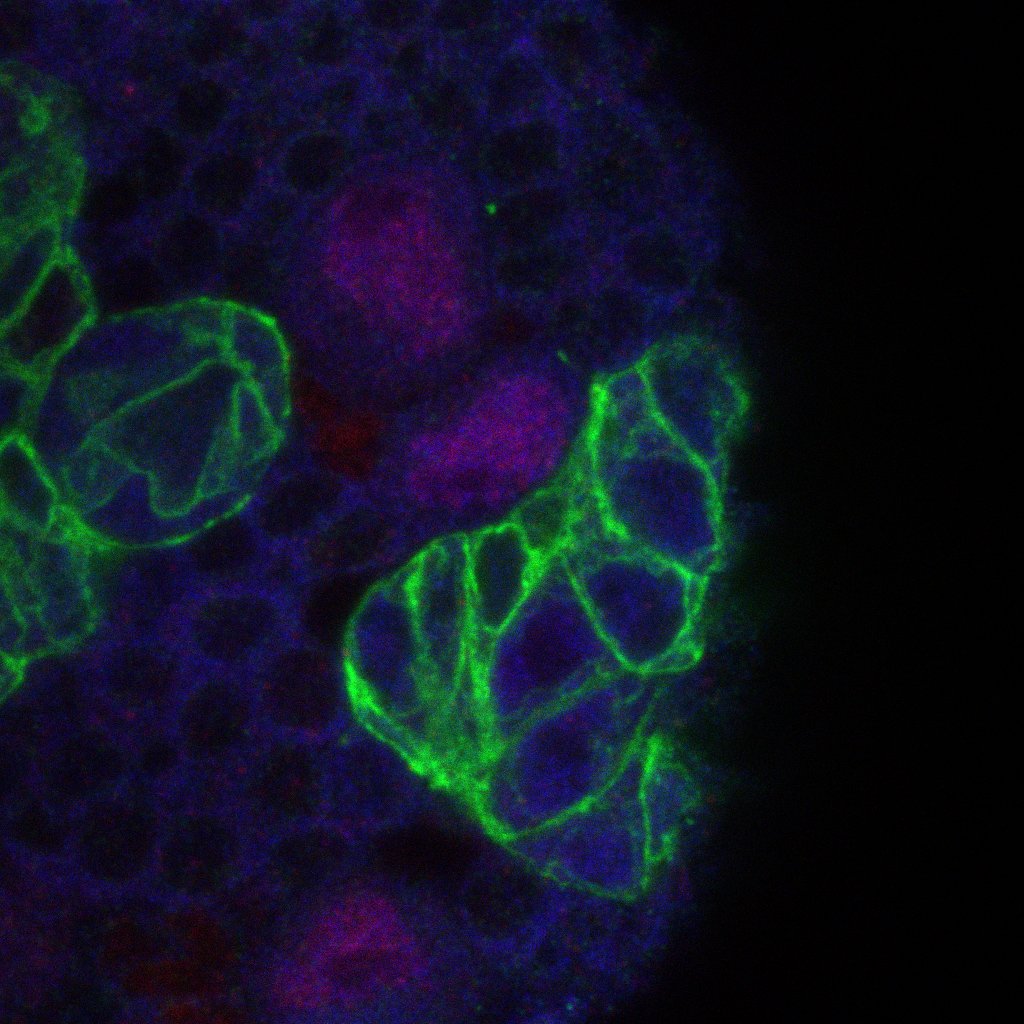

Supplement: Supplementary file 7 — Source Data Fig. 2 [file 44319_2023_17_MOESM7_ESM.zip › Fig2_source data/Fig2H_source data.jpg]

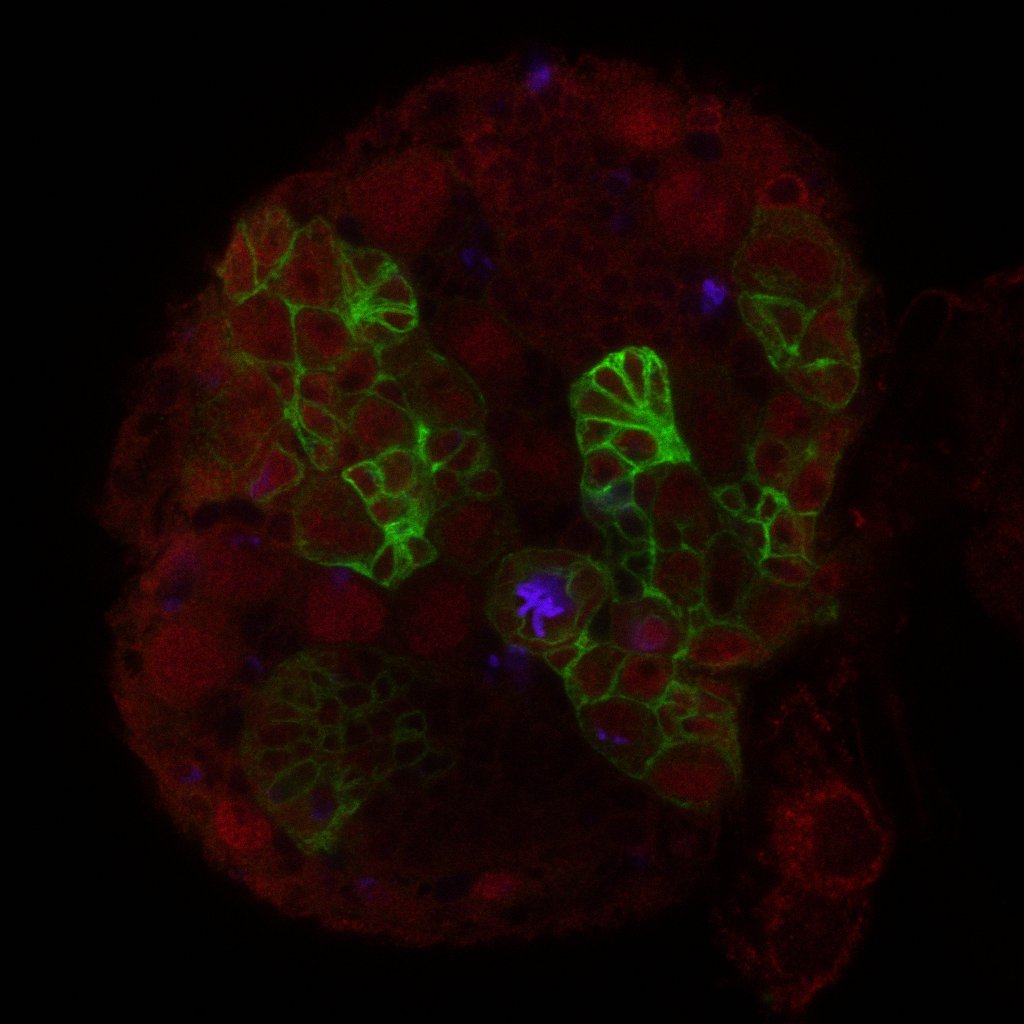

Supplement: Supplementary file 7 — Source Data Fig. 2 [file 44319_2023_17_MOESM7_ESM.zip › Fig2_source data/Fig2O_source data.jpg]

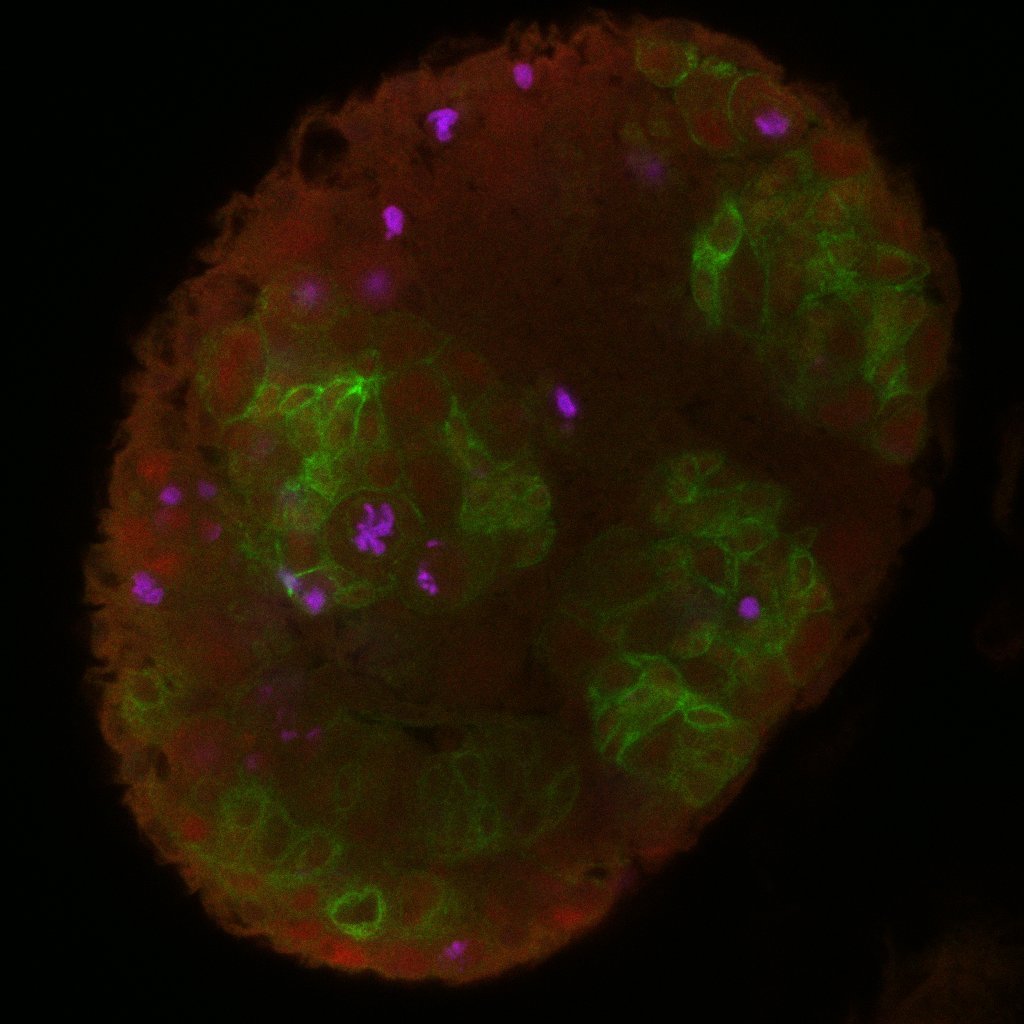

Supplement: Supplementary file 7 — Source Data Fig. 2 [file 44319_2023_17_MOESM7_ESM.zip › Fig2_source data/Fig2N_source data.jpg]

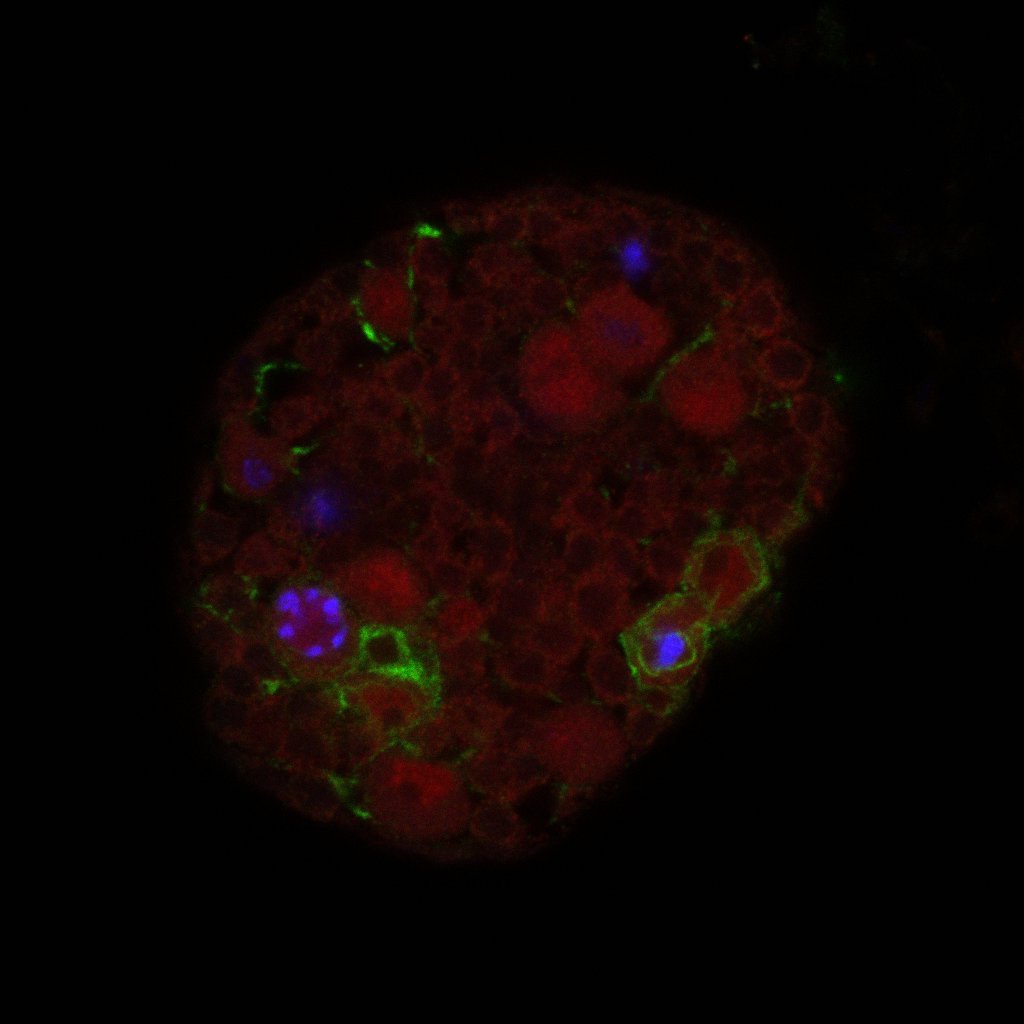

Supplement: Supplementary file 7 — Source Data Fig. 2 [file 44319_2023_17_MOESM7_ESM.zip › Fig2_source data/Fig2A_source data.jpg]

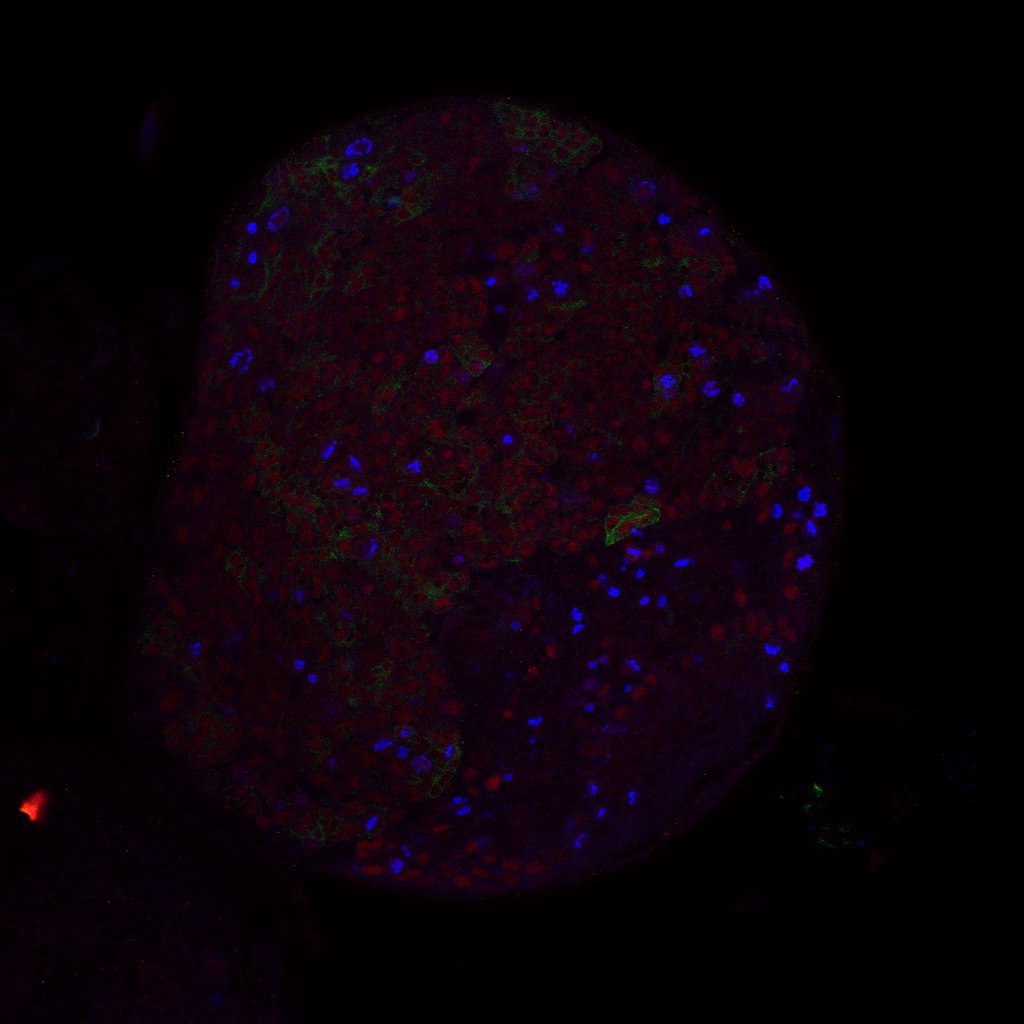

Supplement: Supplementary file 7 — Source Data Fig. 2 [file 44319_2023_17_MOESM7_ESM.zip › Fig2_source data/Fig2T_source data.jpg]

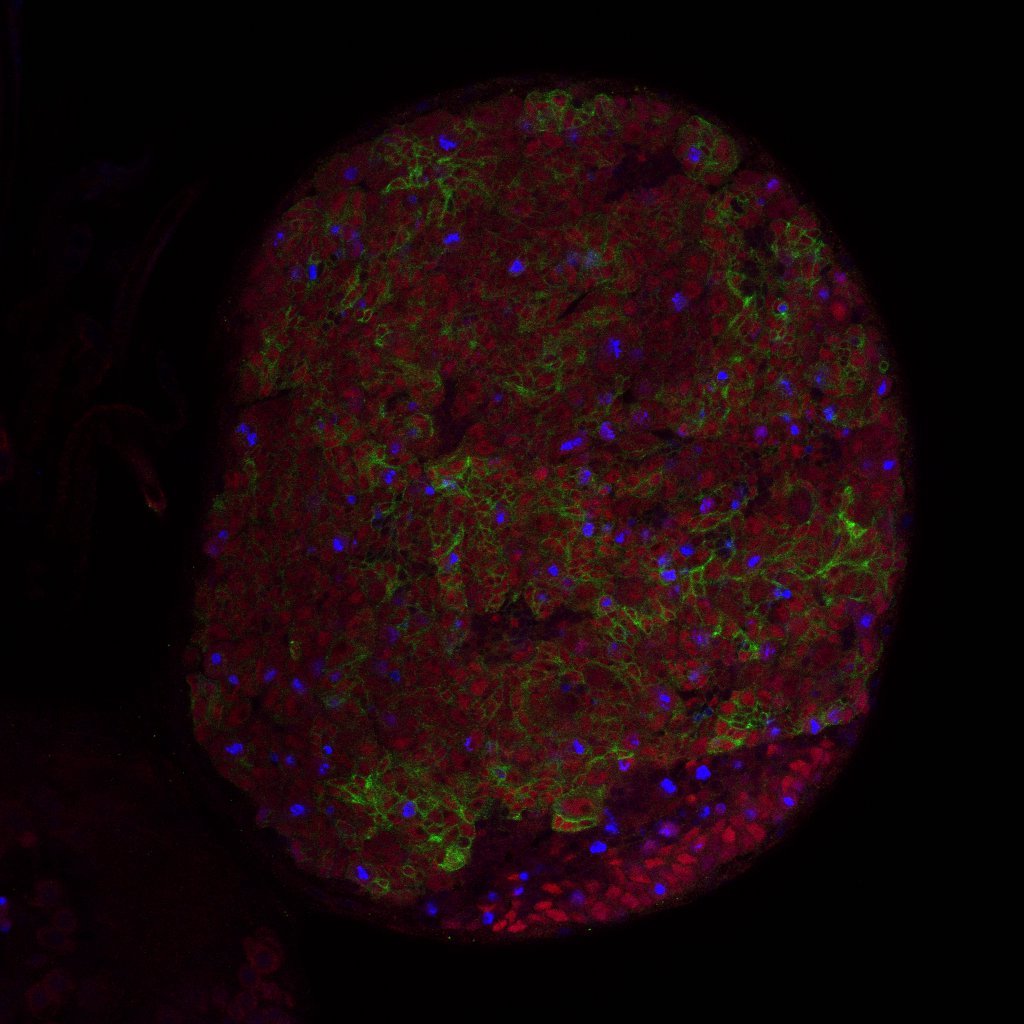

Supplement: Supplementary file 7 — Source Data Fig. 2 [file 44319_2023_17_MOESM7_ESM.zip › Fig2_source data/Fig2S_source data.jpg]

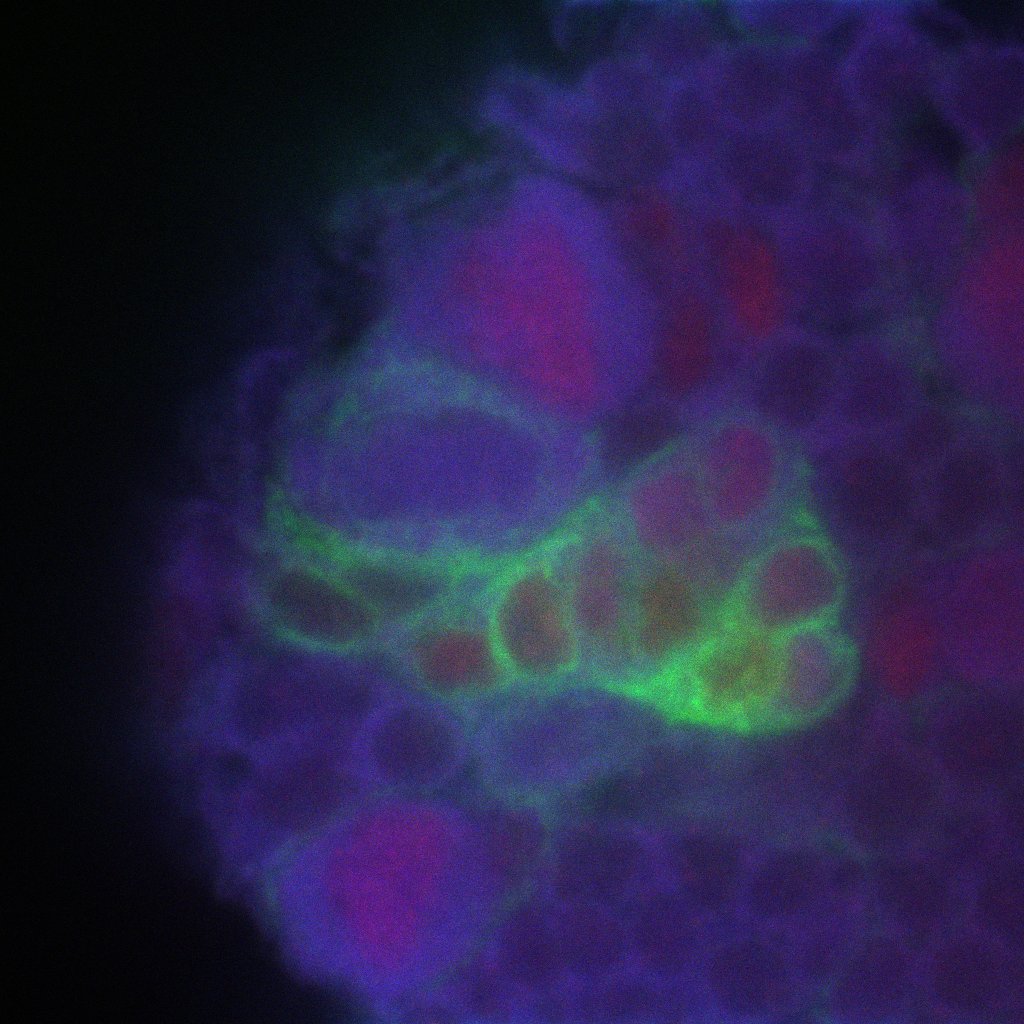

Supplement: Supplementary file 7 — Source Data Fig. 2 [file 44319_2023_17_MOESM7_ESM.zip › Fig2_source data/Fig2F_source data.jpg]

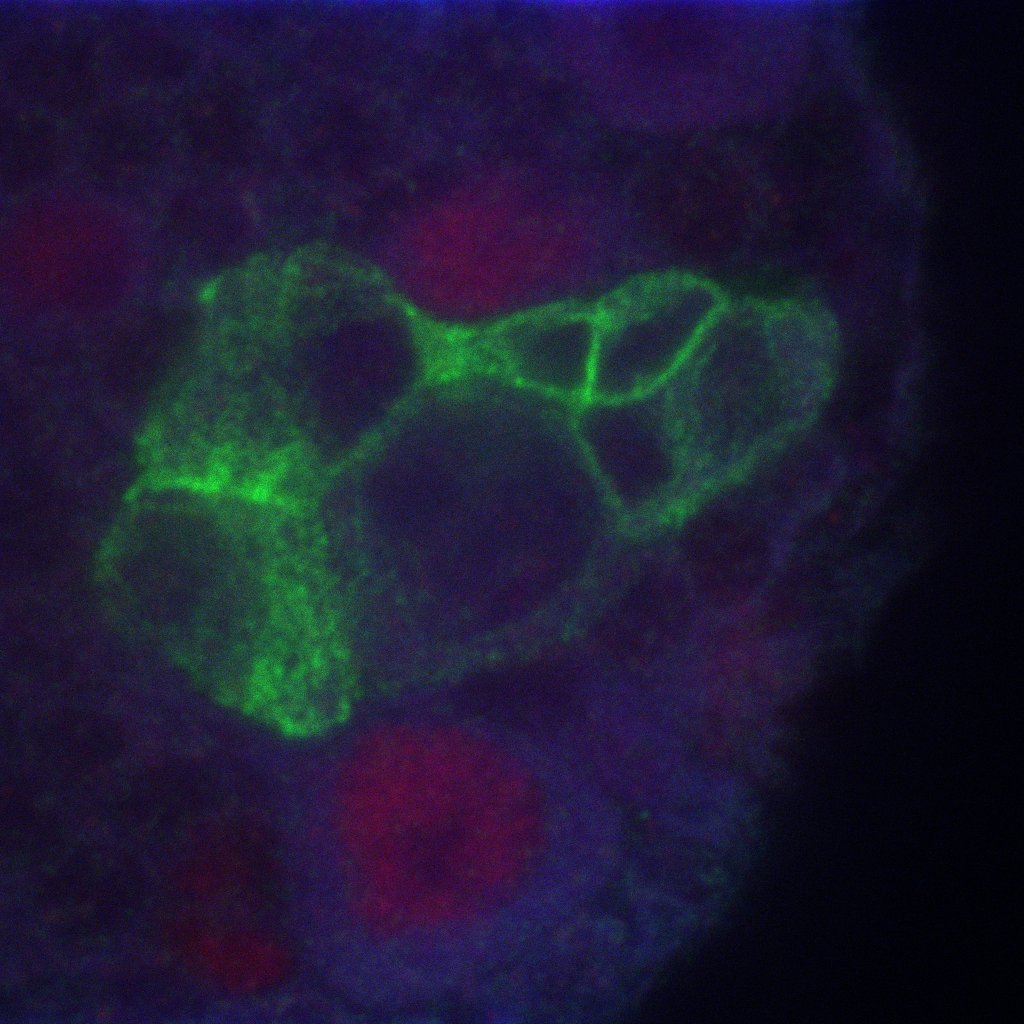

Supplement: Supplementary file 7 — Source Data Fig. 2 [file 44319_2023_17_MOESM7_ESM.zip › Fig2_source data/Fig2I_source data.jpg]

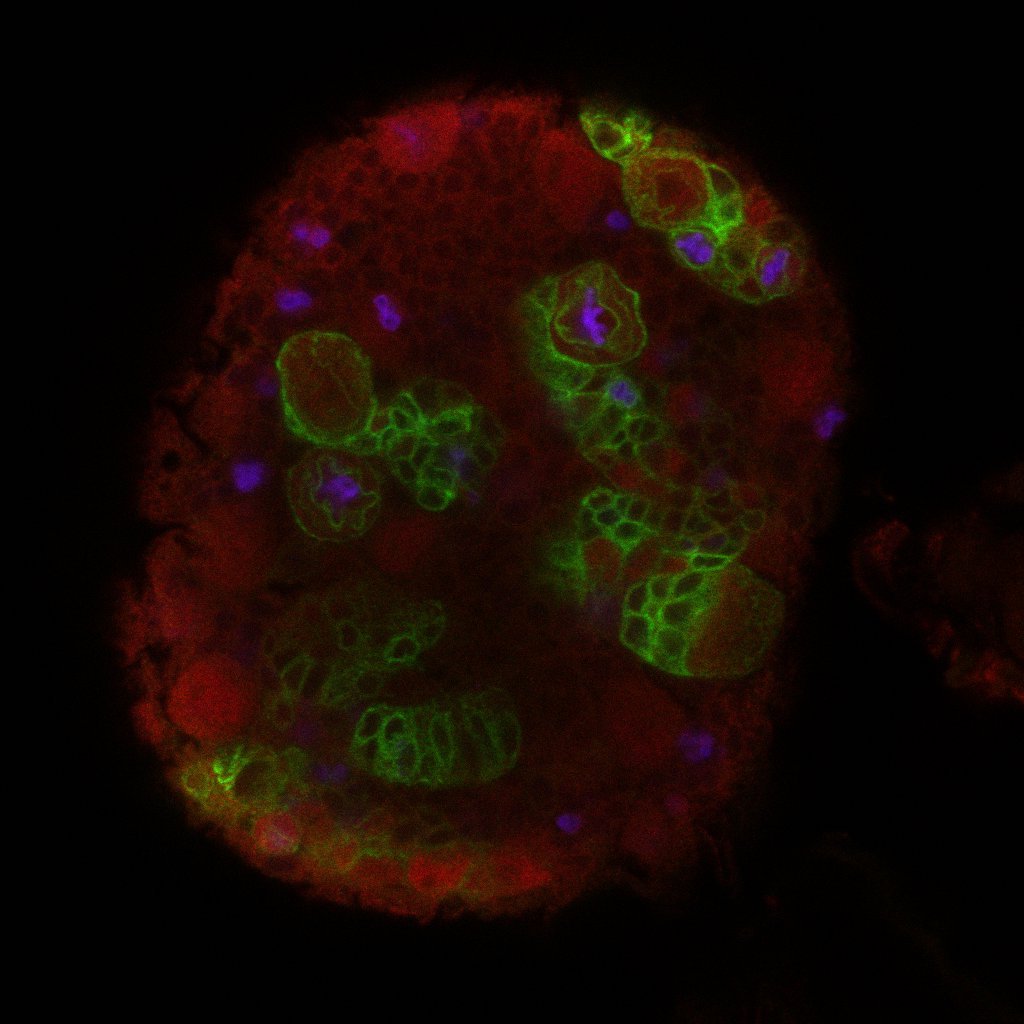

Supplement: Supplementary file 7 — Source Data Fig. 2 [file 44319_2023_17_MOESM7_ESM.zip › Fig2_source data/Fig2L_source data.jpg]

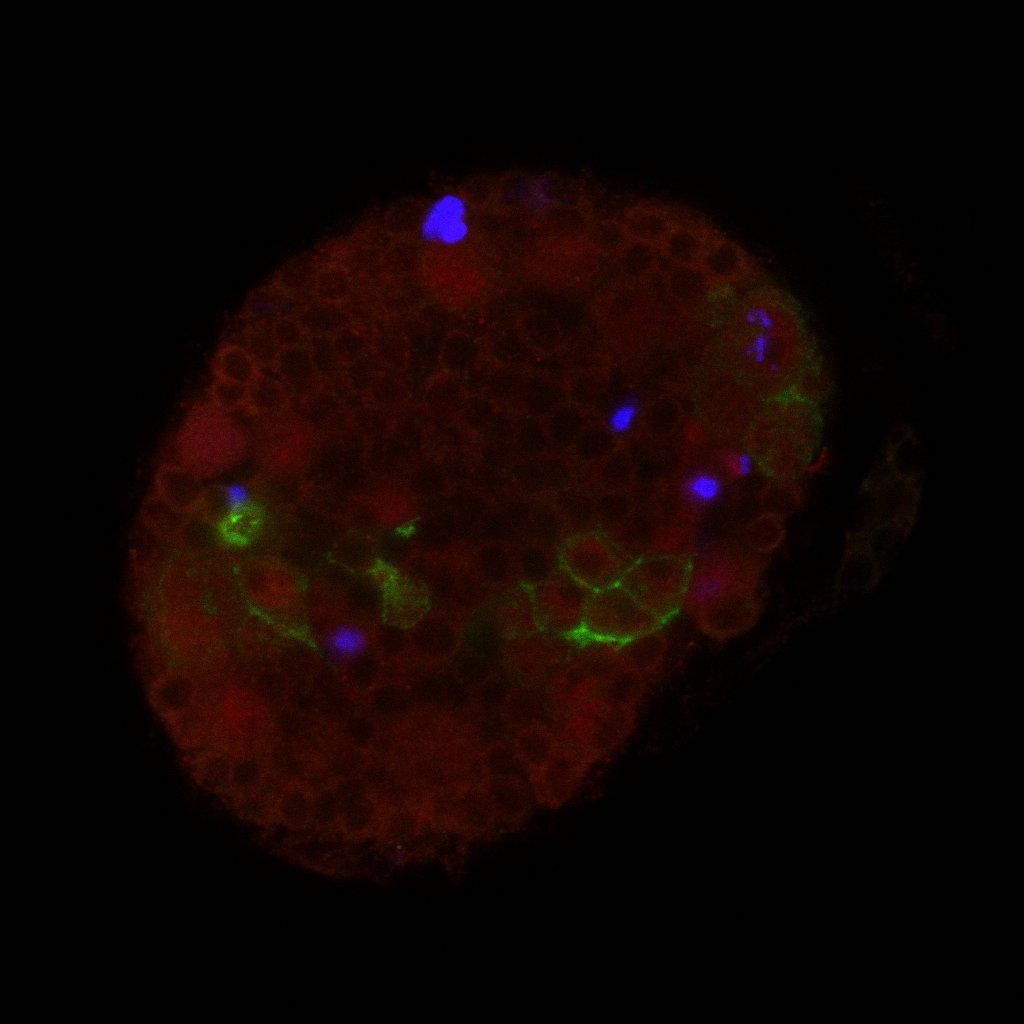

Supplement: Supplementary file 7 — Source Data Fig. 2 [file 44319_2023_17_MOESM7_ESM.zip › Fig2_source data/Fig2C_source data.jpg]

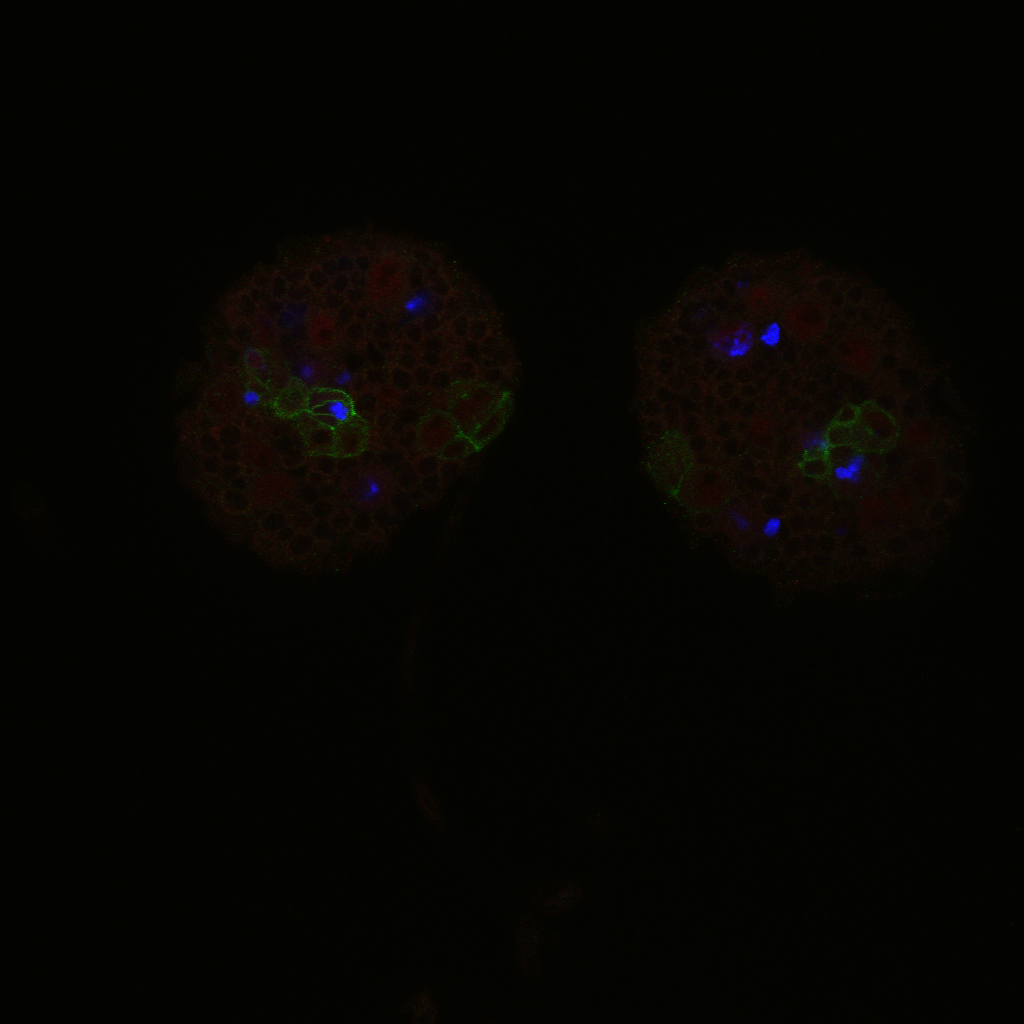

Supplement: Supplementary file 7 — Source Data Fig. 2 [file 44319_2023_17_MOESM7_ESM.zip › Fig2_source data/Fig2D_source data.jpg]

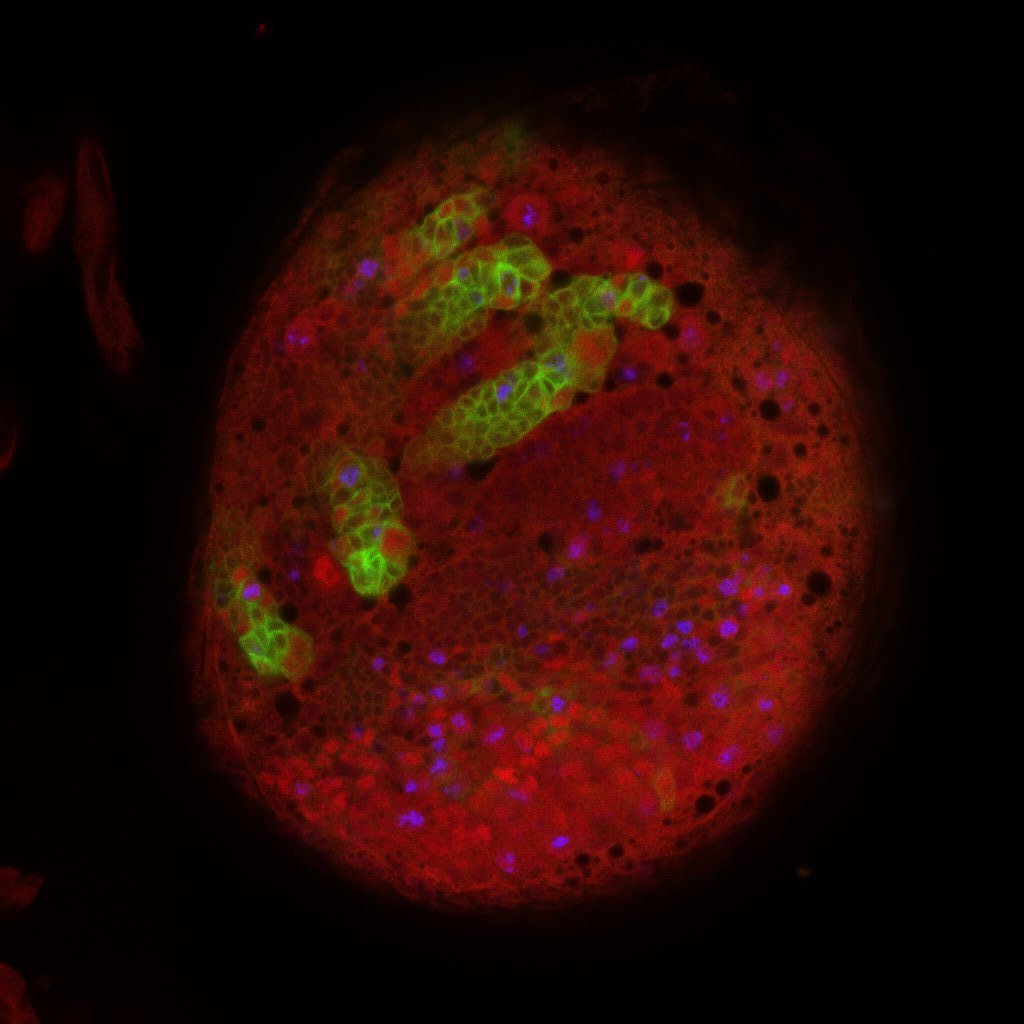

Supplement: Supplementary file 7 — Source Data Fig. 2 [file 44319_2023_17_MOESM7_ESM.zip › Fig2_source data/Fig2Q_source data.jpg]

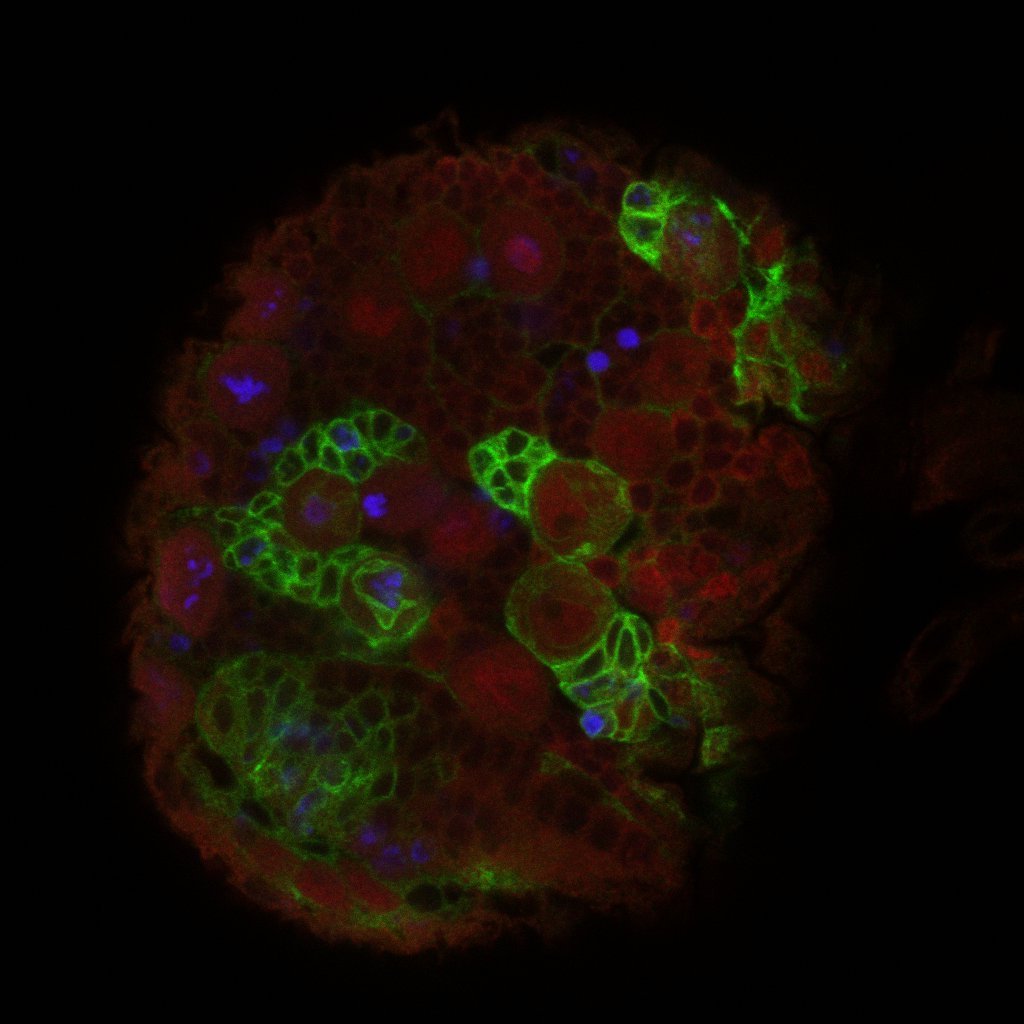

Supplement: Supplementary file 7 — Source Data Fig. 2 [file 44319_2023_17_MOESM7_ESM.zip › Fig2_source data/Fig2M_source data.jpg]

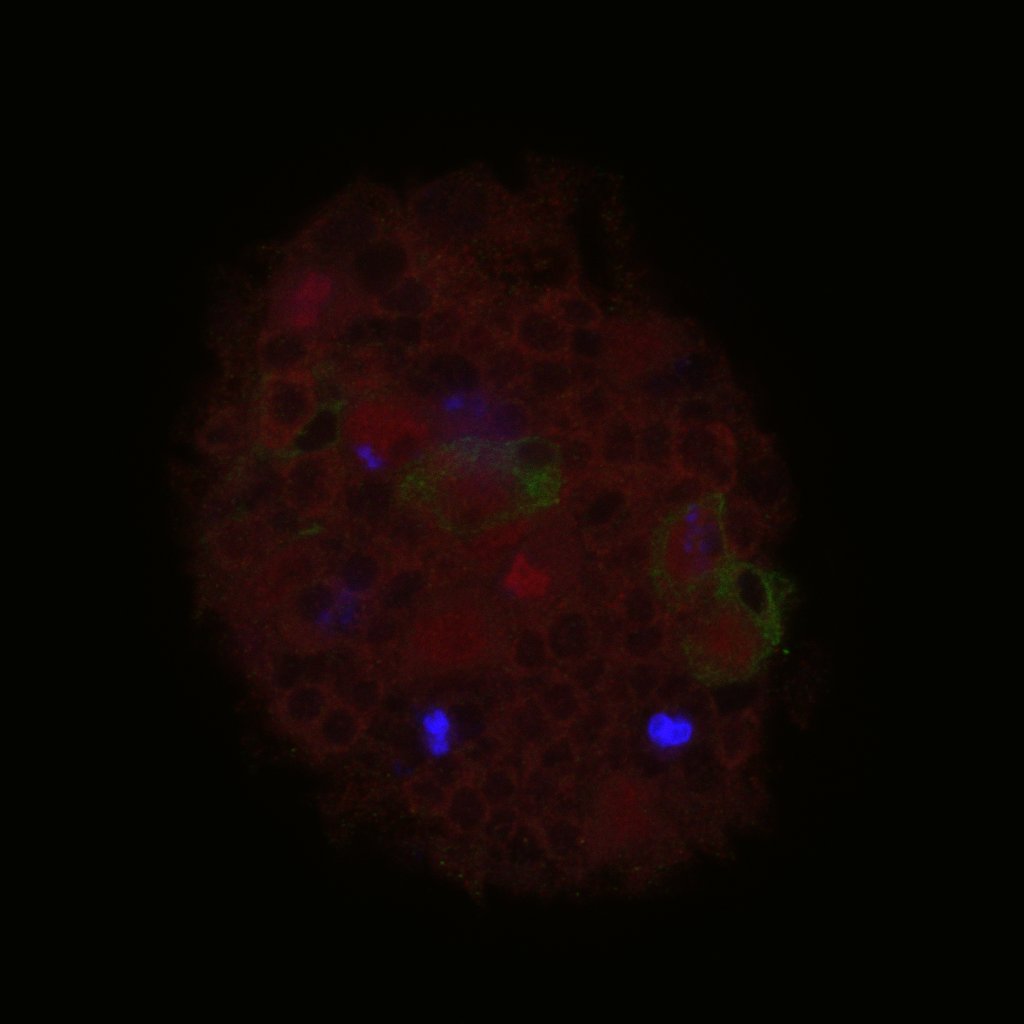

Supplement: Supplementary file 7 — Source Data Fig. 2 [file 44319_2023_17_MOESM7_ESM.zip › Fig2_source data/Fig2B_source data.jpg]

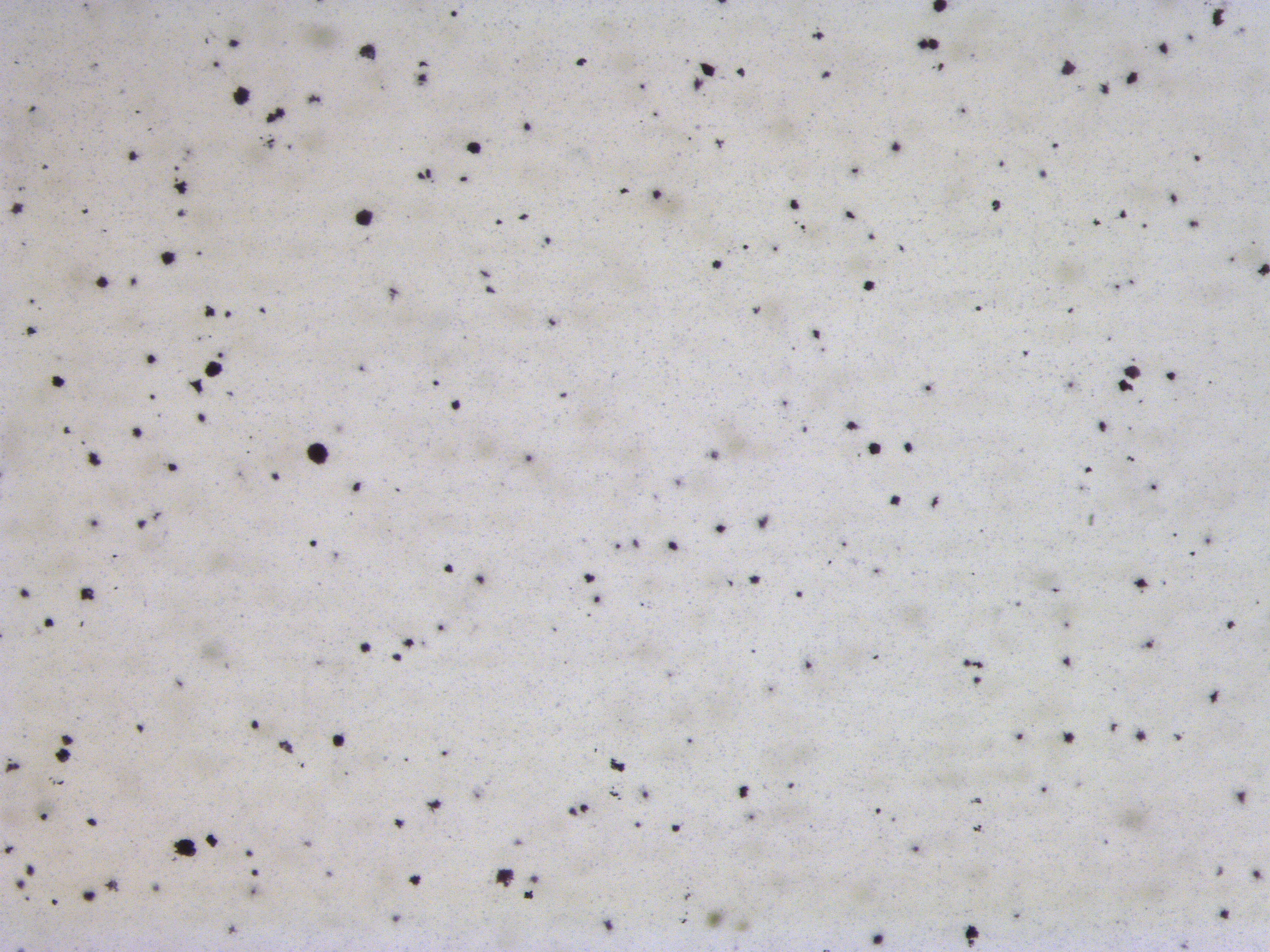

Supplement: Supplementary file 8 — Source Data Fig. 3 [file 44319_2023_17_MOESM8_ESM.zip › Fig3_source data/Fig3N_left panel_source data.tif]

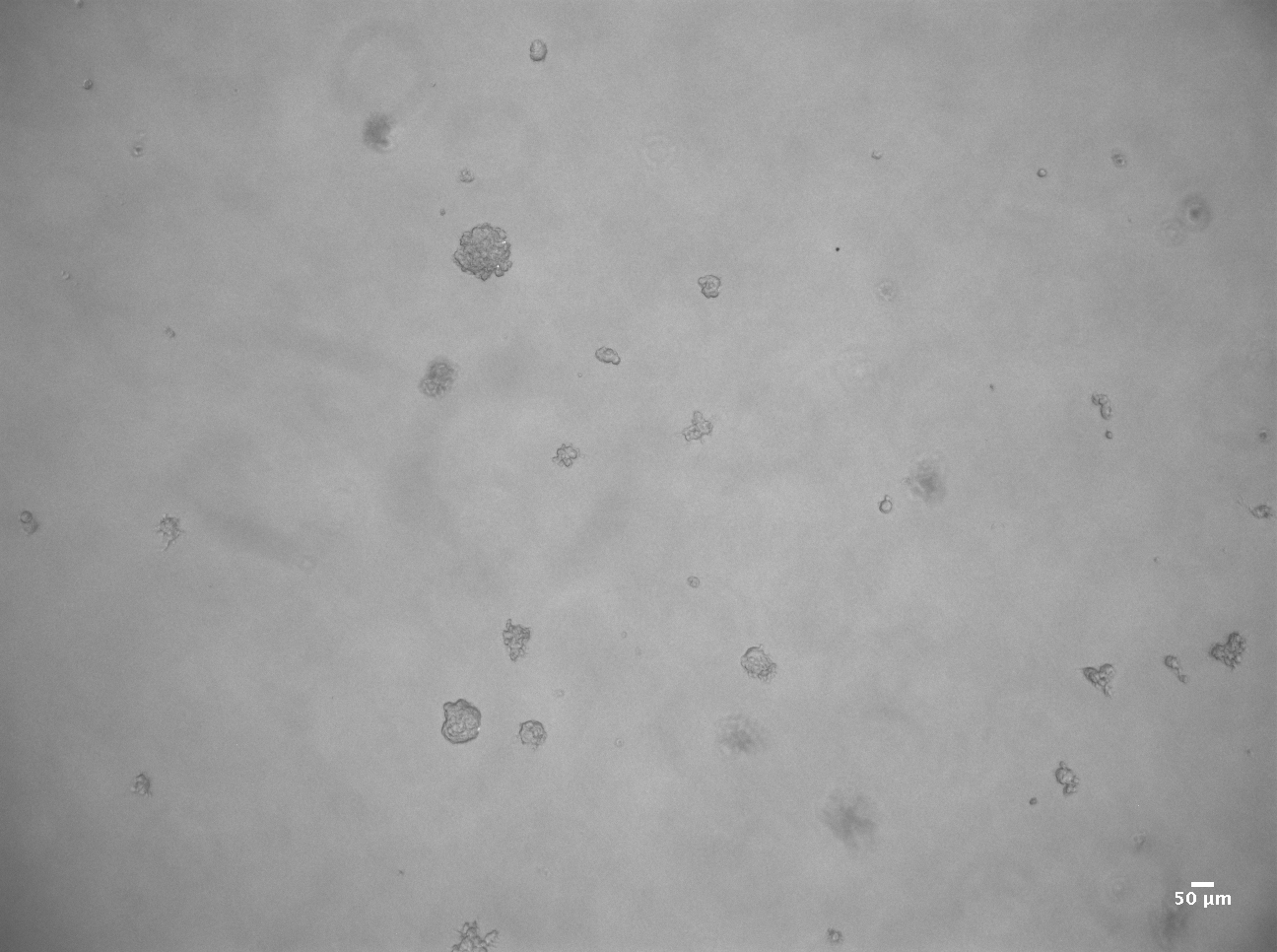

Supplement: Supplementary file 8 — Source Data Fig. 3 [file 44319_2023_17_MOESM8_ESM.zip › Fig3_source data/Fig3N_right panel_source data.tif]

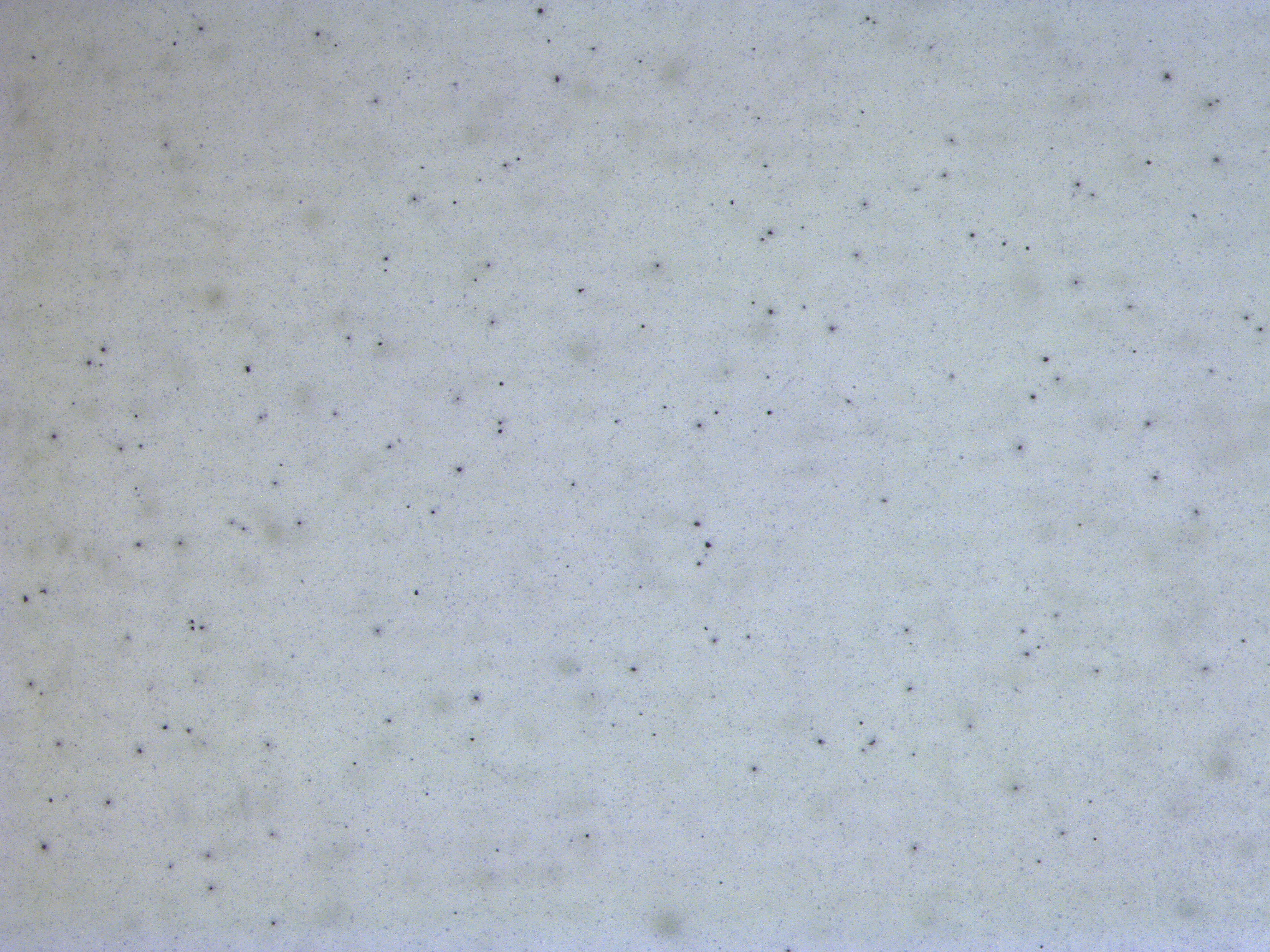

Supplement: Supplementary file 8 — Source Data Fig. 3 [file 44319_2023_17_MOESM8_ESM.zip › Fig3_source data/Fig3R_left panel_source data.tif]

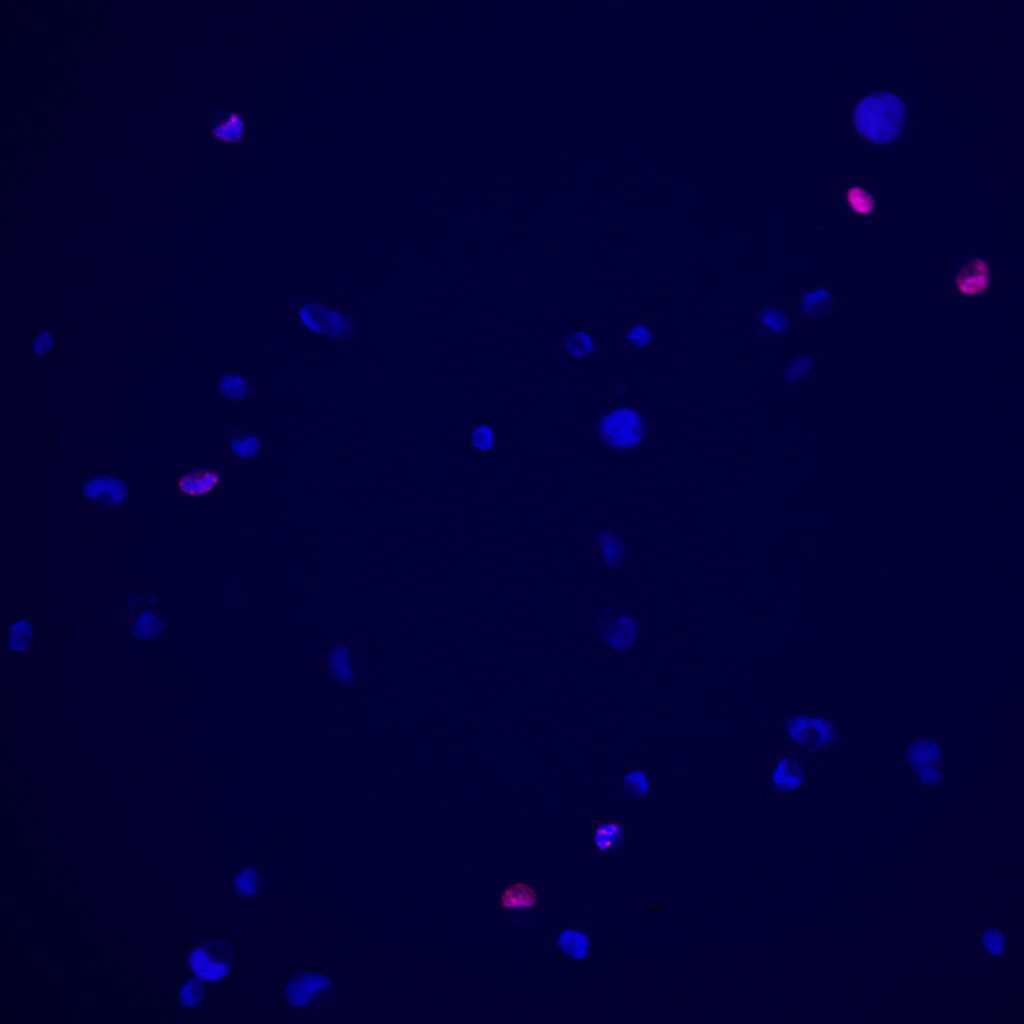

Supplement: Supplementary file 8 — Source Data Fig. 3 [file 44319_2023_17_MOESM8_ESM.zip › Fig3_source data/Fig3G_source data.tif]

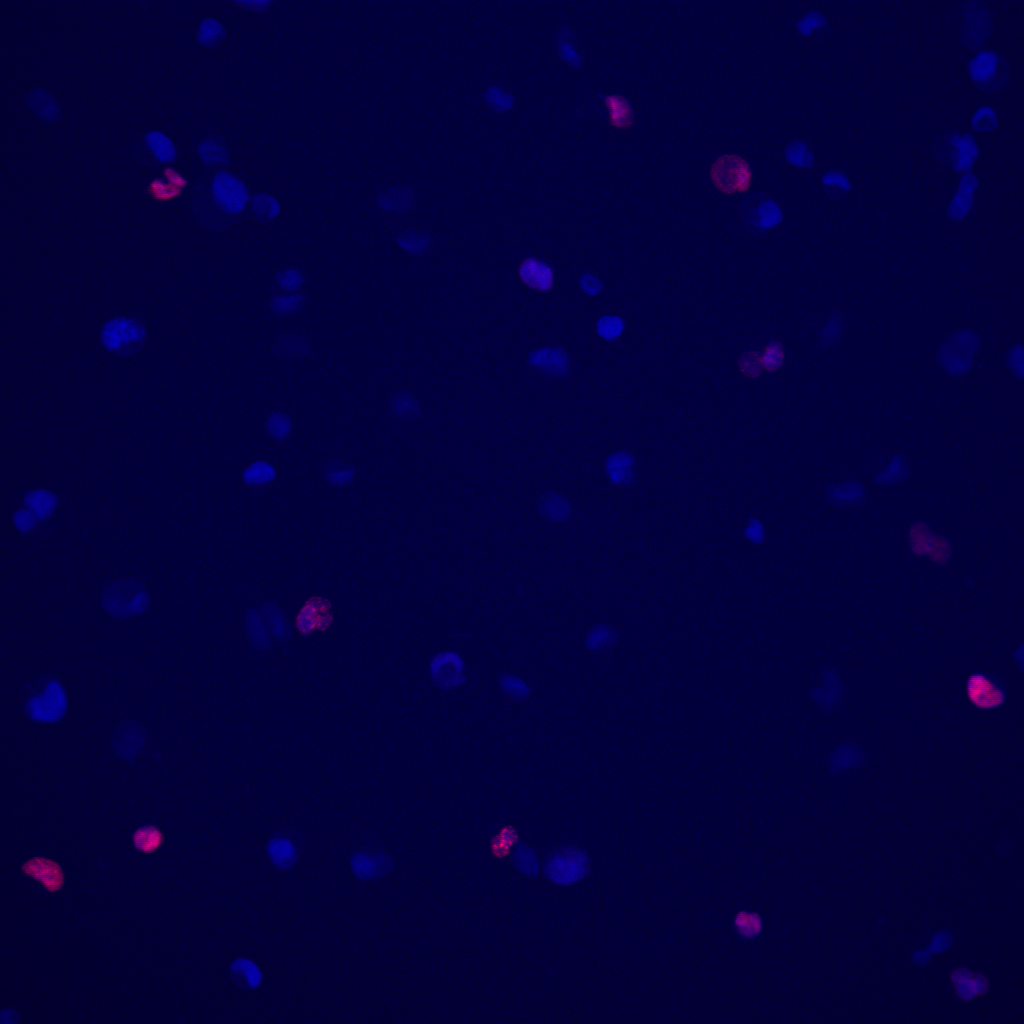

Supplement: Supplementary file 8 — Source Data Fig. 3 [file 44319_2023_17_MOESM8_ESM.zip › Fig3_source data/Fig3H_source data.tif]

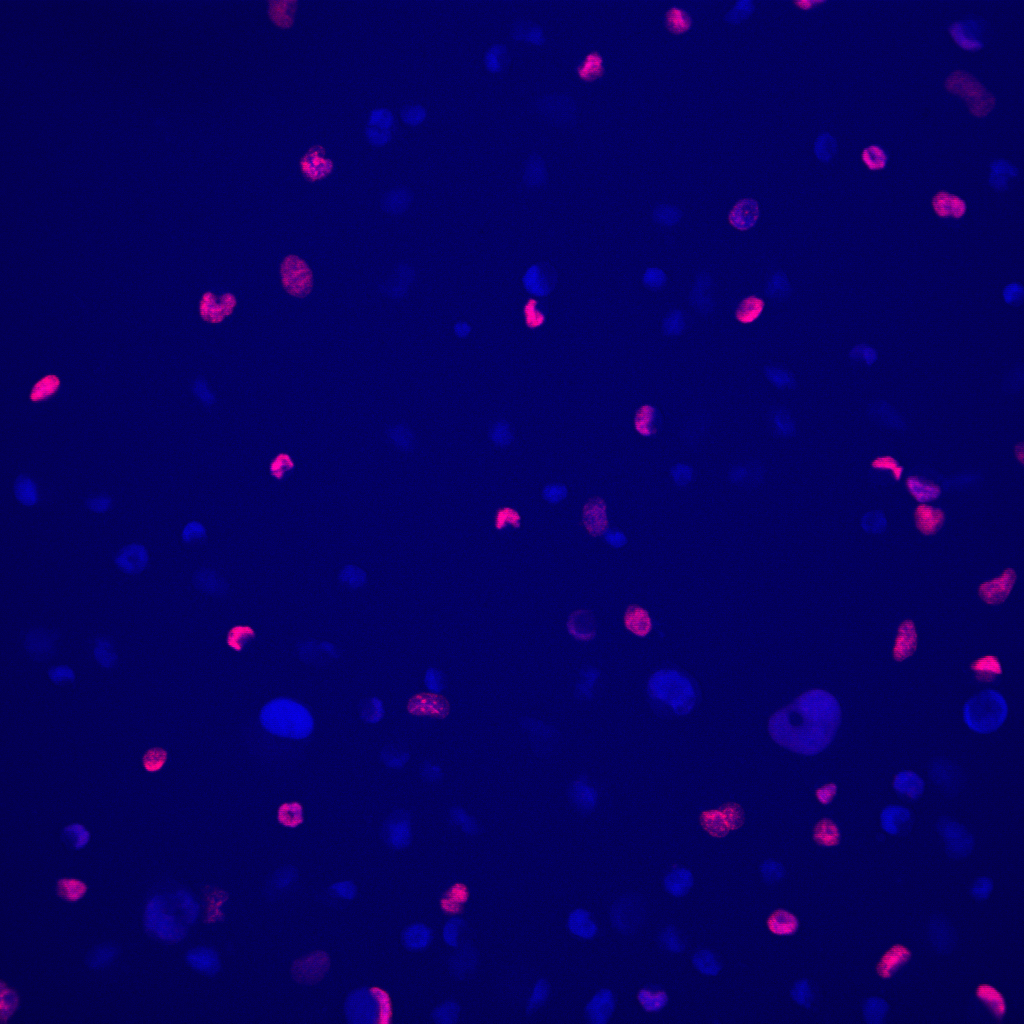

Supplement: Supplementary file 8 — Source Data Fig. 3 [file 44319_2023_17_MOESM8_ESM.zip › Fig3_source data/Fig3F_source data.tif]

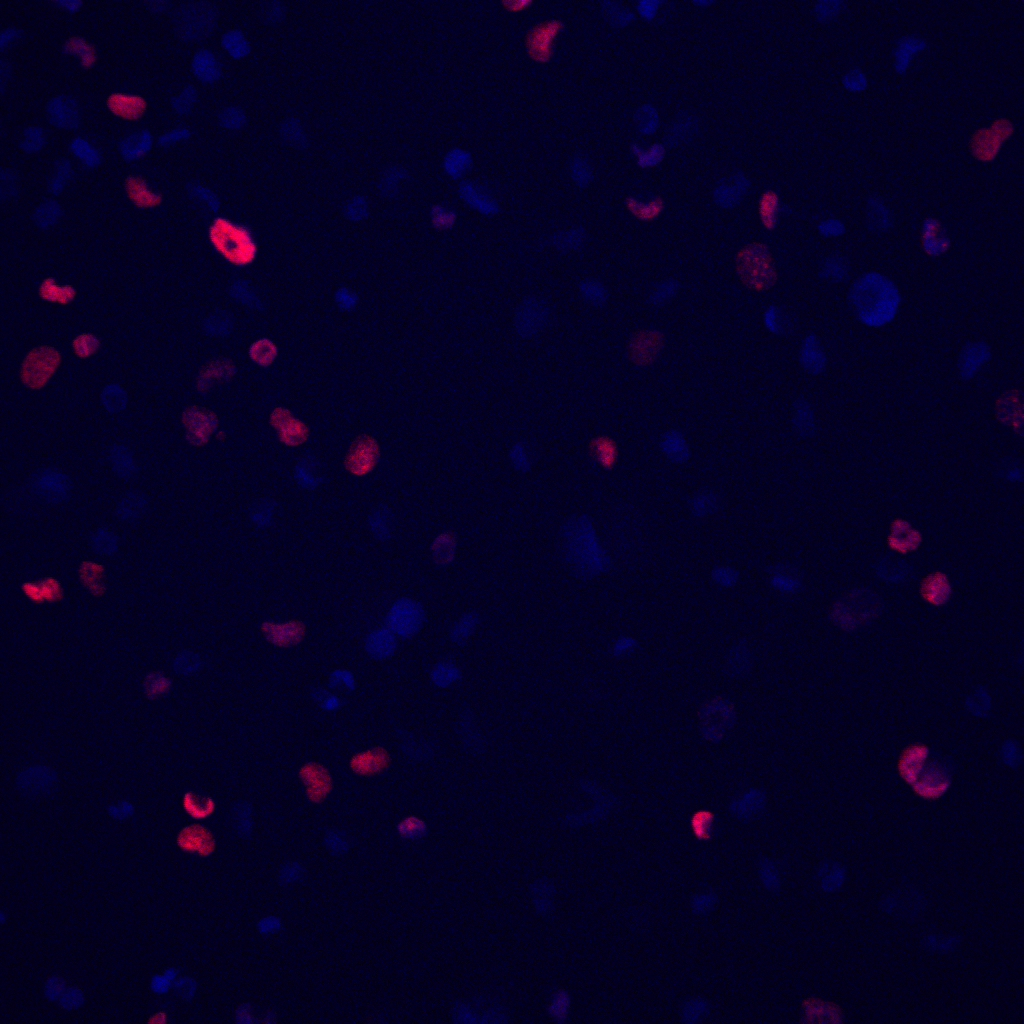

Supplement: Supplementary file 8 — Source Data Fig. 3 [file 44319_2023_17_MOESM8_ESM.zip › Fig3_source data/Fig3I_source data.tif]

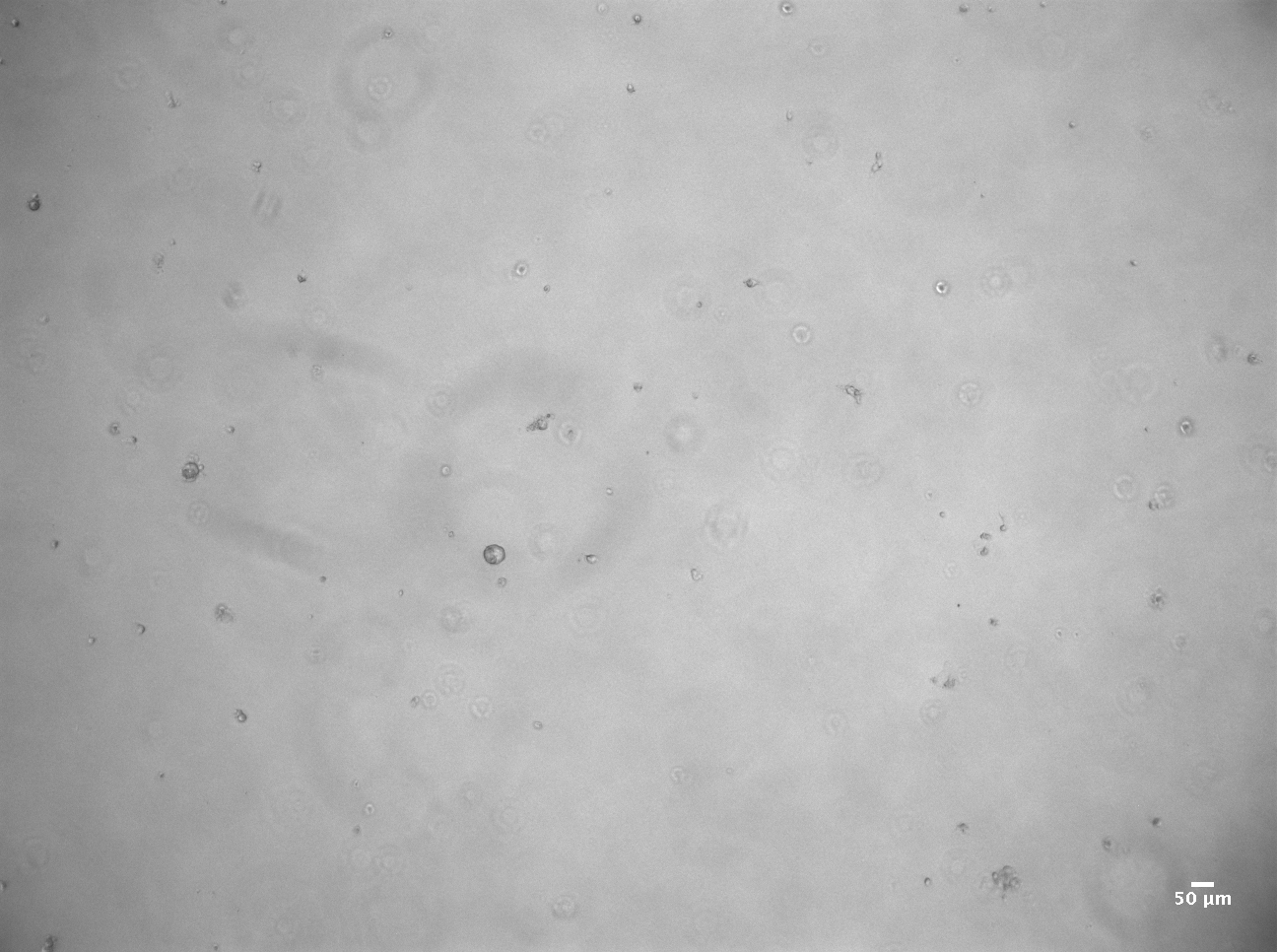

Supplement: Supplementary file 8 — Source Data Fig. 3 [file 44319_2023_17_MOESM8_ESM.zip › Fig3_source data/Fig3O_right panel_source data.tif]

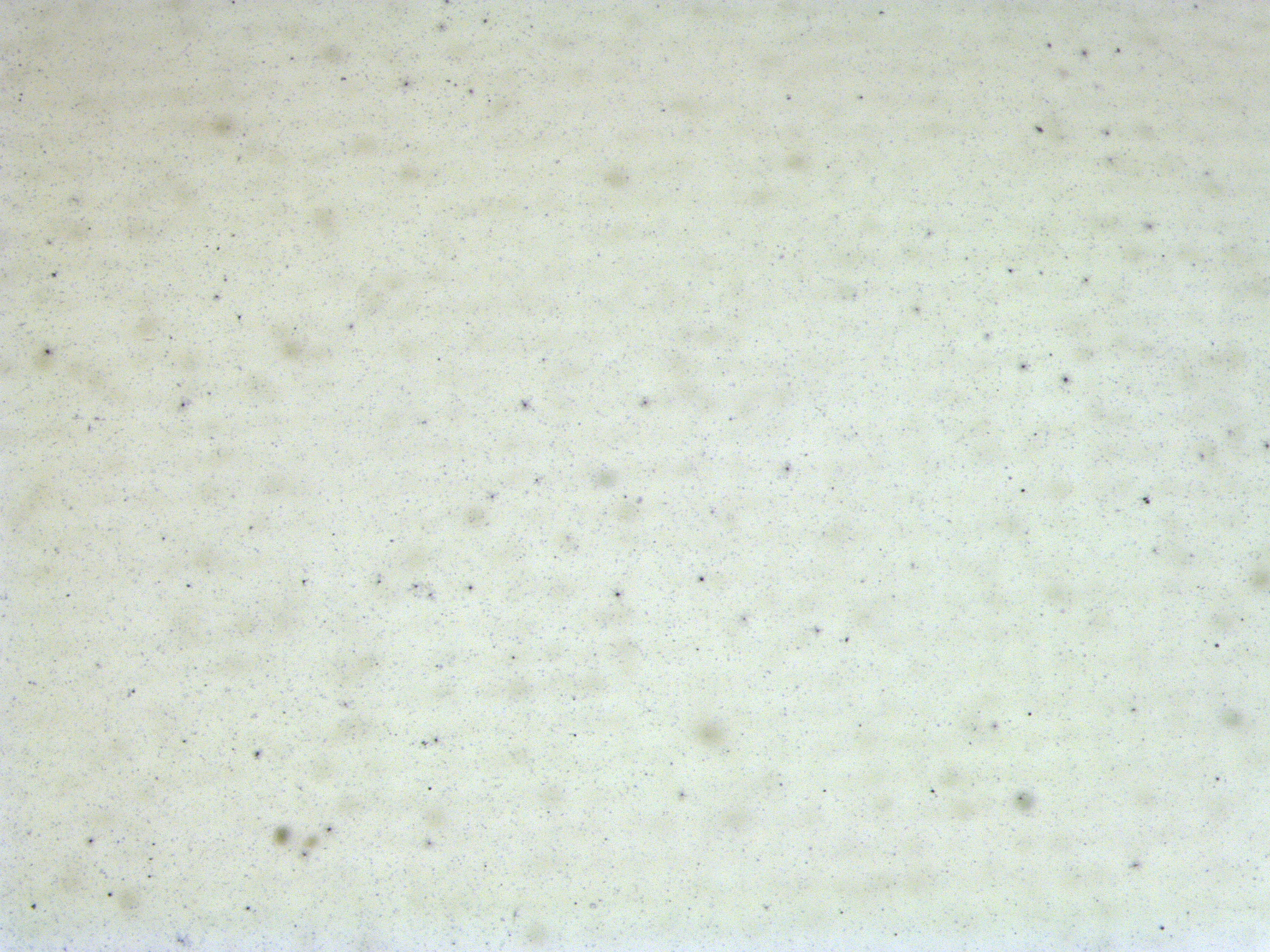

Supplement: Supplementary file 8 — Source Data Fig. 3 [file 44319_2023_17_MOESM8_ESM.zip › Fig3_source data/Fig3O_left panel_source data.tif]

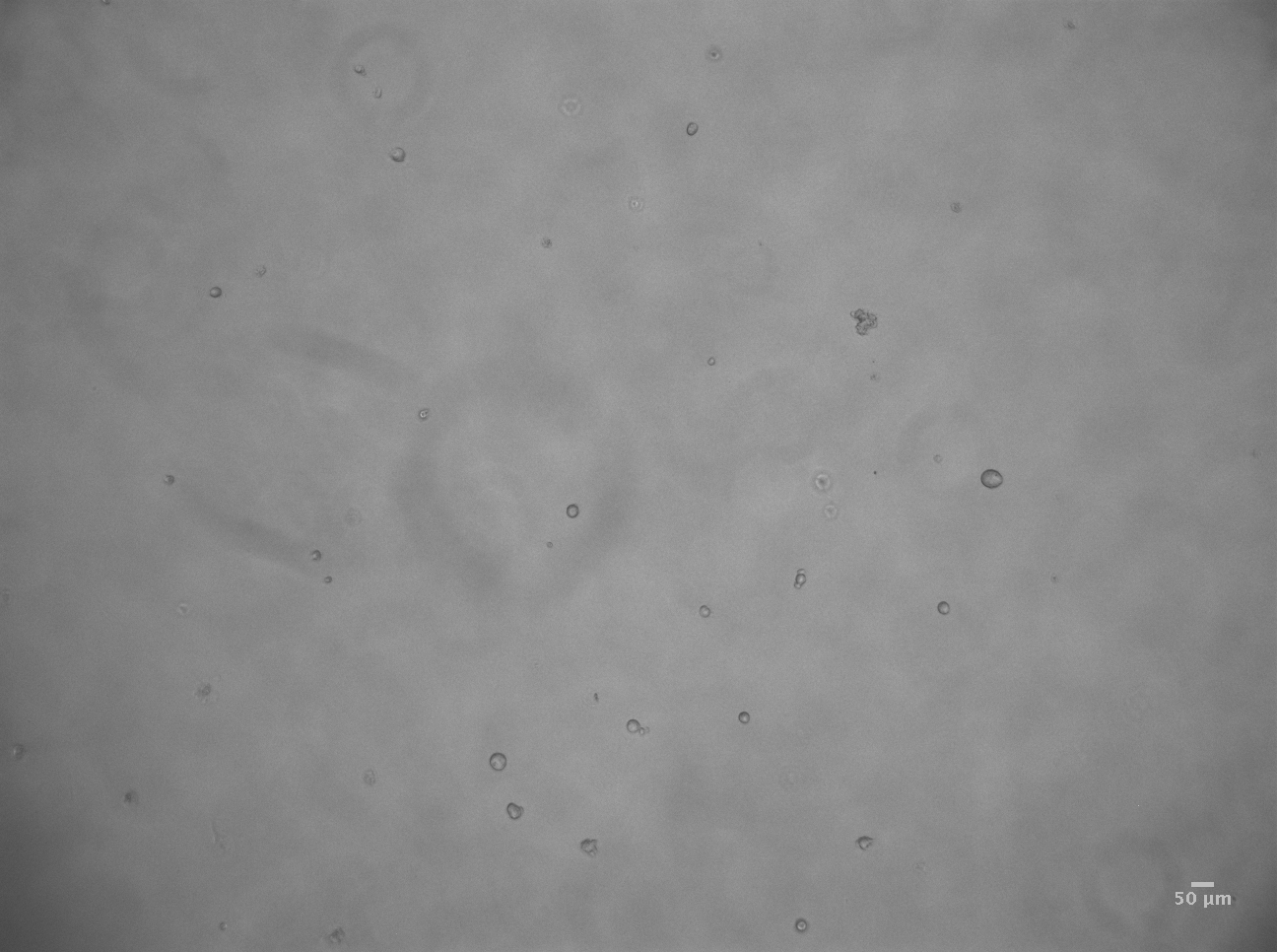

Supplement: Supplementary file 8 — Source Data Fig. 3 [file 44319_2023_17_MOESM8_ESM.zip › Fig3_source data/Fig3R_right panel_source data.tif]

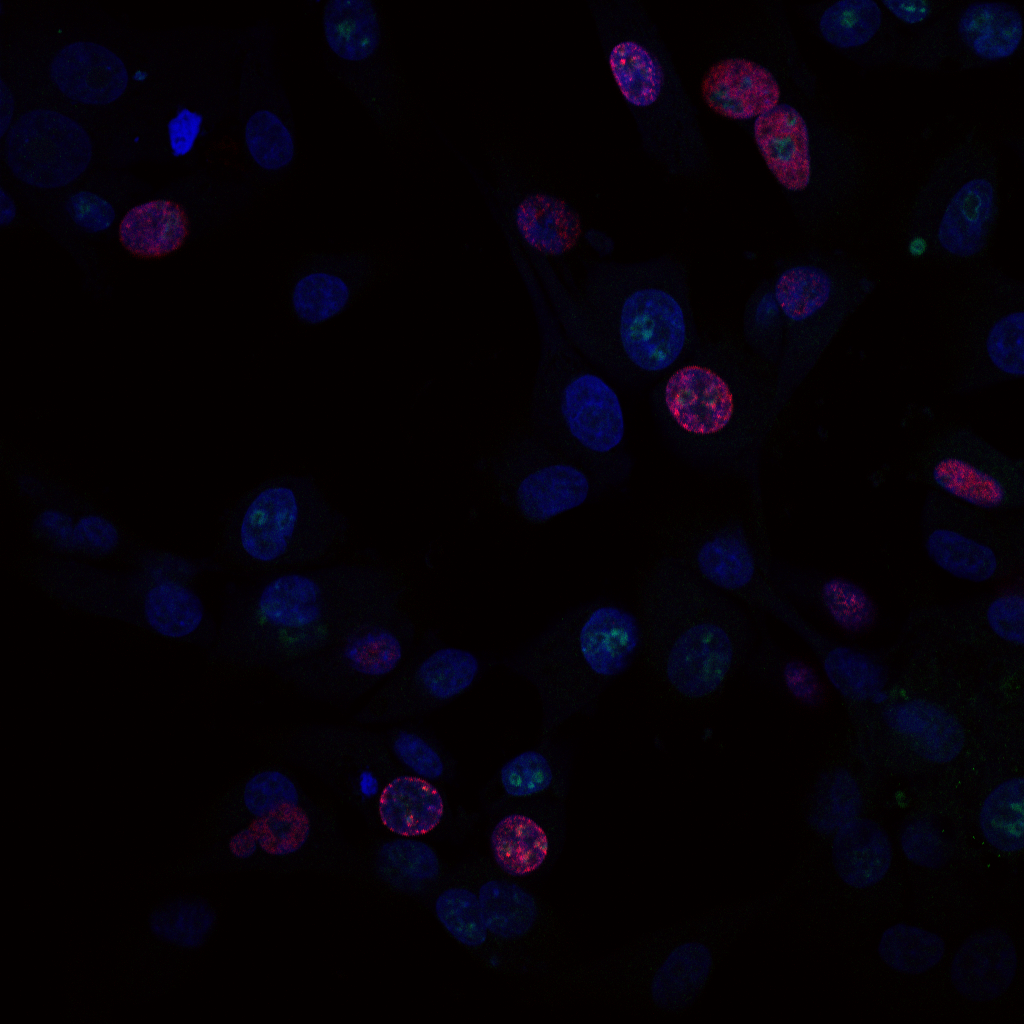

Supplement: Supplementary file 8 — Source Data Fig. 3 [file 44319_2023_17_MOESM8_ESM.zip › Fig3_source data/Fig3A_source data.tif]

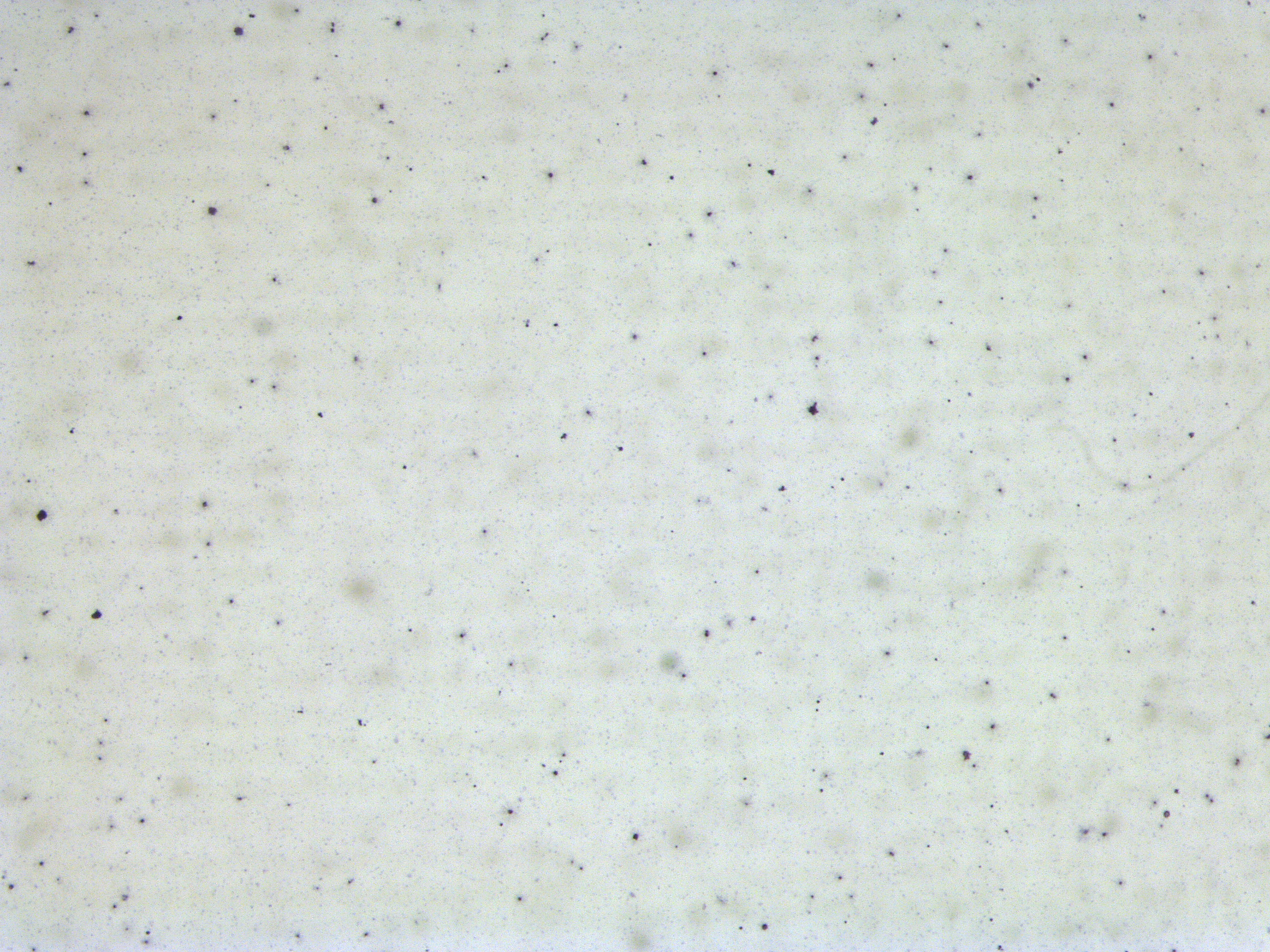

Supplement: Supplementary file 8 — Source Data Fig. 3 [file 44319_2023_17_MOESM8_ESM.zip › Fig3_source data/Fig3Q_left panel_source data.tif]

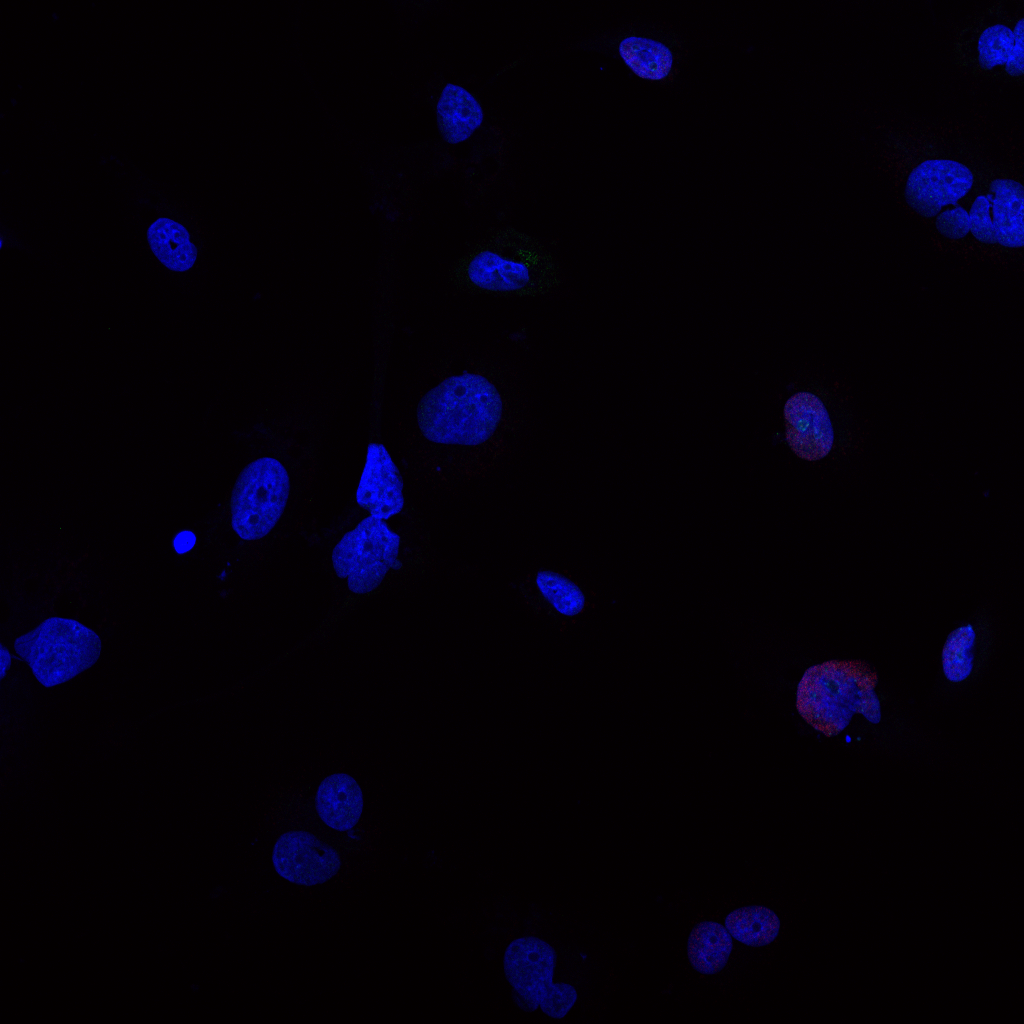

Supplement: Supplementary file 8 — Source Data Fig. 3 [file 44319_2023_17_MOESM8_ESM.zip › Fig3_source data/Fig3D_source data.tif]

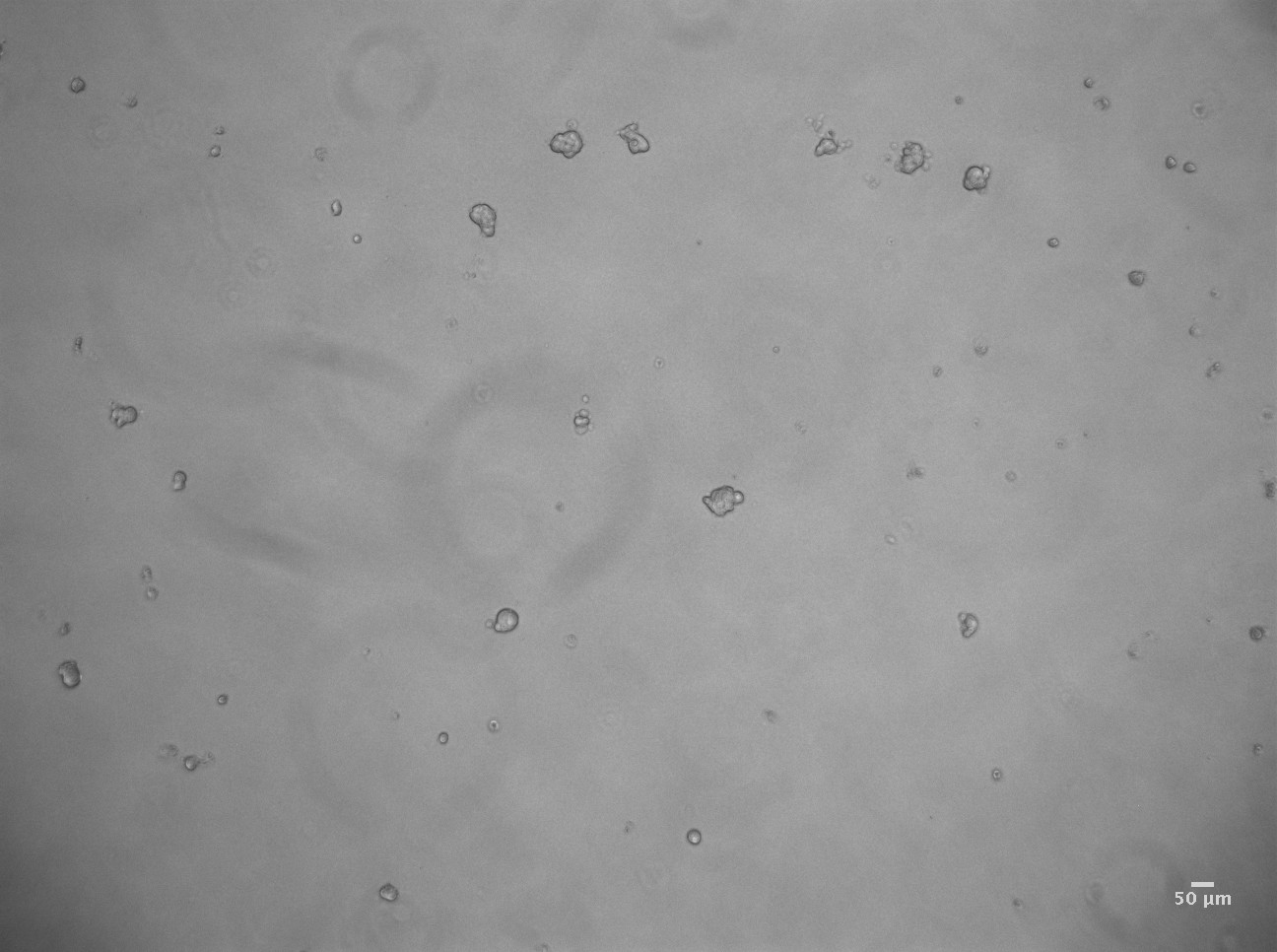

Supplement: Supplementary file 8 — Source Data Fig. 3 [file 44319_2023_17_MOESM8_ESM.zip › Fig3_source data/Fig3Q_right panel_source data.tif]

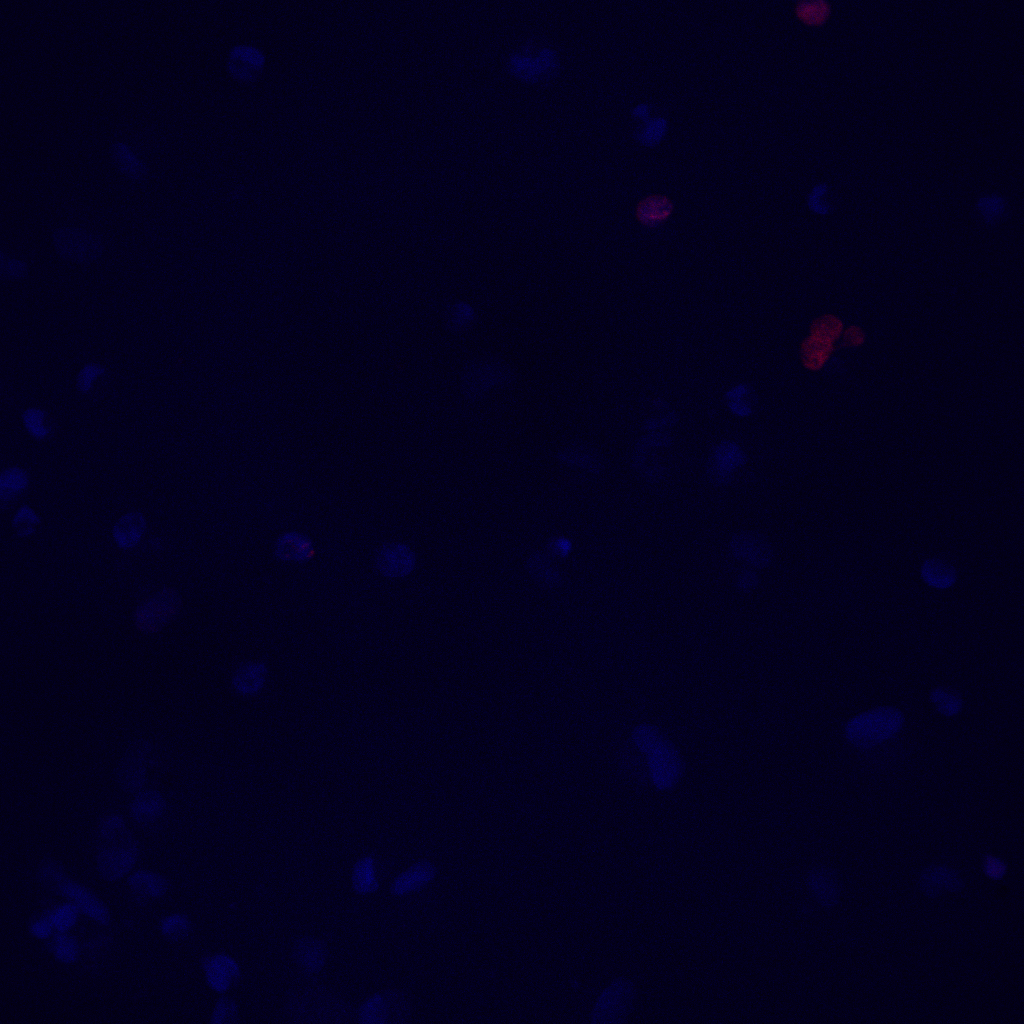

Supplement: Supplementary file 8 — Source Data Fig. 3 [file 44319_2023_17_MOESM8_ESM.zip › Fig3_source data/Fig3K_source data.tif]

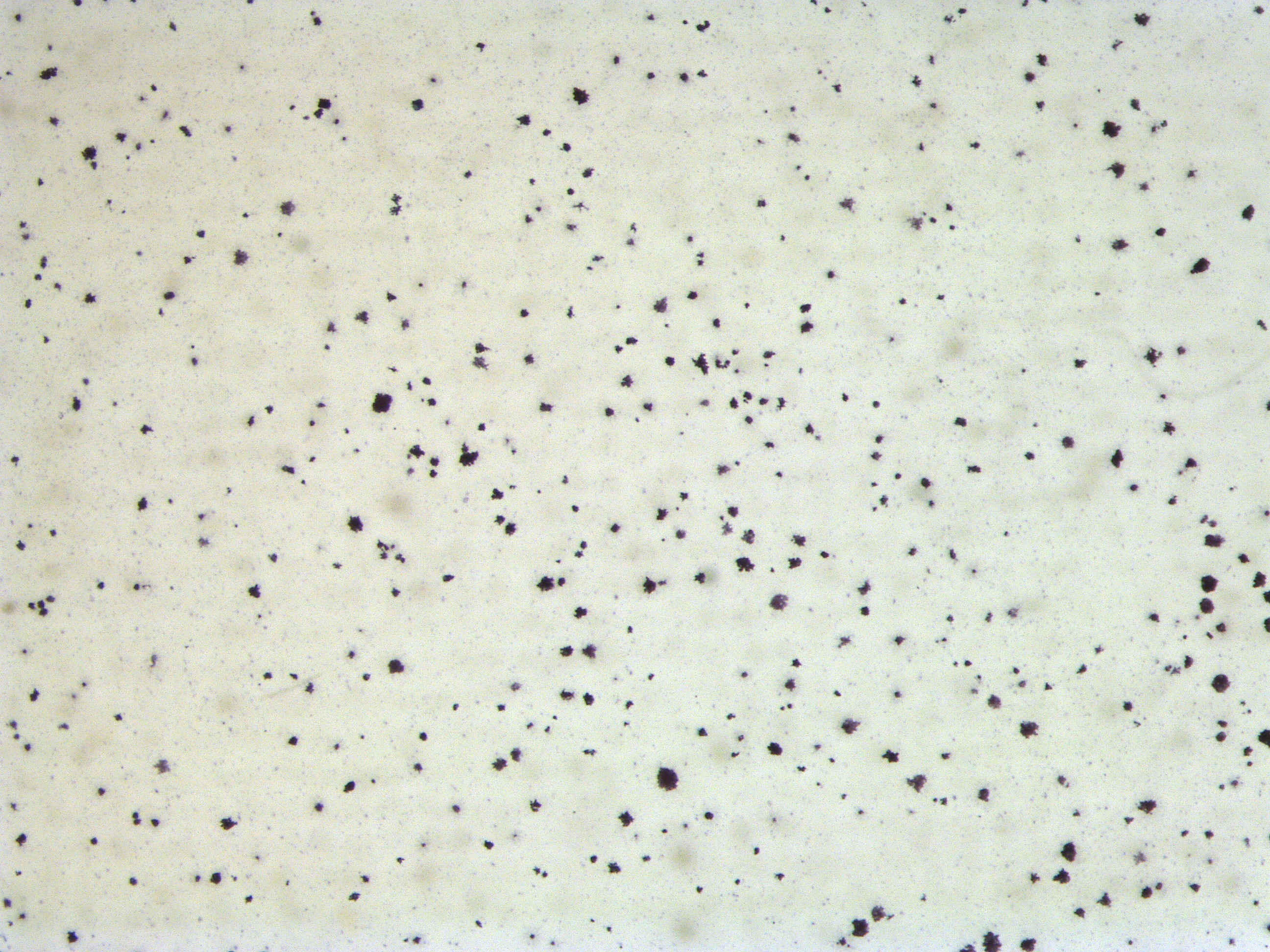

Supplement: Supplementary file 8 — Source Data Fig. 3 [file 44319_2023_17_MOESM8_ESM.zip › Fig3_source data/Fig3M_left panel_source data.tif]

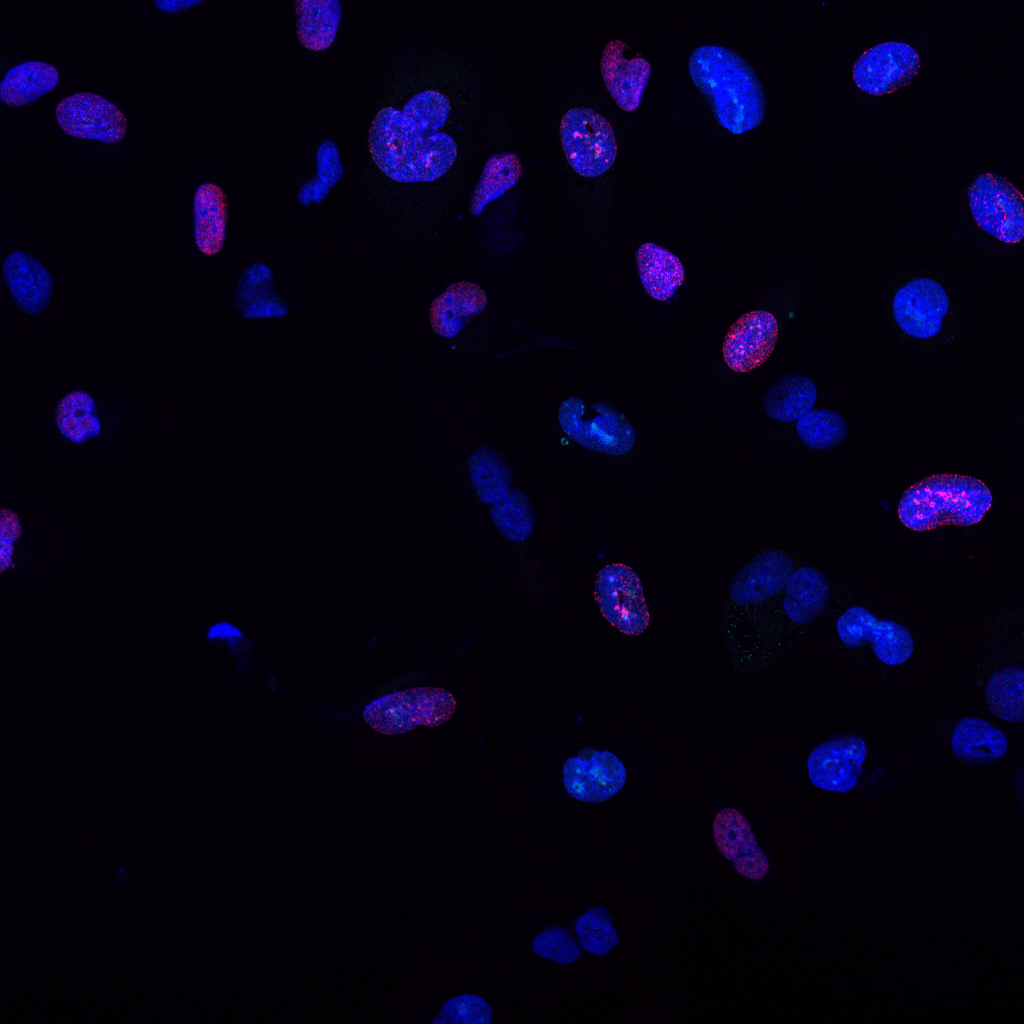

Supplement: Supplementary file 8 — Source Data Fig. 3 [file 44319_2023_17_MOESM8_ESM.zip › Fig3_source data/Fig3C_source data.tif]

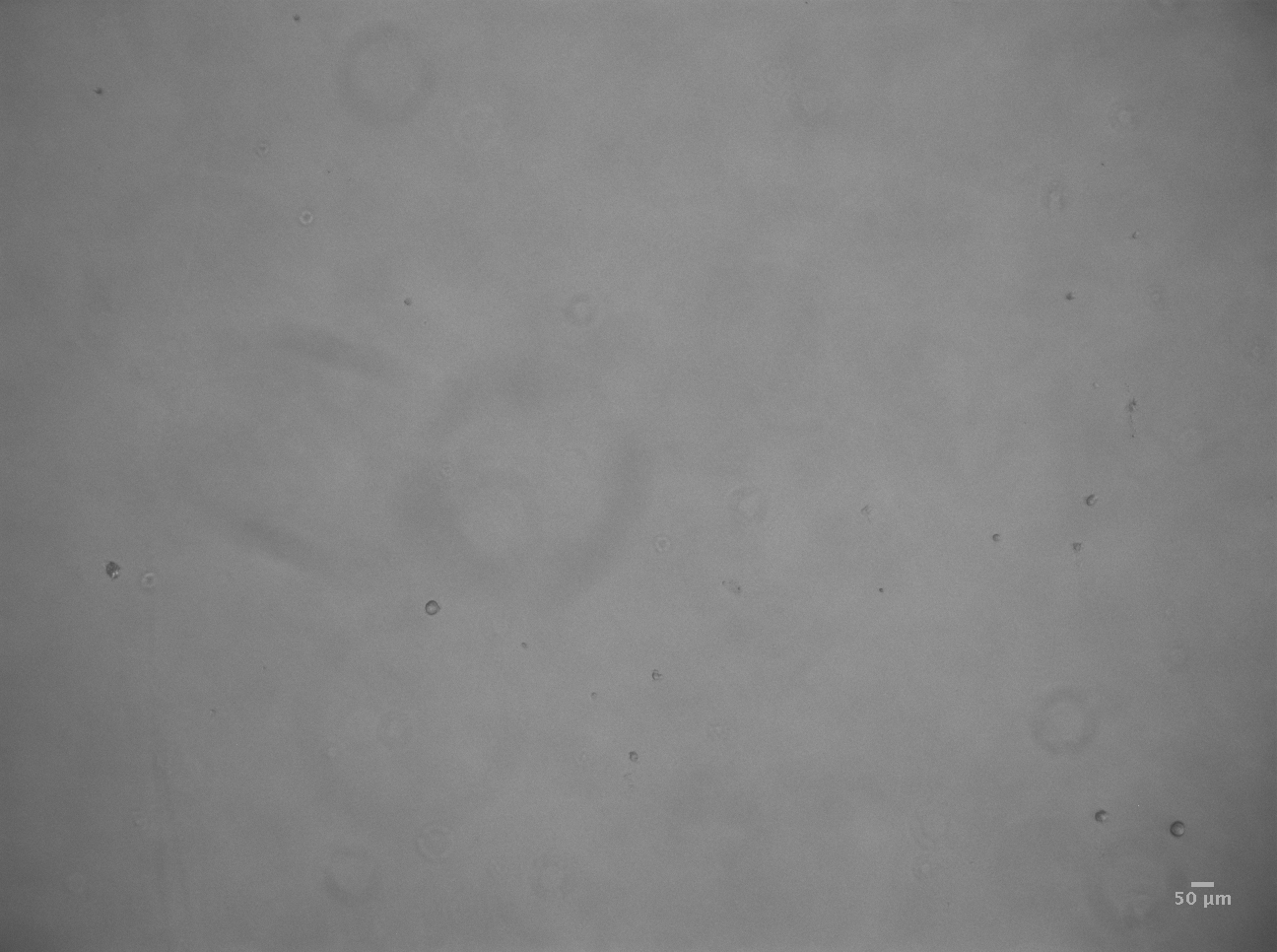

Supplement: Supplementary file 8 — Source Data Fig. 3 [file 44319_2023_17_MOESM8_ESM.zip › Fig3_source data/Fig3P_right panel_source data.tif]

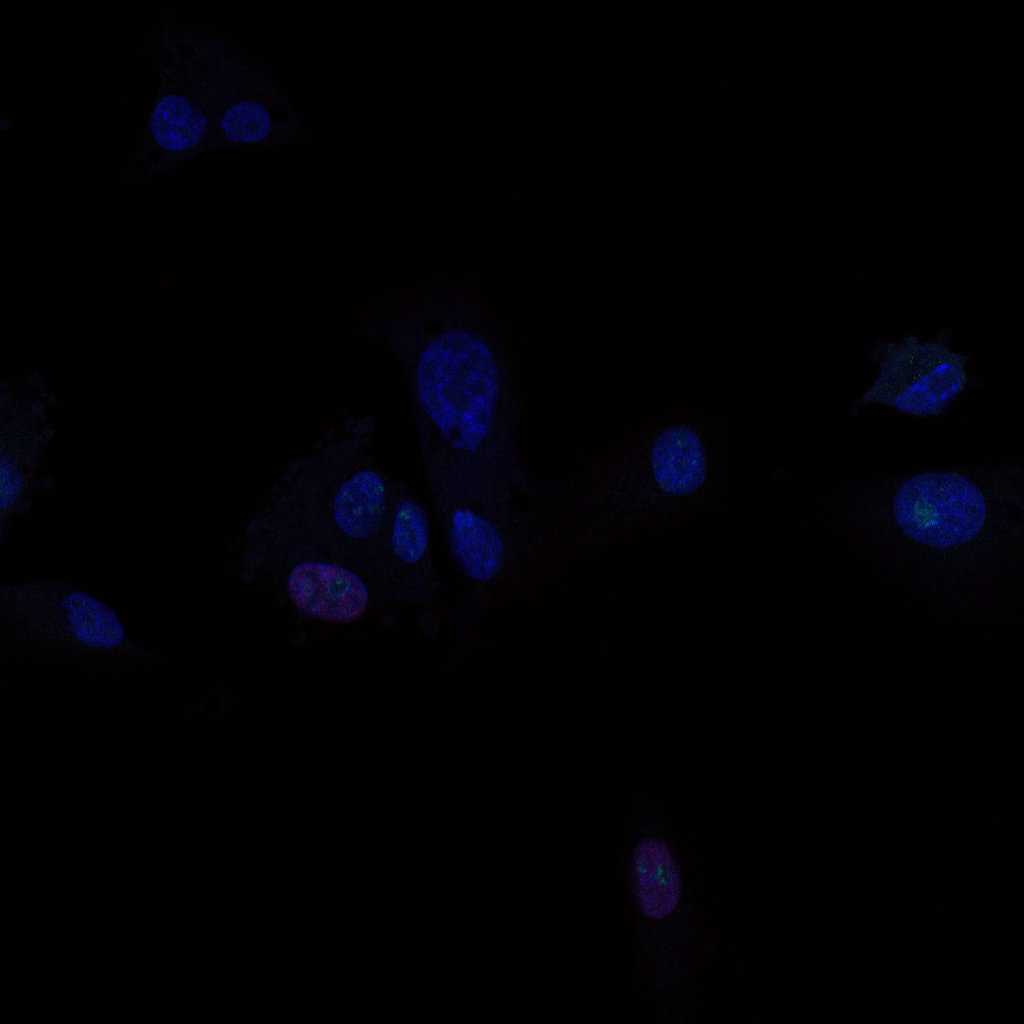

Supplement: Supplementary file 8 — Source Data Fig. 3 [file 44319_2023_17_MOESM8_ESM.zip › Fig3_source data/Fig3B_source data.tif]

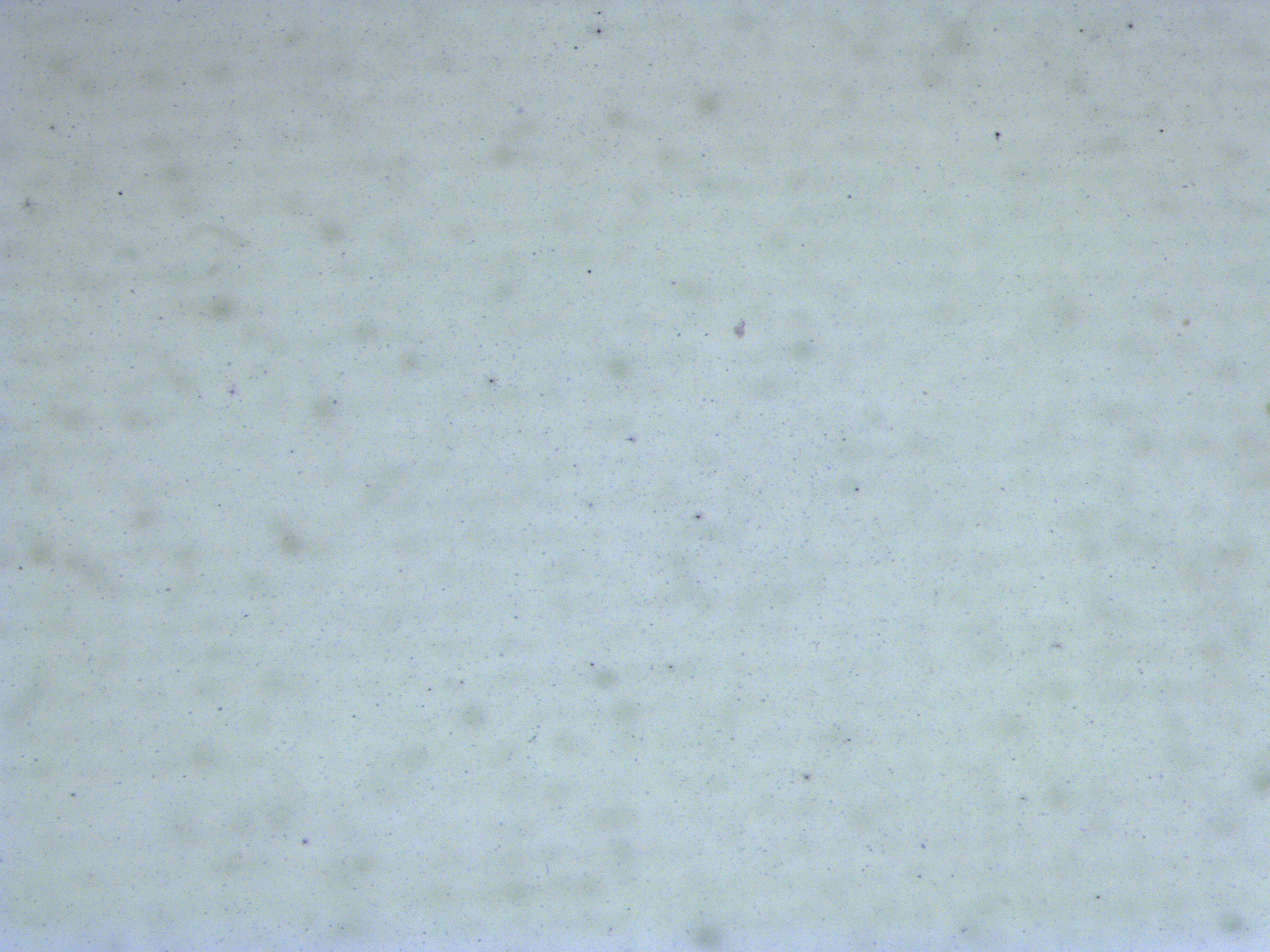

Supplement: Supplementary file 8 — Source Data Fig. 3 [file 44319_2023_17_MOESM8_ESM.zip › Fig3_source data/Fig3P_left panel_source data.tif]

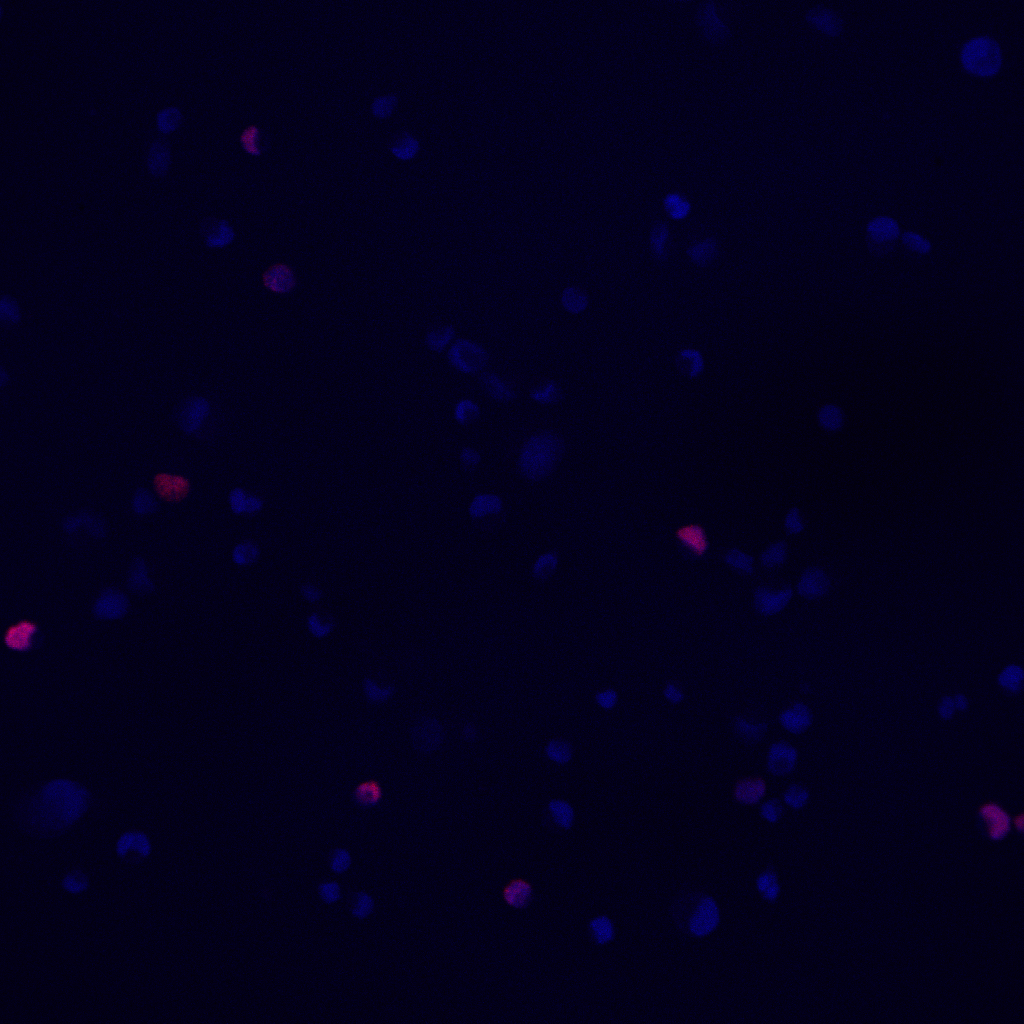

Supplement: Supplementary file 8 — Source Data Fig. 3 [file 44319_2023_17_MOESM8_ESM.zip › Fig3_source data/Fig3J_source data.tif]

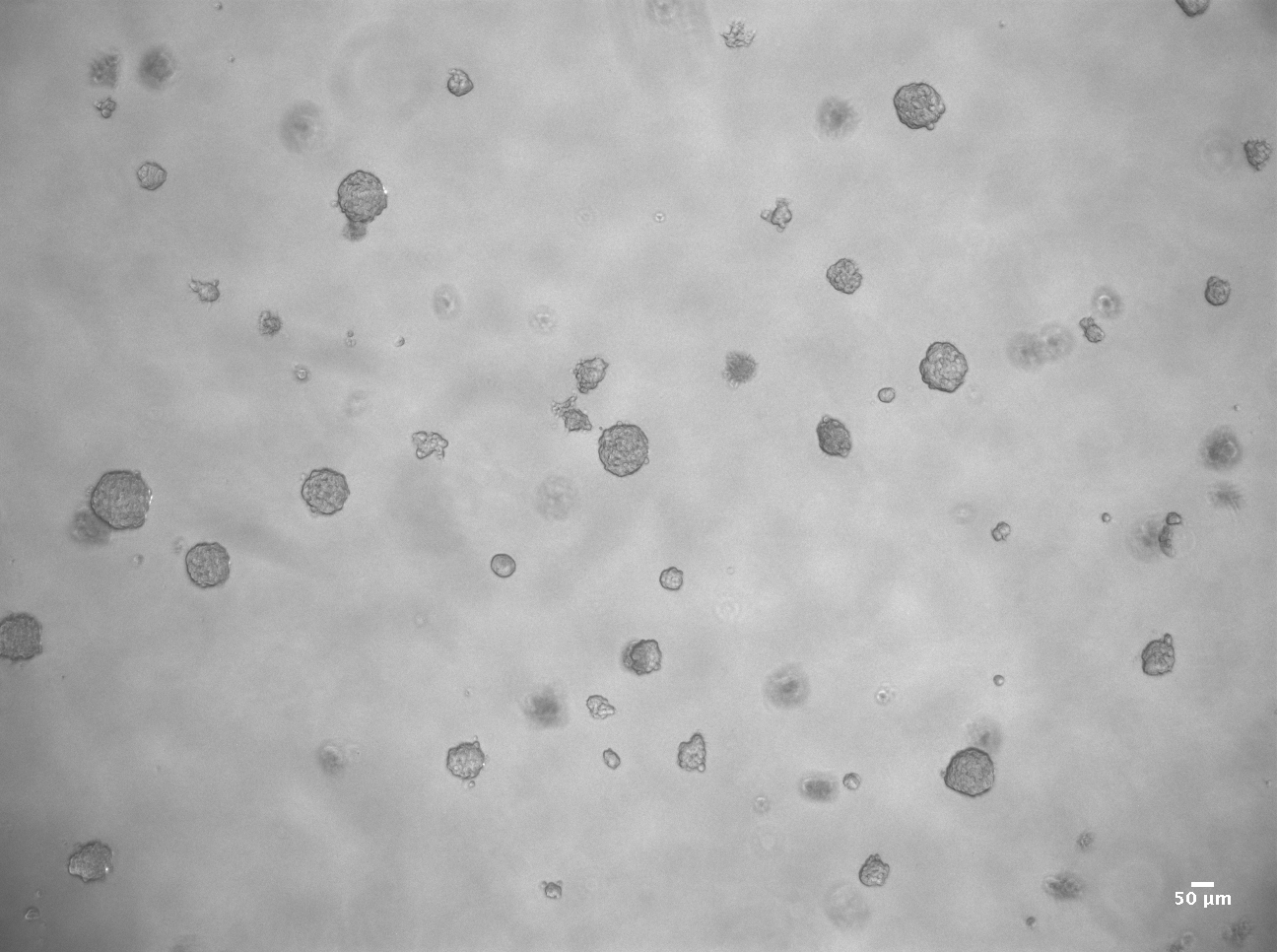

Supplement: Supplementary file 8 — Source Data Fig. 3 [file 44319_2023_17_MOESM8_ESM.zip › Fig3_source data/Fig3M_right panel_source data.tif]

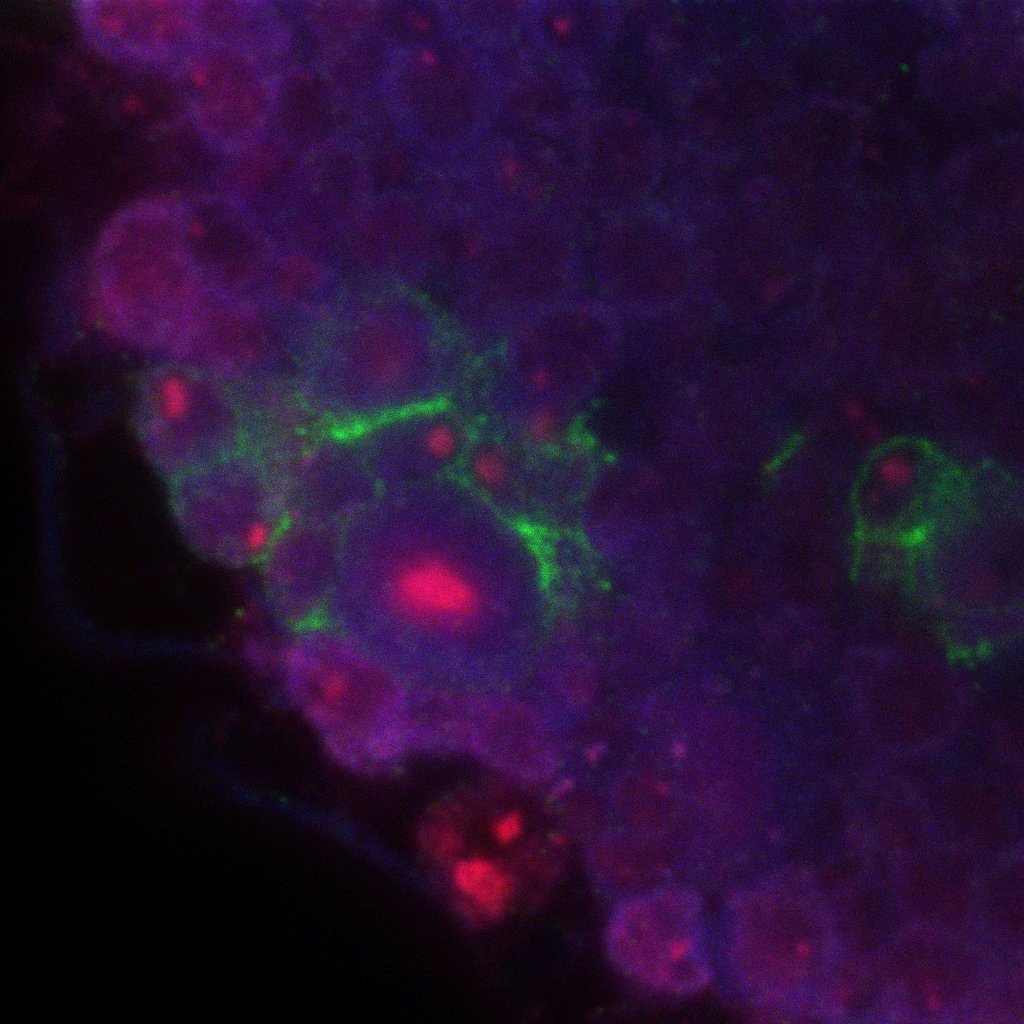

Supplement: Supplementary file 9 — Source Data Fig. 4 [file 44319_2023_17_MOESM9_ESM.zip › Fig4_source data/Fig4D_source data.jpg]

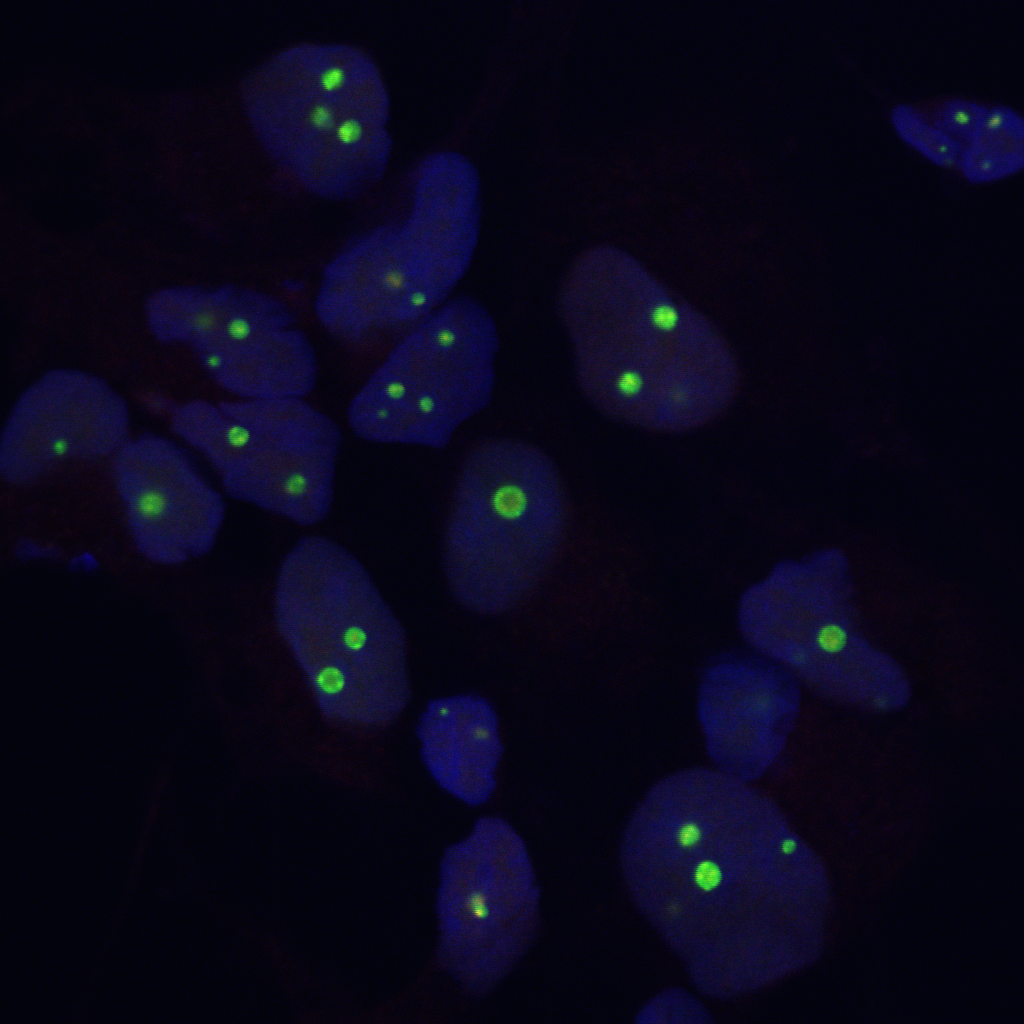

Supplement: Supplementary file 9 — Source Data Fig. 4 [file 44319_2023_17_MOESM9_ESM.zip › Fig4_source data/Fig4J_source data.tif]

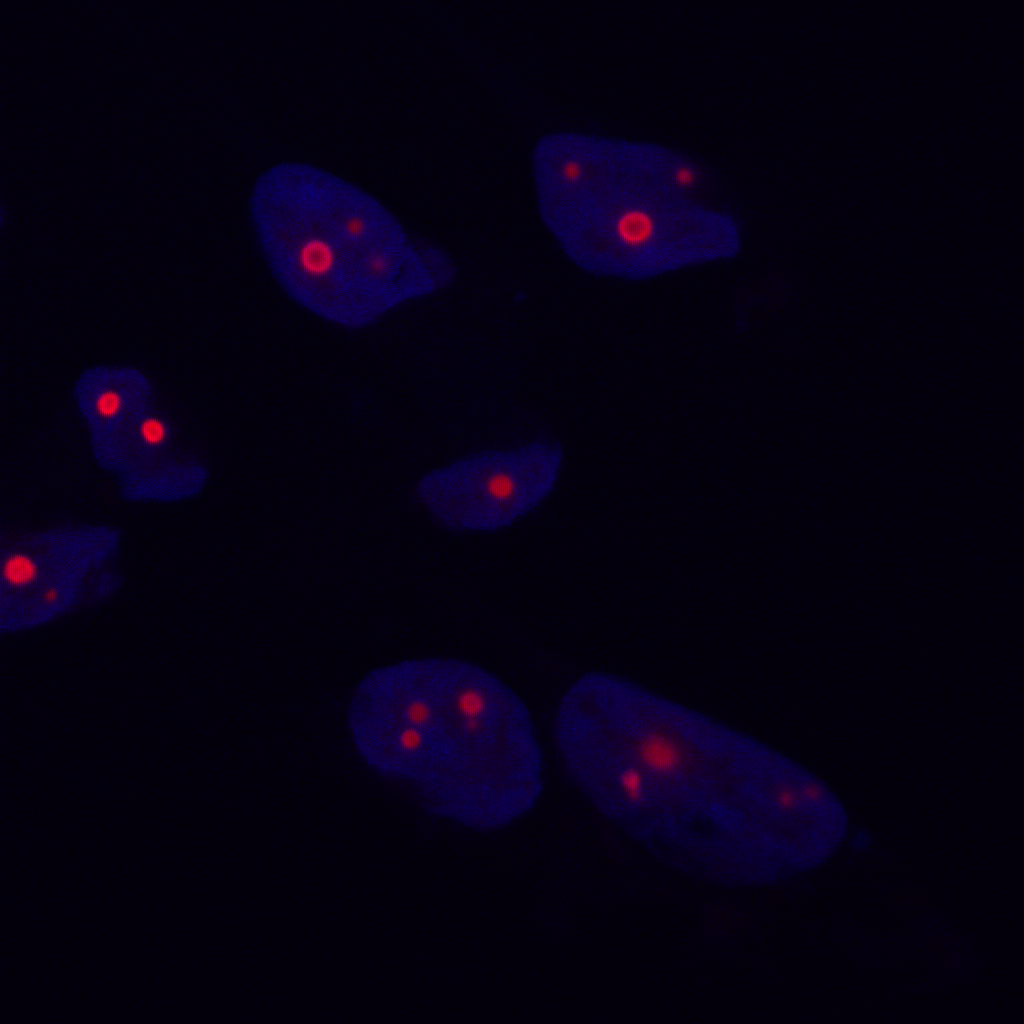

Supplement: Supplementary file 9 — Source Data Fig. 4 [file 44319_2023_17_MOESM9_ESM.zip › Fig4_source data/Fig4M_source data.tif]

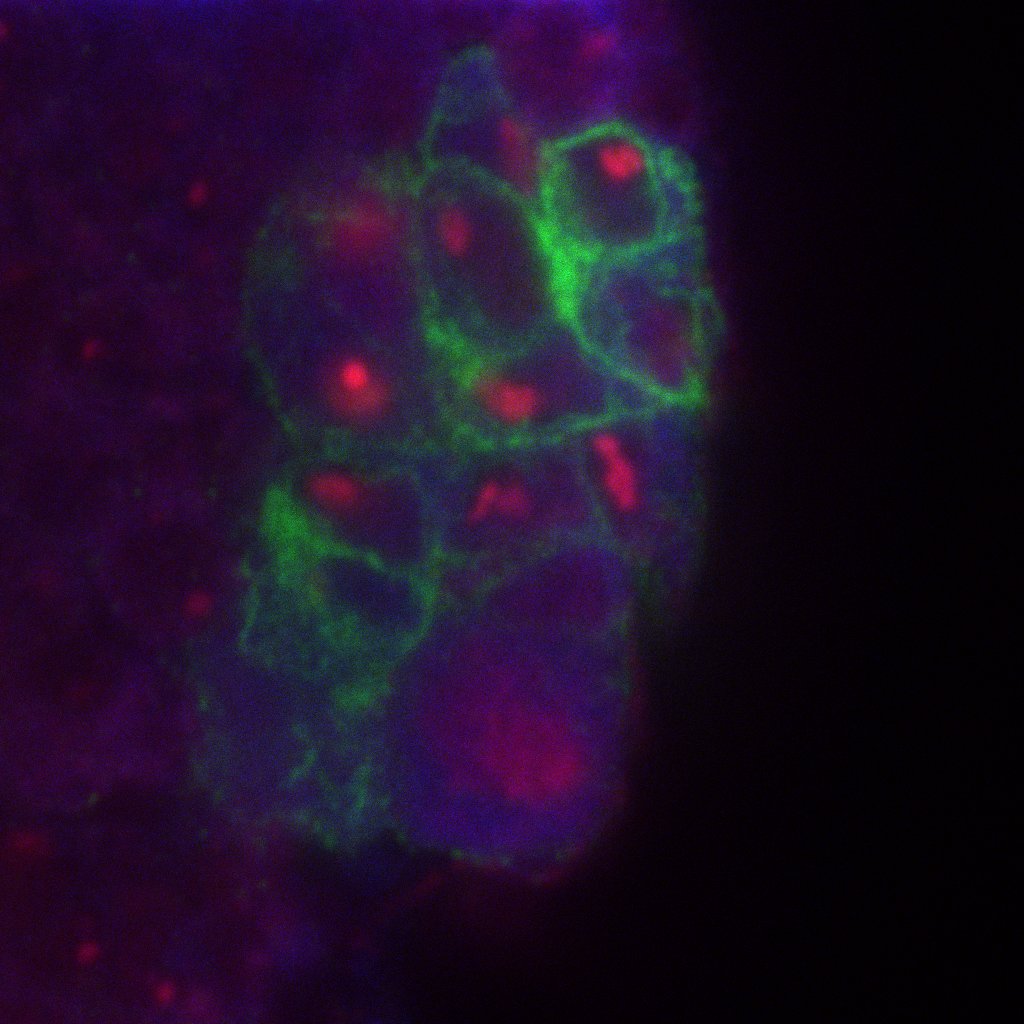

Supplement: Supplementary file 9 — Source Data Fig. 4 [file 44319_2023_17_MOESM9_ESM.zip › Fig4_source data/Fig4C_source data.jpg]

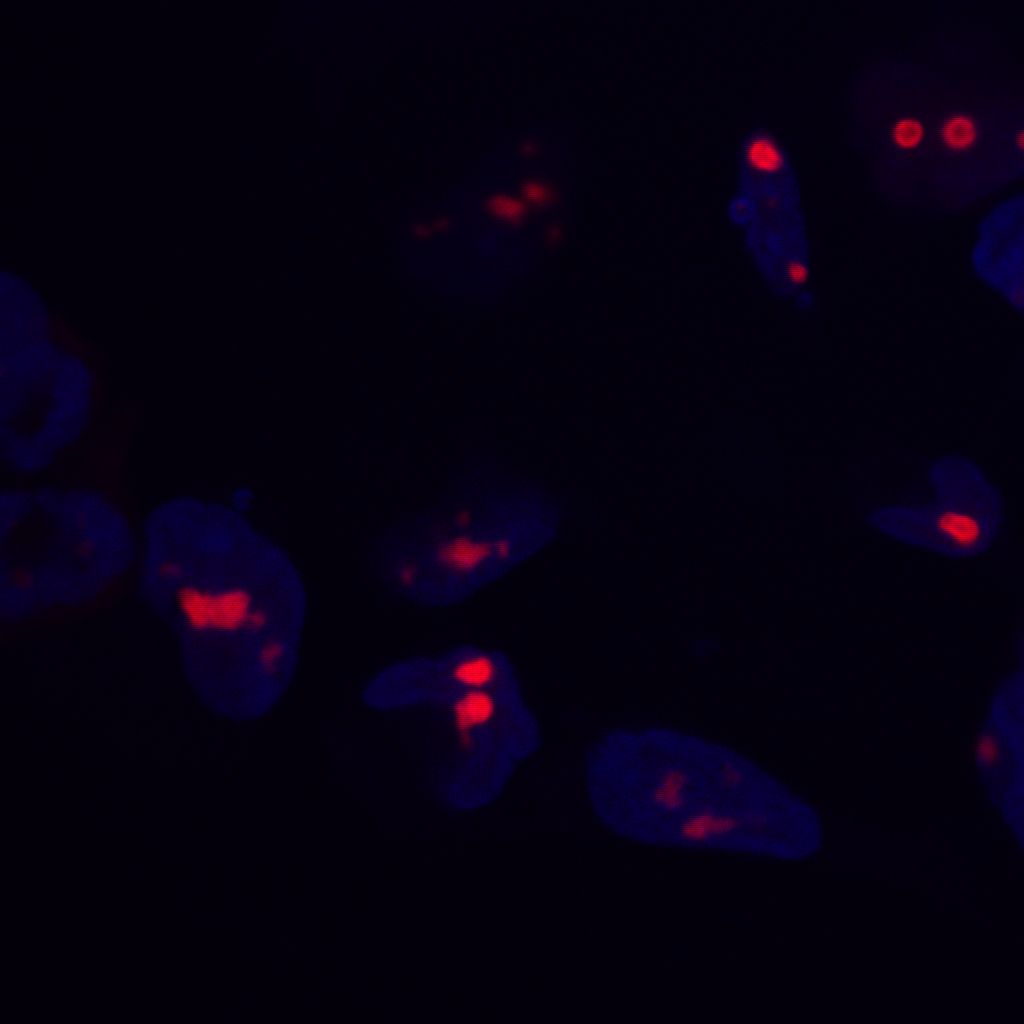

Supplement: Supplementary file 9 — Source Data Fig. 4 [file 44319_2023_17_MOESM9_ESM.zip › Fig4_source data/Fig4L_source data.tif]

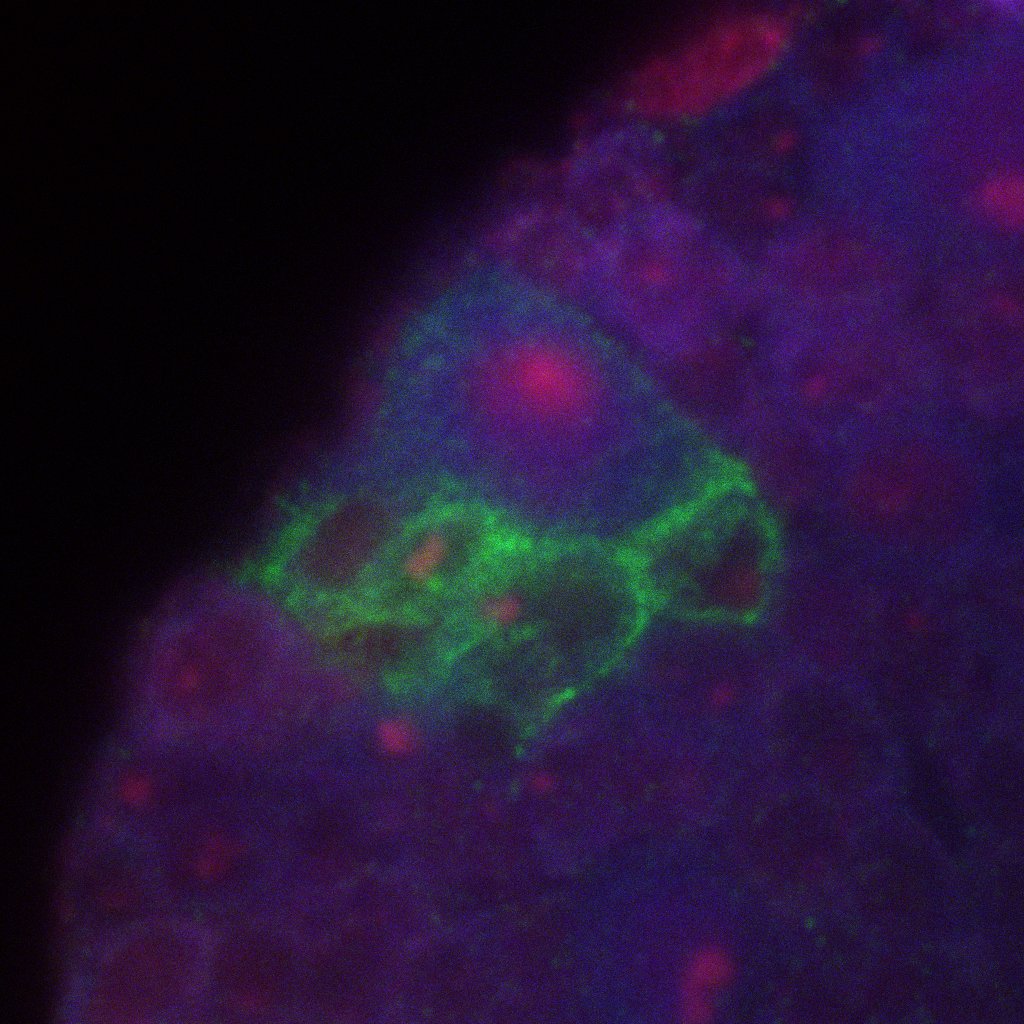

Supplement: Supplementary file 9 — Source Data Fig. 4 [file 44319_2023_17_MOESM9_ESM.zip › Fig4_source data/Fig4B_source data.jpg]

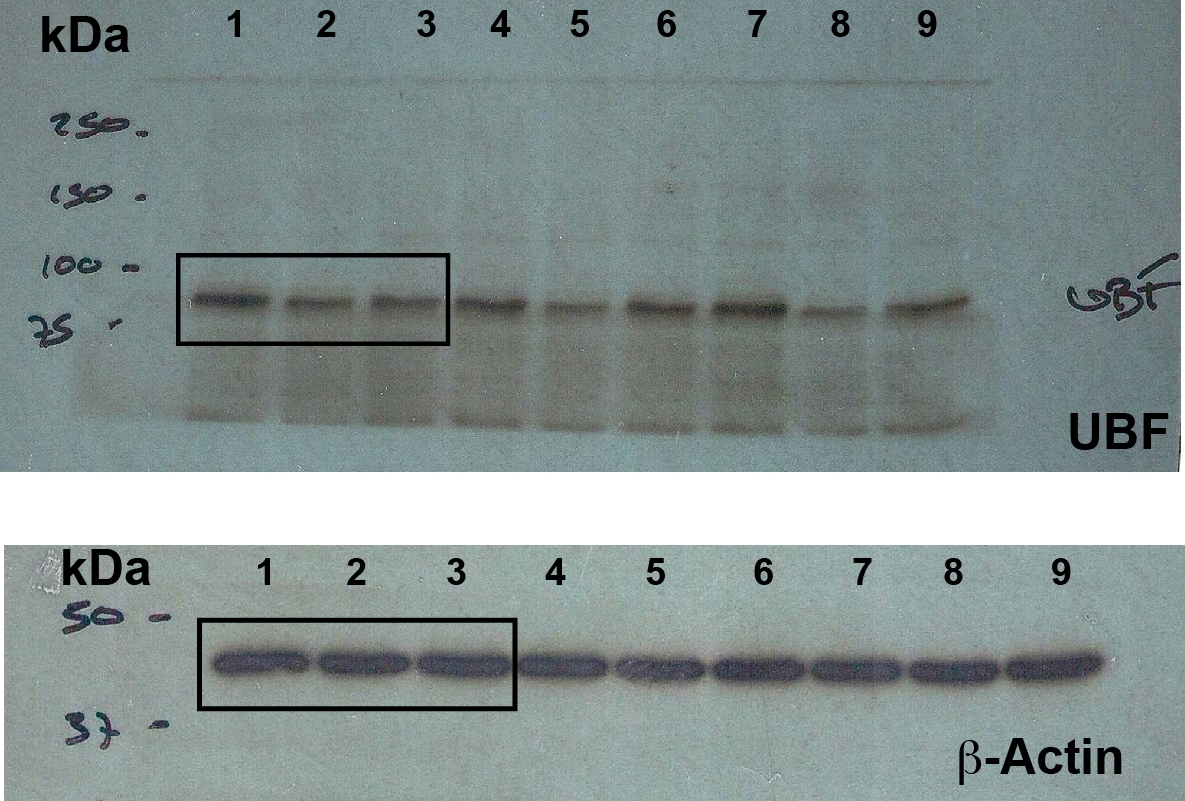

Supplement: Supplementary file 9 — Source Data Fig. 4 [file 44319_2023_17_MOESM9_ESM.zip › Fig4_source data/Fig4W_source data/Fig4W_UBF+loadingcontrol_source data.jpg]

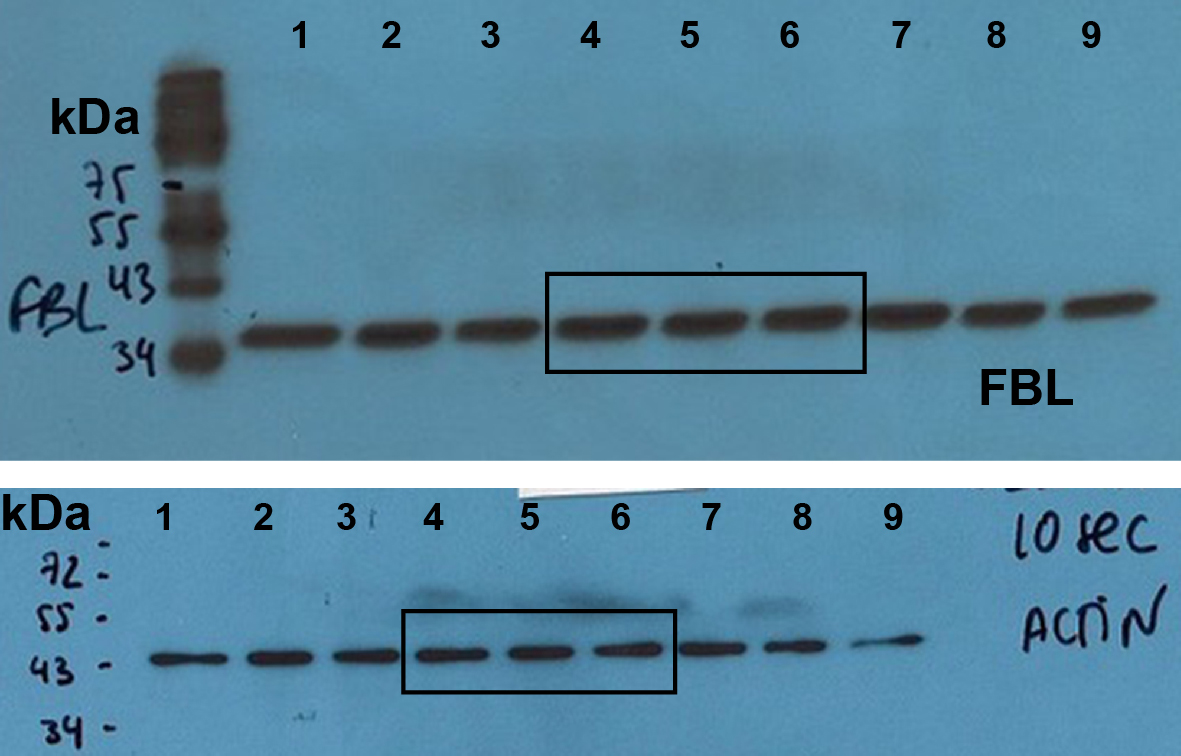

Supplement: Supplementary file 9 — Source Data Fig. 4 [file 44319_2023_17_MOESM9_ESM.zip › Fig4_source data/Fig4W_source data/Fig4W_FBL+loadingcontrol_source data.jpg]

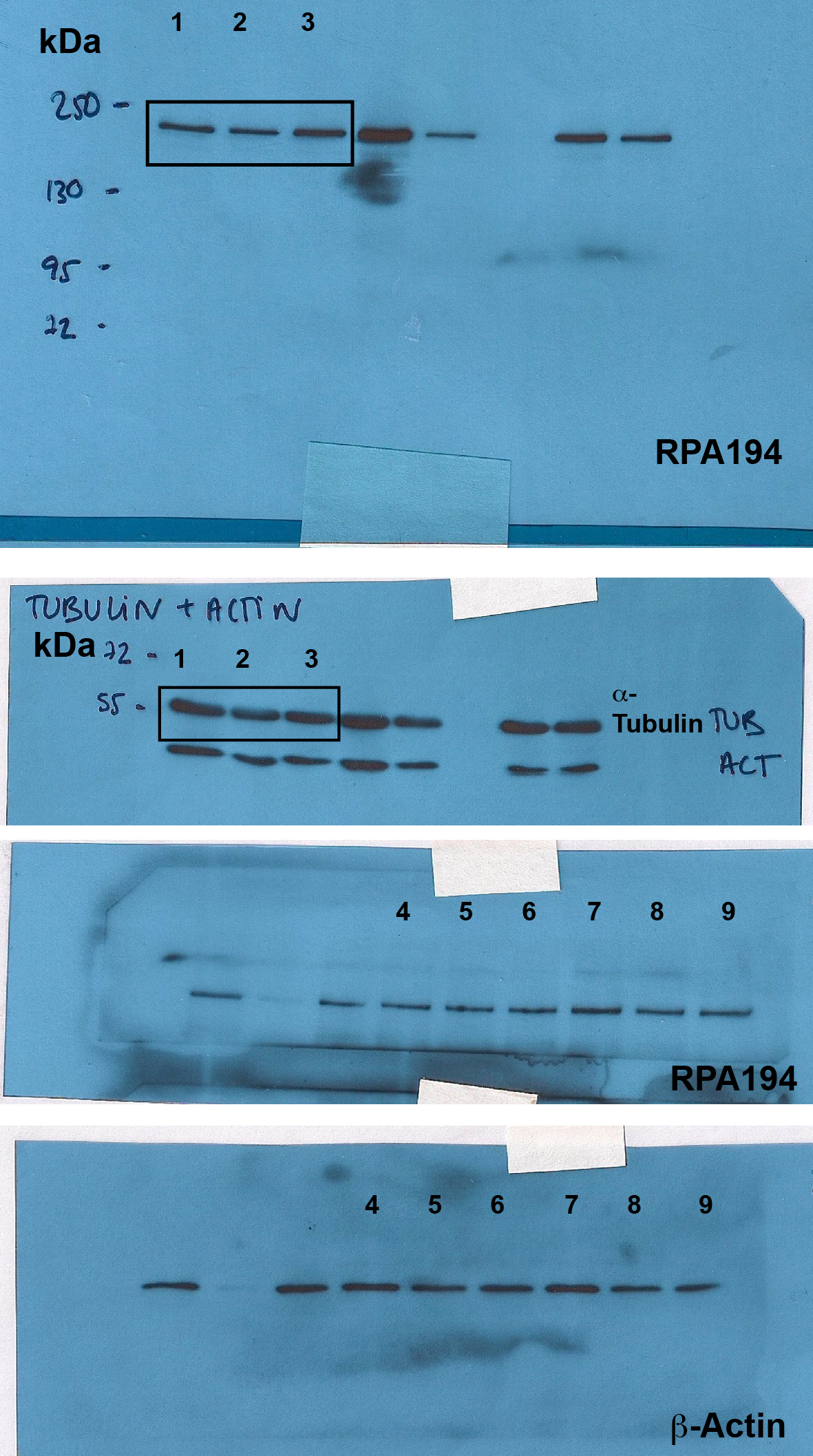

Supplement: Supplementary file 9 — Source Data Fig. 4 [file 44319_2023_17_MOESM9_ESM.zip › Fig4_source data/Fig4W_source data/Fig4W_RPA194+loadingcontrol_source data.jpg]

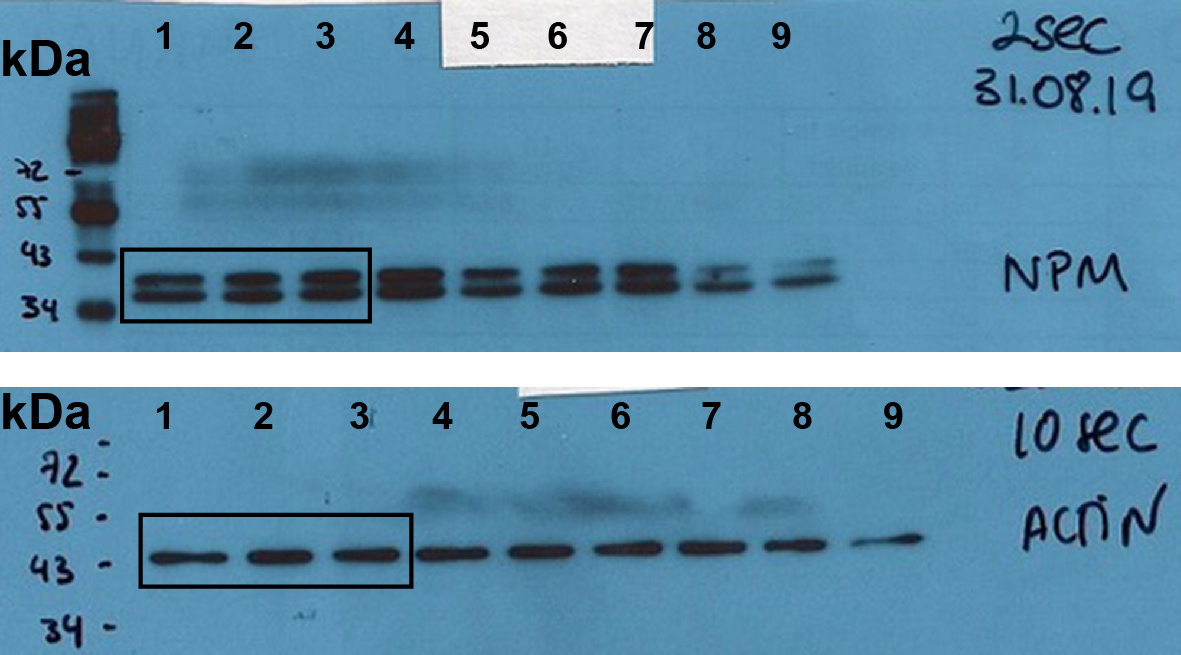

Supplement: Supplementary file 9 — Source Data Fig. 4 [file 44319_2023_17_MOESM9_ESM.zip › Fig4_source data/Fig4W_source data/Fig4W_NPM1+loadingcontrol_source data.jpg]

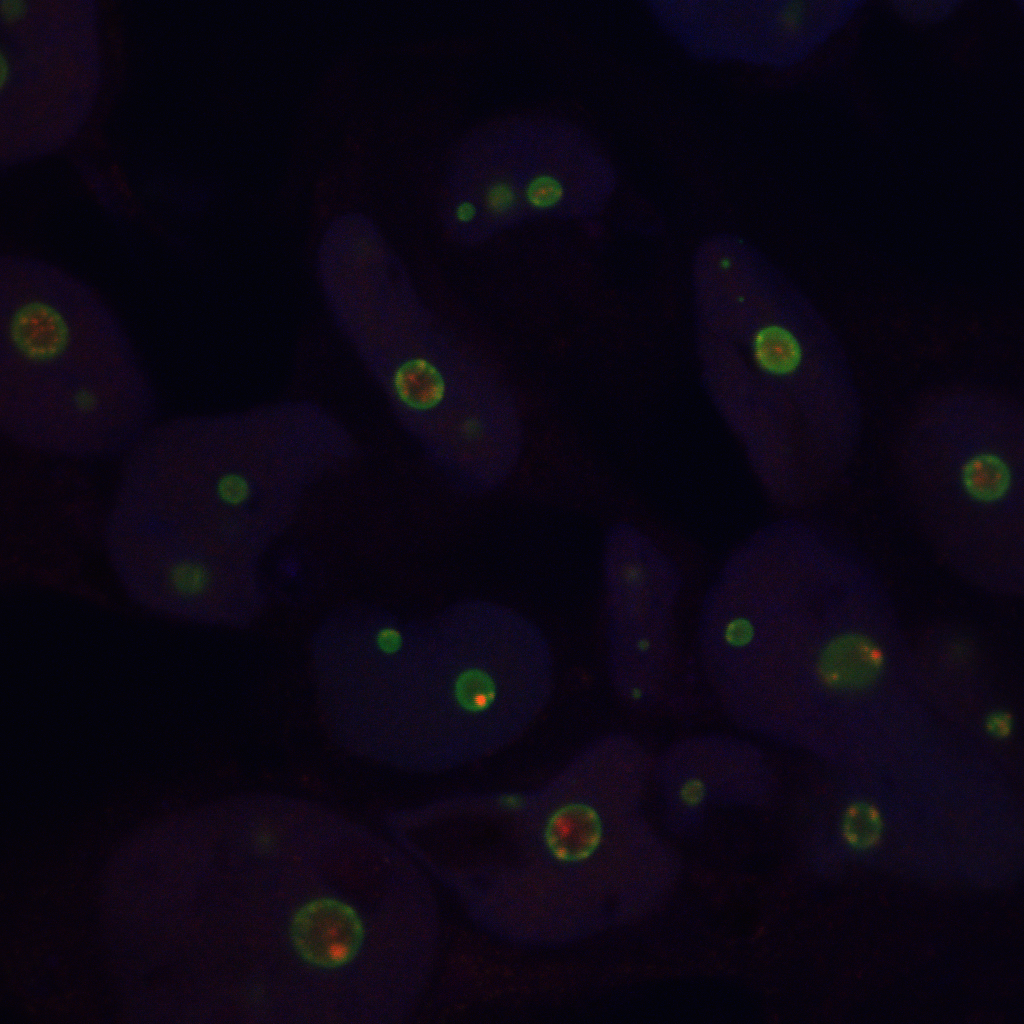

Supplement: Supplementary file 9 — Source Data Fig. 4 [file 44319_2023_17_MOESM9_ESM.zip › Fig4_source data/Fig4K_source data.tif]

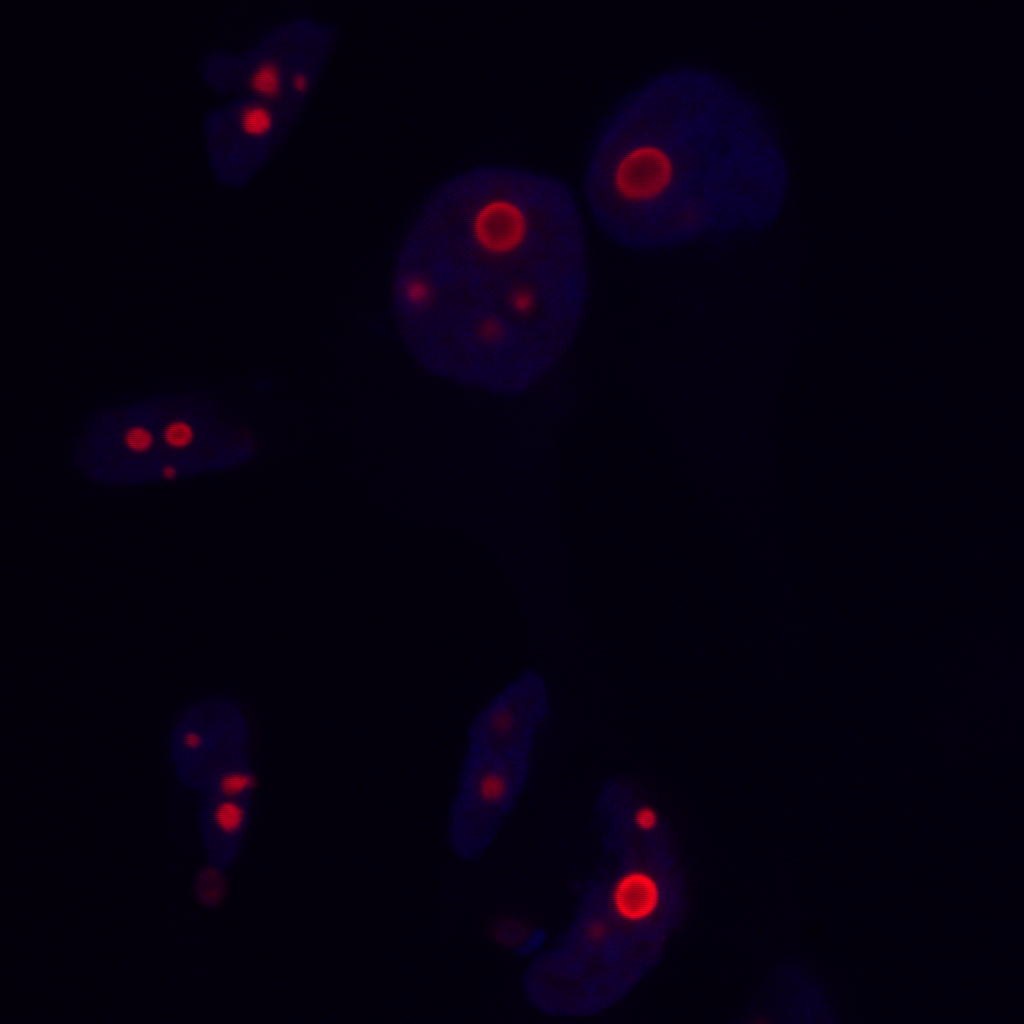

Supplement: Supplementary file 9 — Source Data Fig. 4 [file 44319_2023_17_MOESM9_ESM.zip › Fig4_source data/Fig4N_source data.tif]

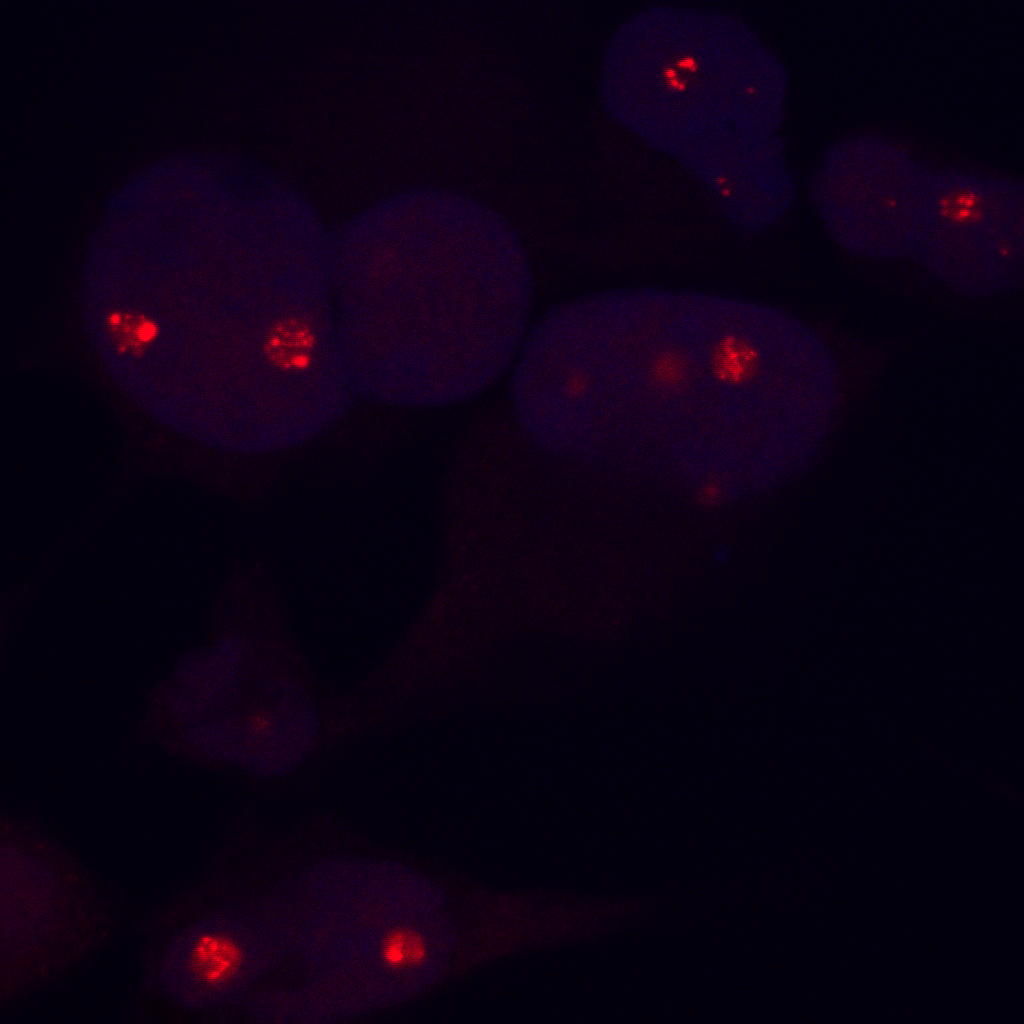

Supplement: Supplementary file 9 — Source Data Fig. 4 [file 44319_2023_17_MOESM9_ESM.zip › Fig4_source data/Fig4T_source data.tif]

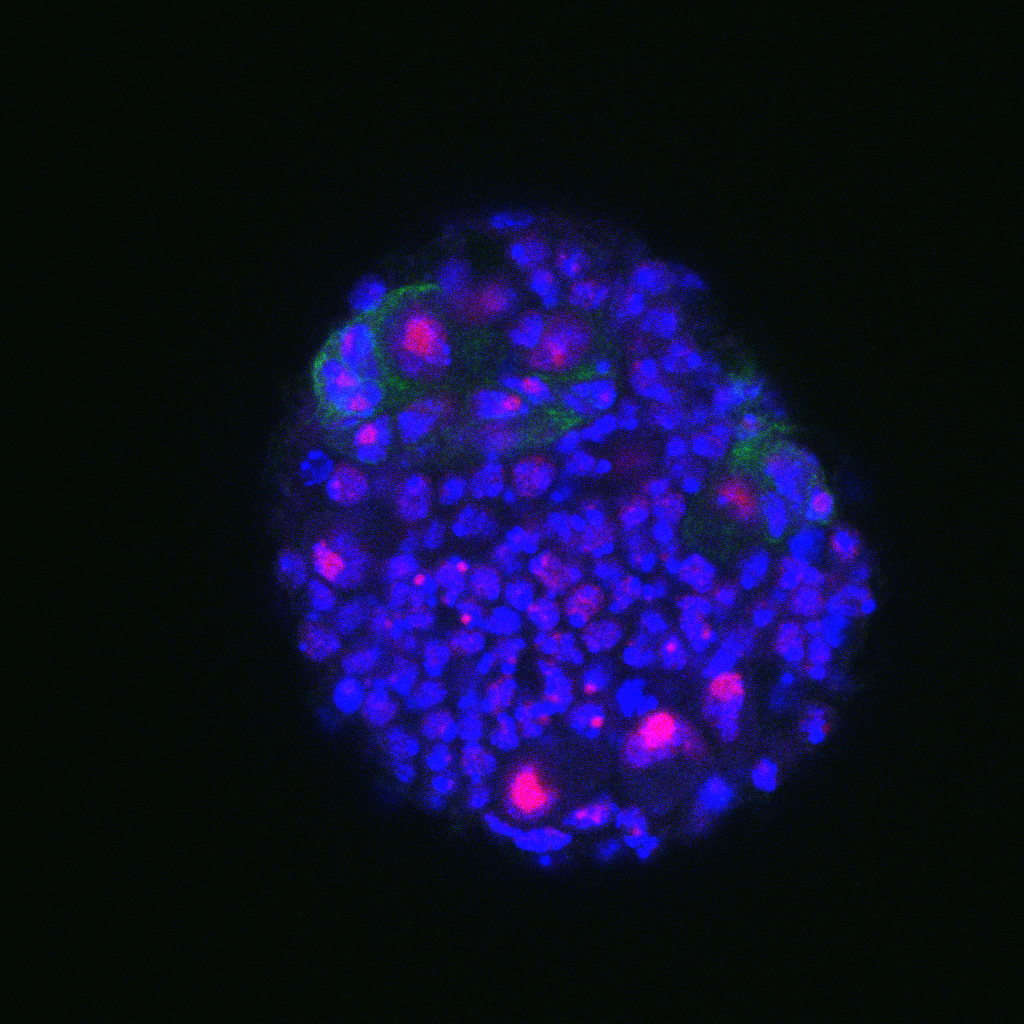

Supplement: Supplementary file 9 — Source Data Fig. 4 [file 44319_2023_17_MOESM9_ESM.zip › Fig4_source data/Fig4F_source data.tif]

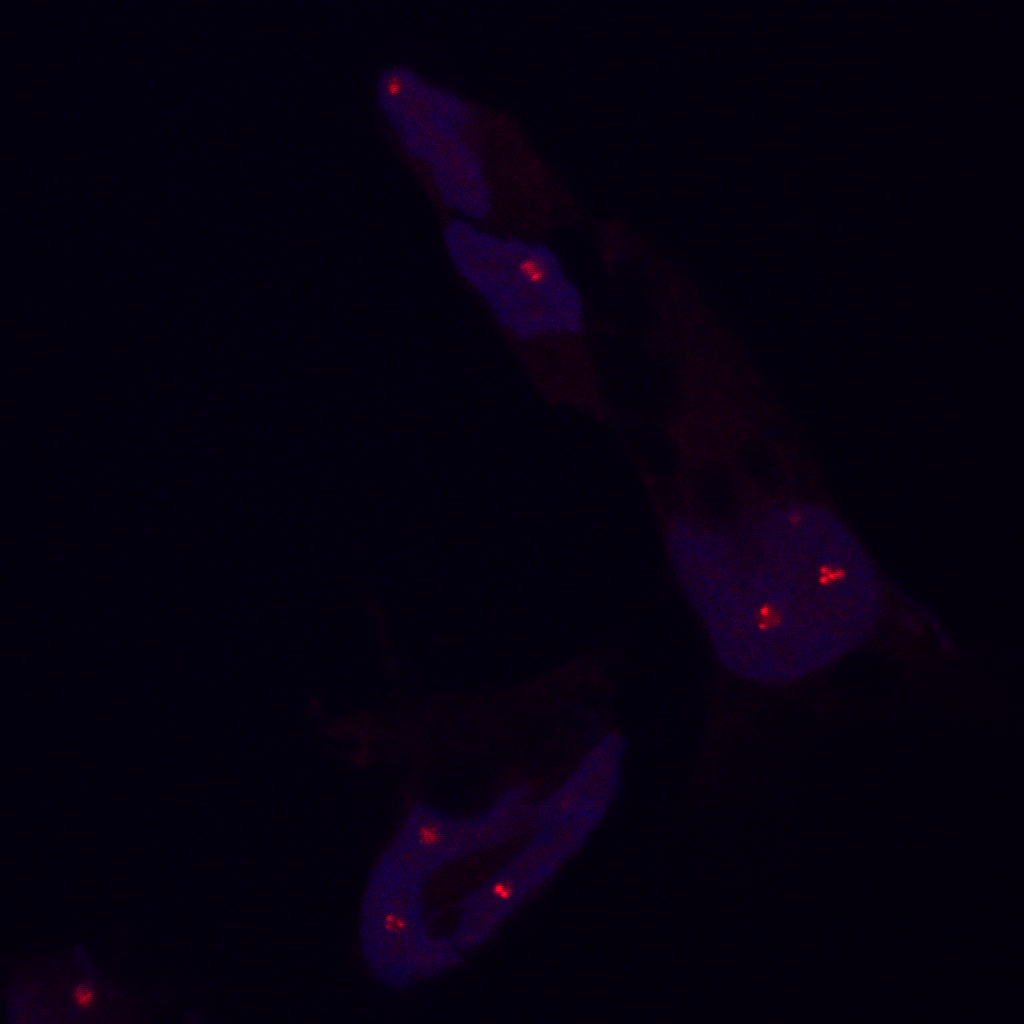

Supplement: Supplementary file 9 — Source Data Fig. 4 [file 44319_2023_17_MOESM9_ESM.zip › Fig4_source data/Fig4S_source data.tif]

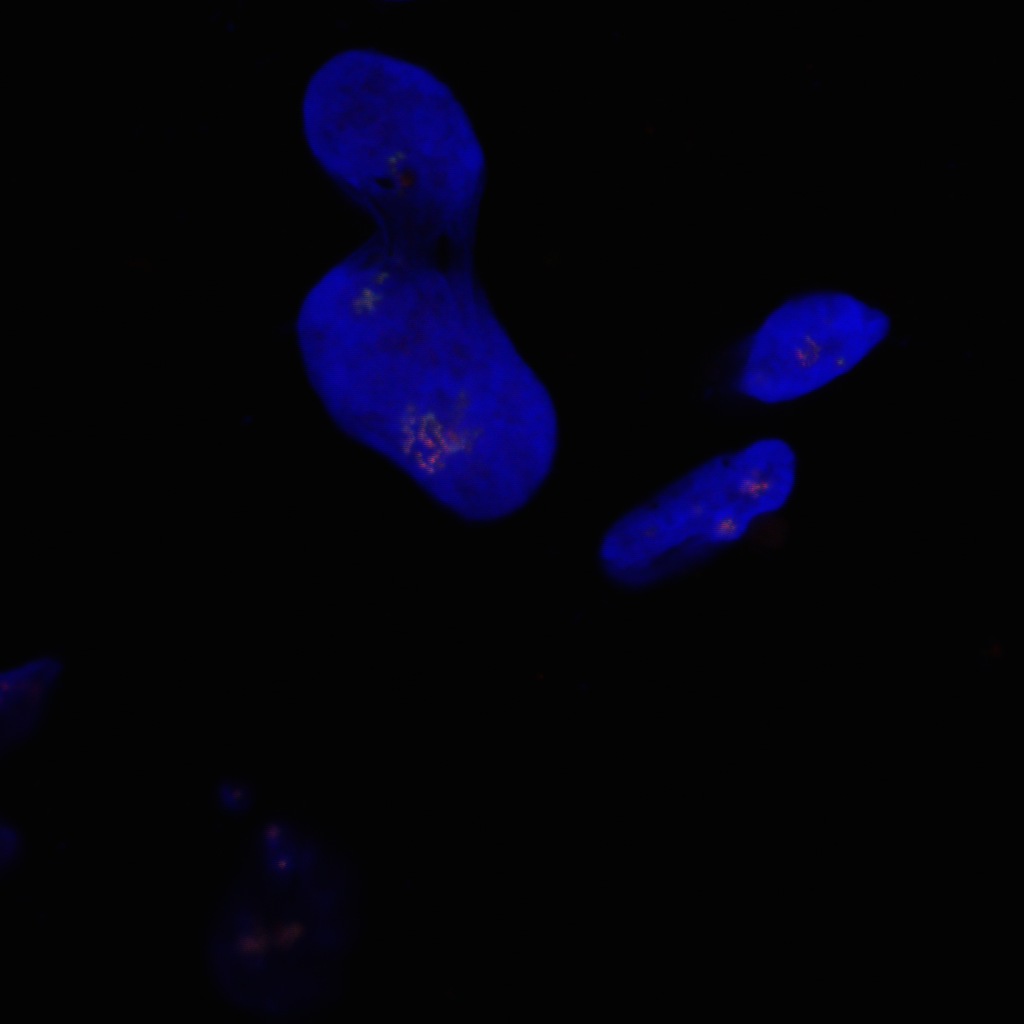

Supplement: Supplementary file 9 — Source Data Fig. 4 [file 44319_2023_17_MOESM9_ESM.zip › Fig4_source data/Fig4I_source data.tif]

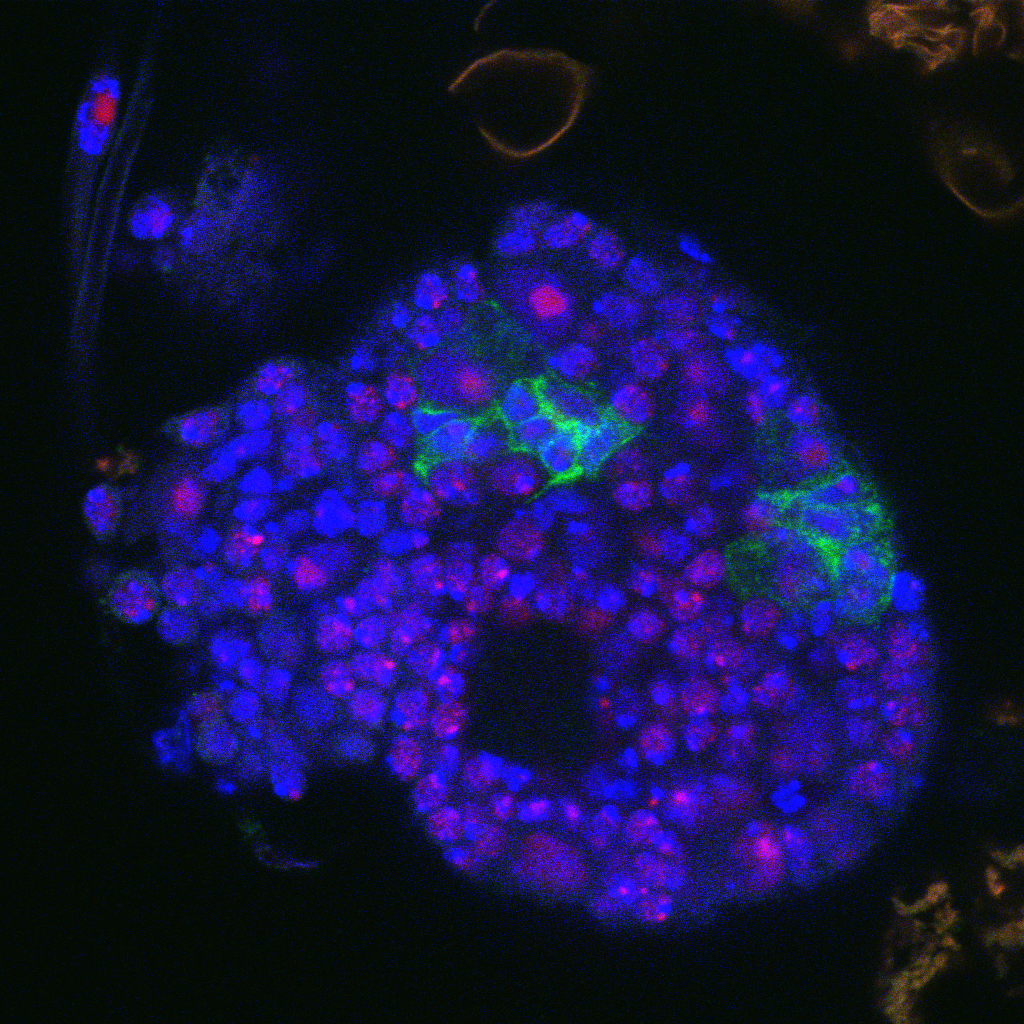

Supplement: Supplementary file 9 — Source Data Fig. 4 [file 44319_2023_17_MOESM9_ESM.zip › Fig4_source data/Fig4G_source data.tif]

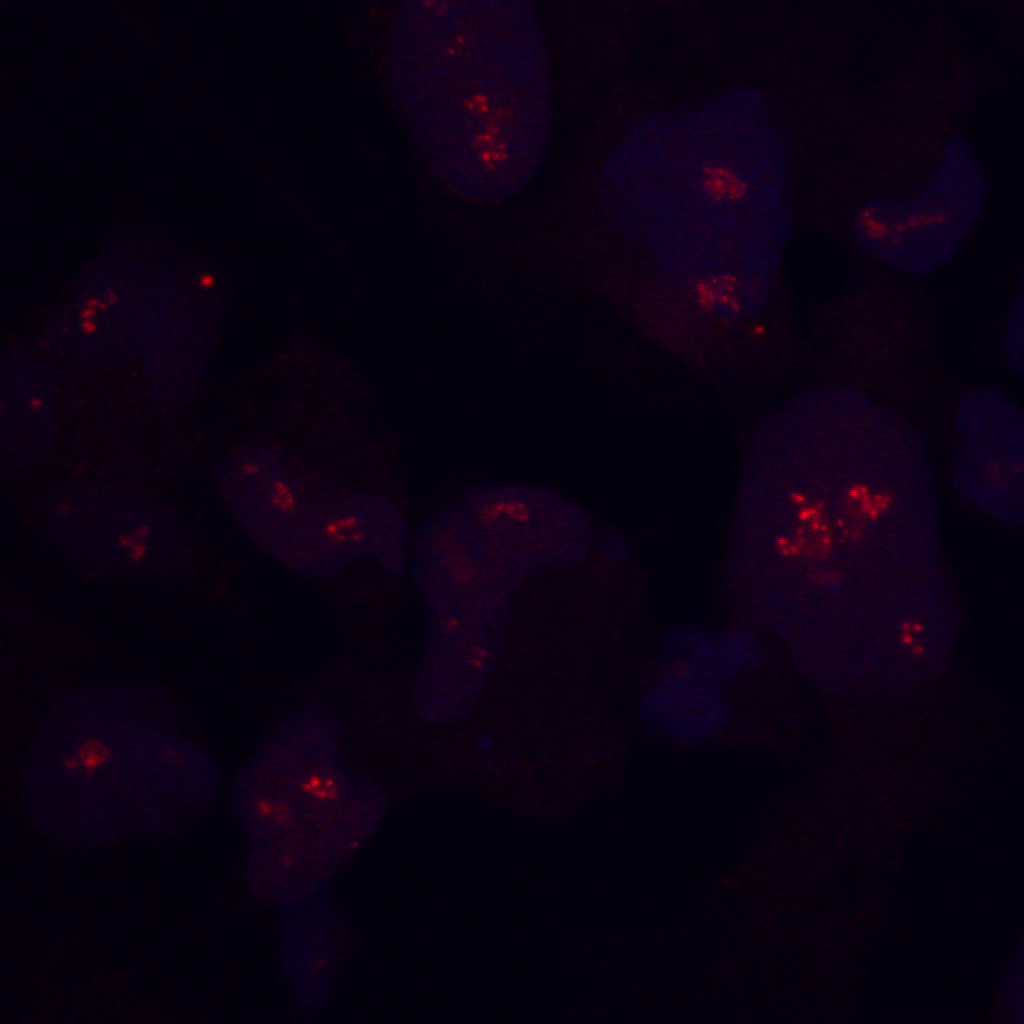

Supplement: Supplementary file 9 — Source Data Fig. 4 [file 44319_2023_17_MOESM9_ESM.zip › Fig4_source data/Fig4R_source data.tif]

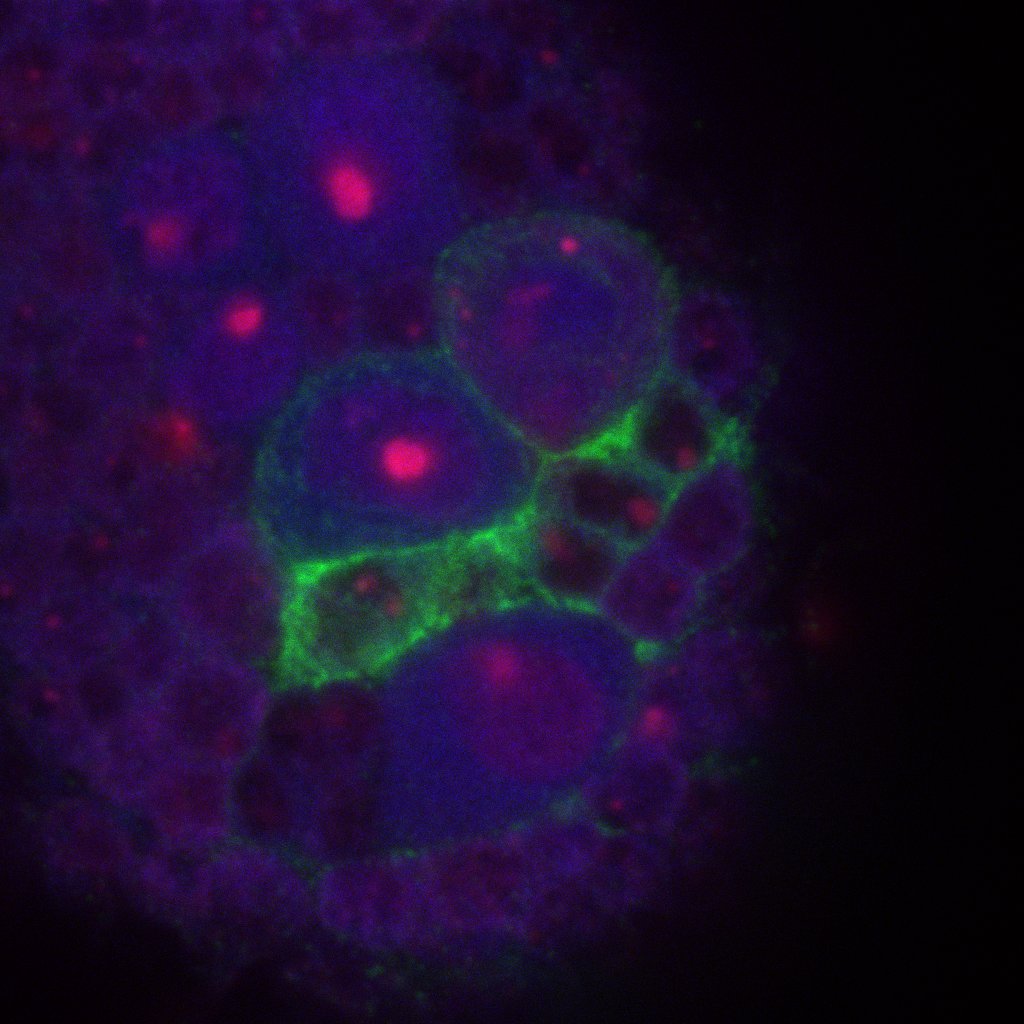

Supplement: Supplementary file 9 — Source Data Fig. 4 [file 44319_2023_17_MOESM9_ESM.zip › Fig4_source data/Fig4A_source data.jpg]

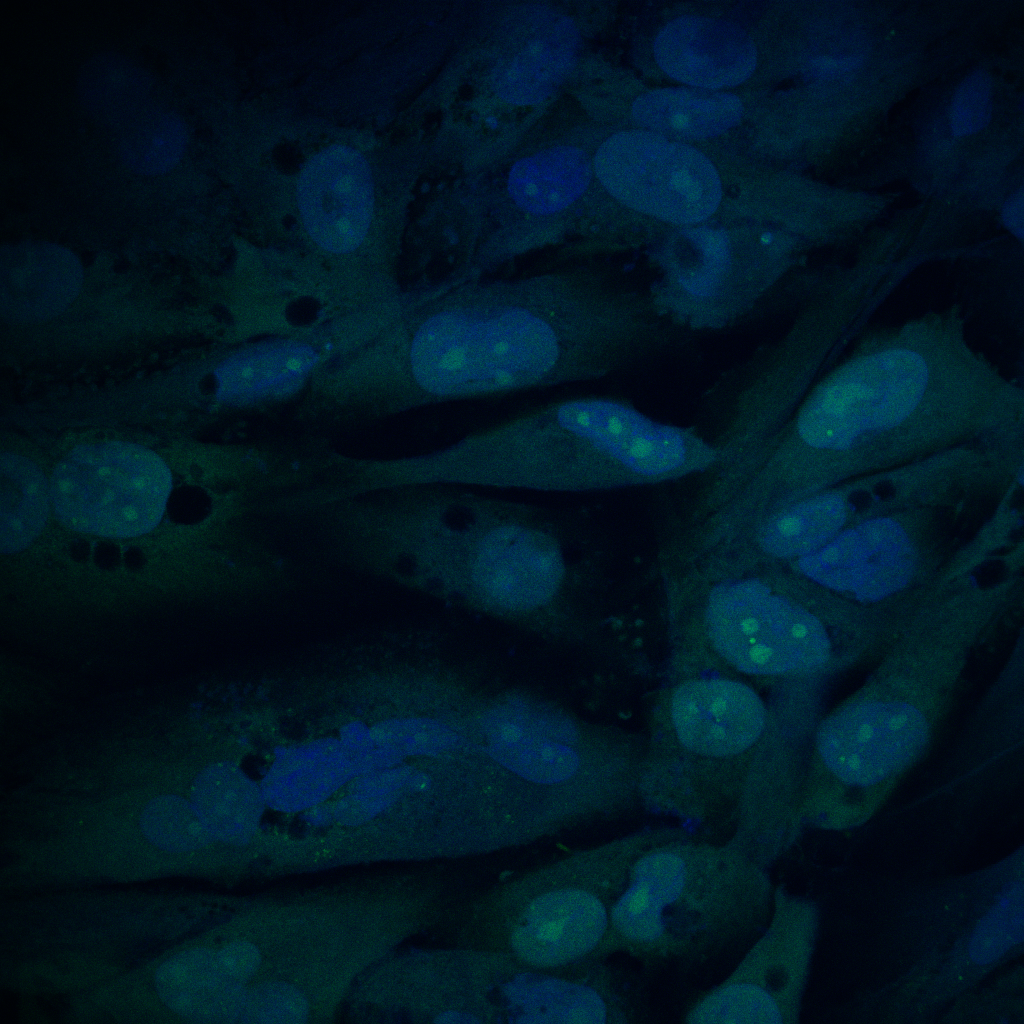

Supplement: Supplementary file 10 — Source Data Fig. 5 [file 44319_2023_17_MOESM10_ESM.zip › Fig5_source data/Fig5L_source data.tif]

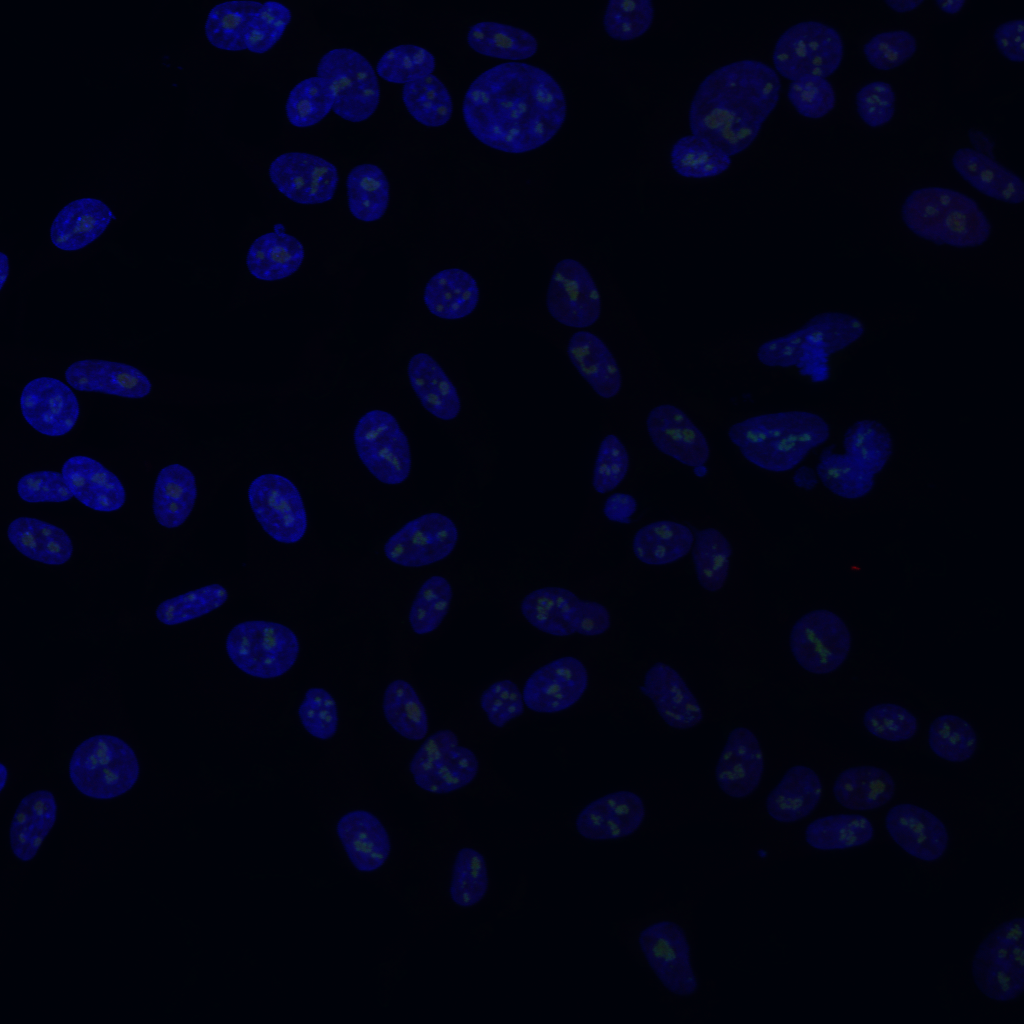

Supplement: Supplementary file 10 — Source Data Fig. 5 [file 44319_2023_17_MOESM10_ESM.zip › Fig5_source data/Fig5C_source data.tif]

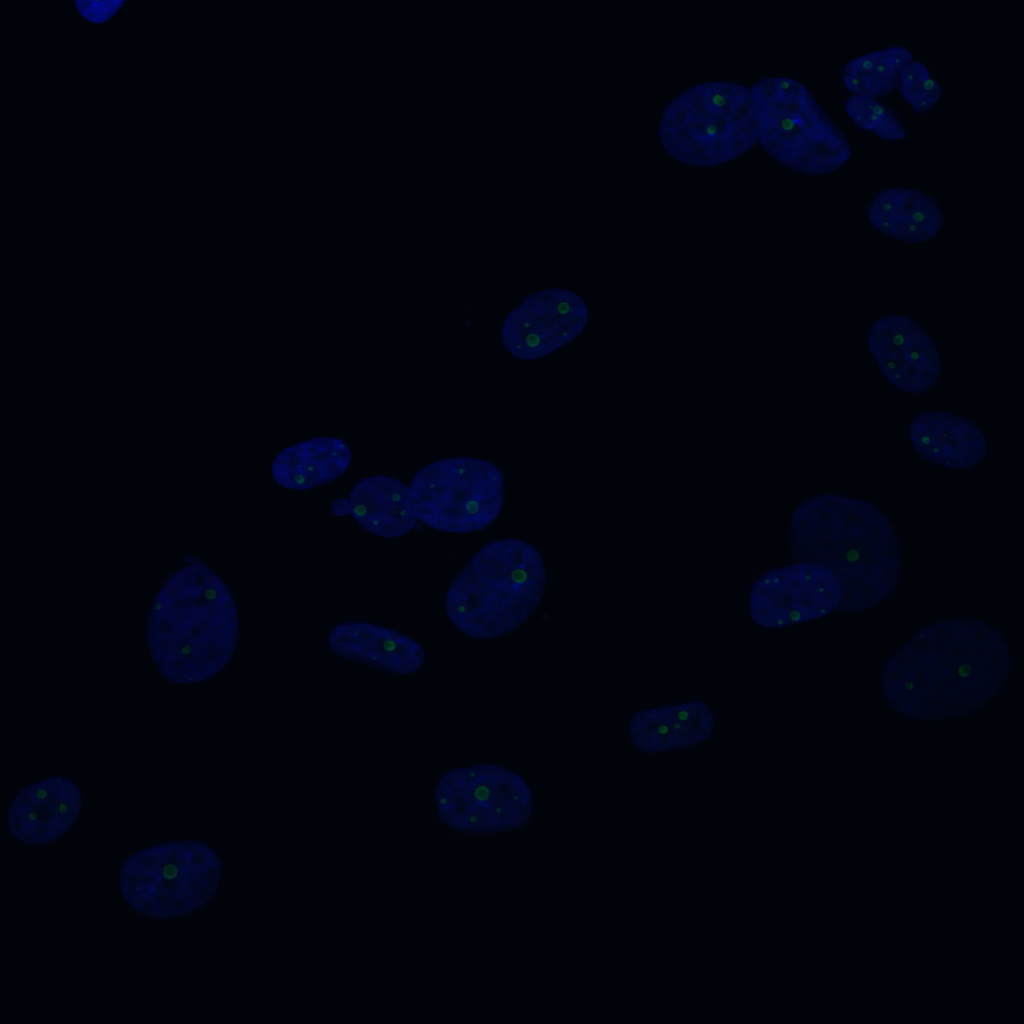

Supplement: Supplementary file 10 — Source Data Fig. 5 [file 44319_2023_17_MOESM10_ESM.zip › Fig5_source data/Fig5D_source data.tif]

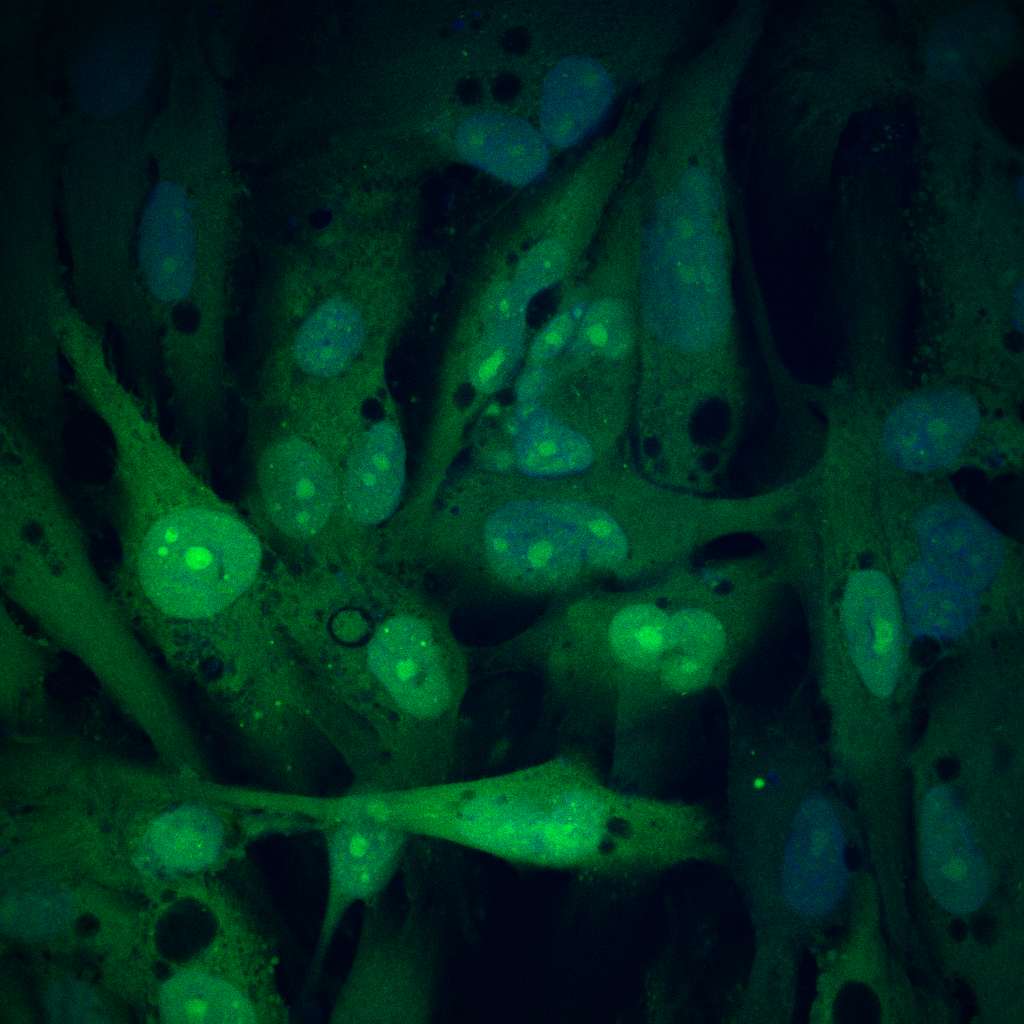

Supplement: Supplementary file 10 — Source Data Fig. 5 [file 44319_2023_17_MOESM10_ESM.zip › Fig5_source data/Fig5K_source data.tif]

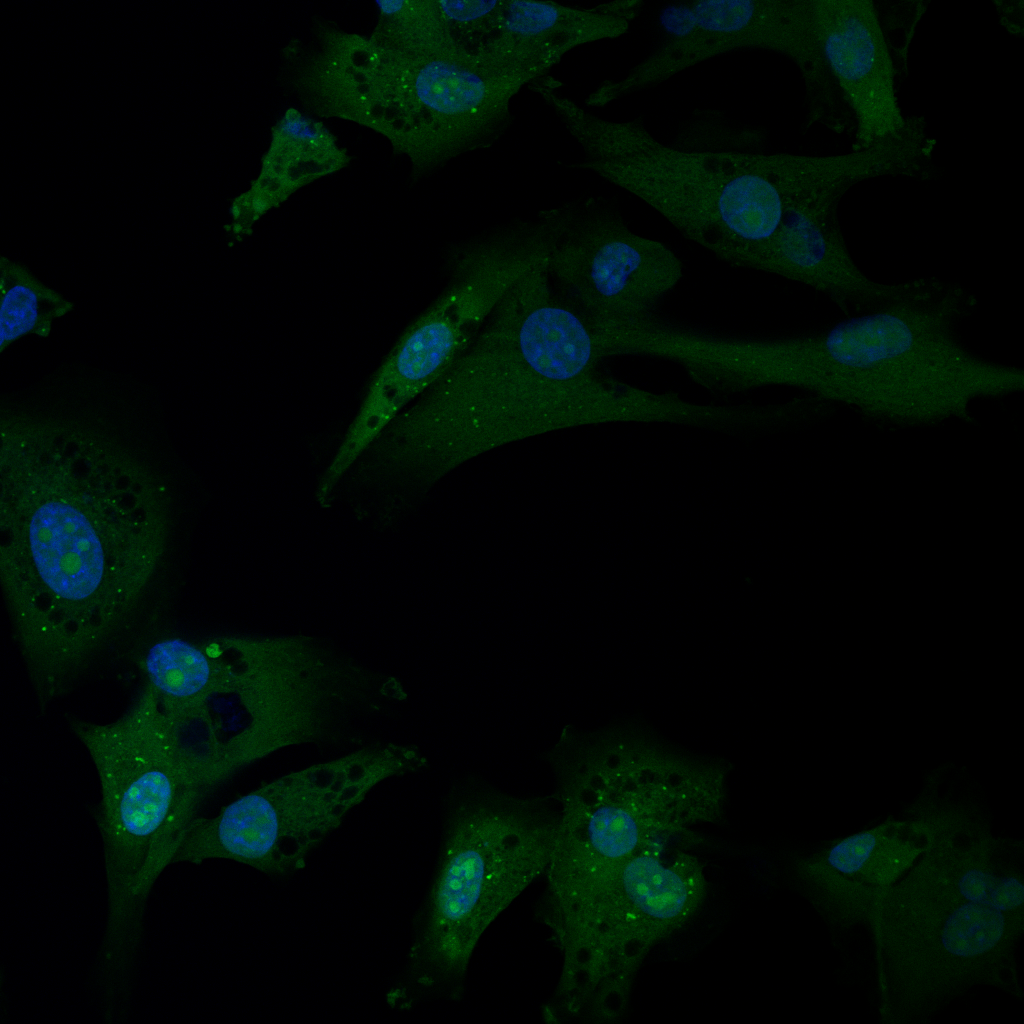

Supplement: Supplementary file 10 — Source Data Fig. 5 [file 44319_2023_17_MOESM10_ESM.zip › Fig5_source data/Fig5J_source data.tif]

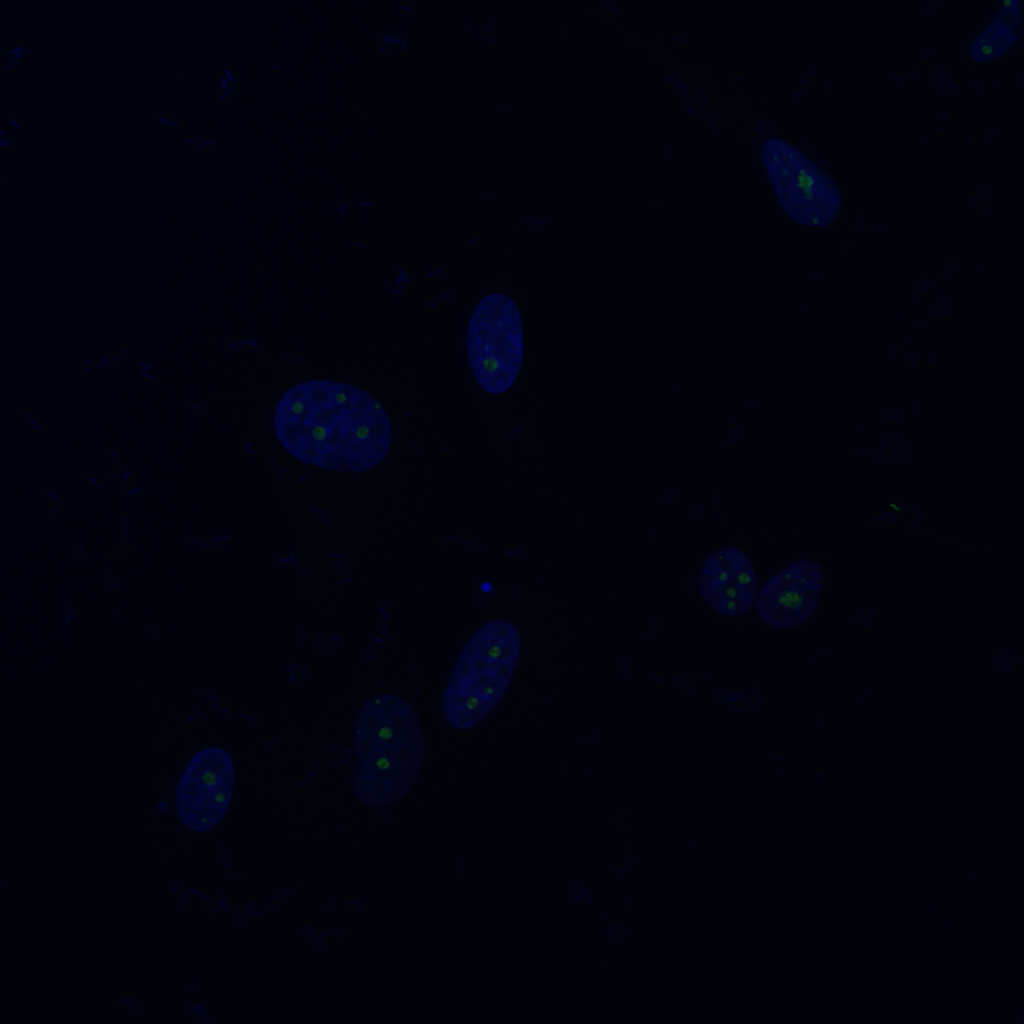

Supplement: Supplementary file 10 — Source Data Fig. 5 [file 44319_2023_17_MOESM10_ESM.zip › Fig5_source data/Fig5B_source data.tif]

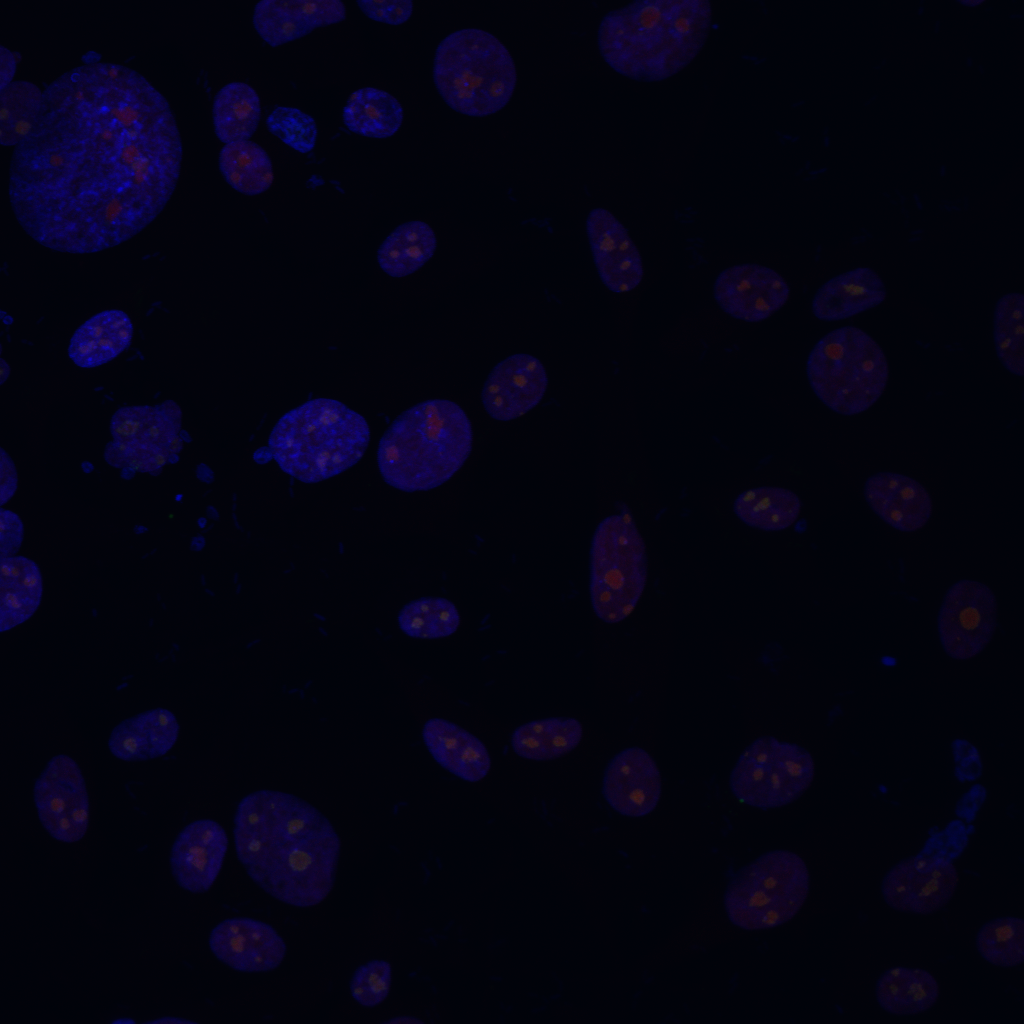

Supplement: Supplementary file 10 — Source Data Fig. 5 [file 44319_2023_17_MOESM10_ESM.zip › Fig5_source data/Fig5A_source data.tif]

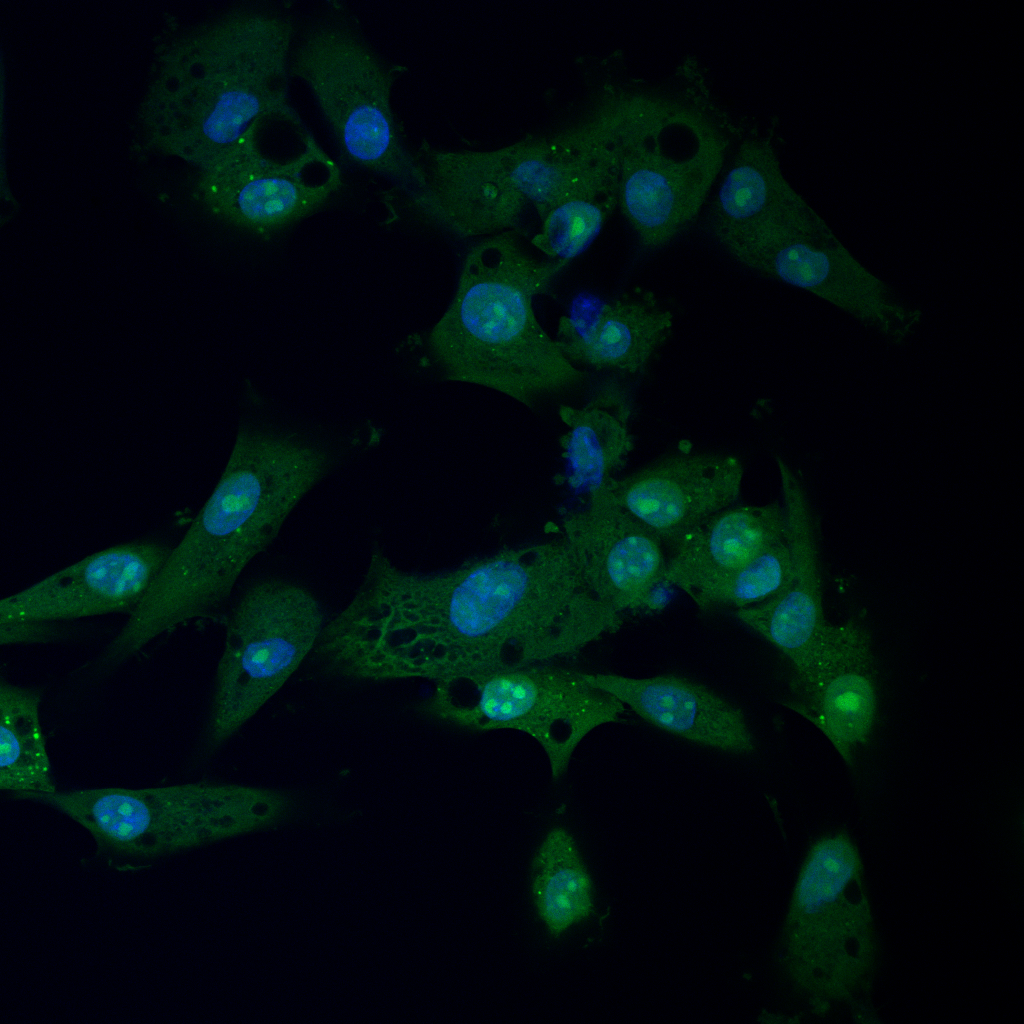

Supplement: Supplementary file 10 — Source Data Fig. 5 [file 44319_2023_17_MOESM10_ESM.zip › Fig5_source data/Fig5I_source data.tif]

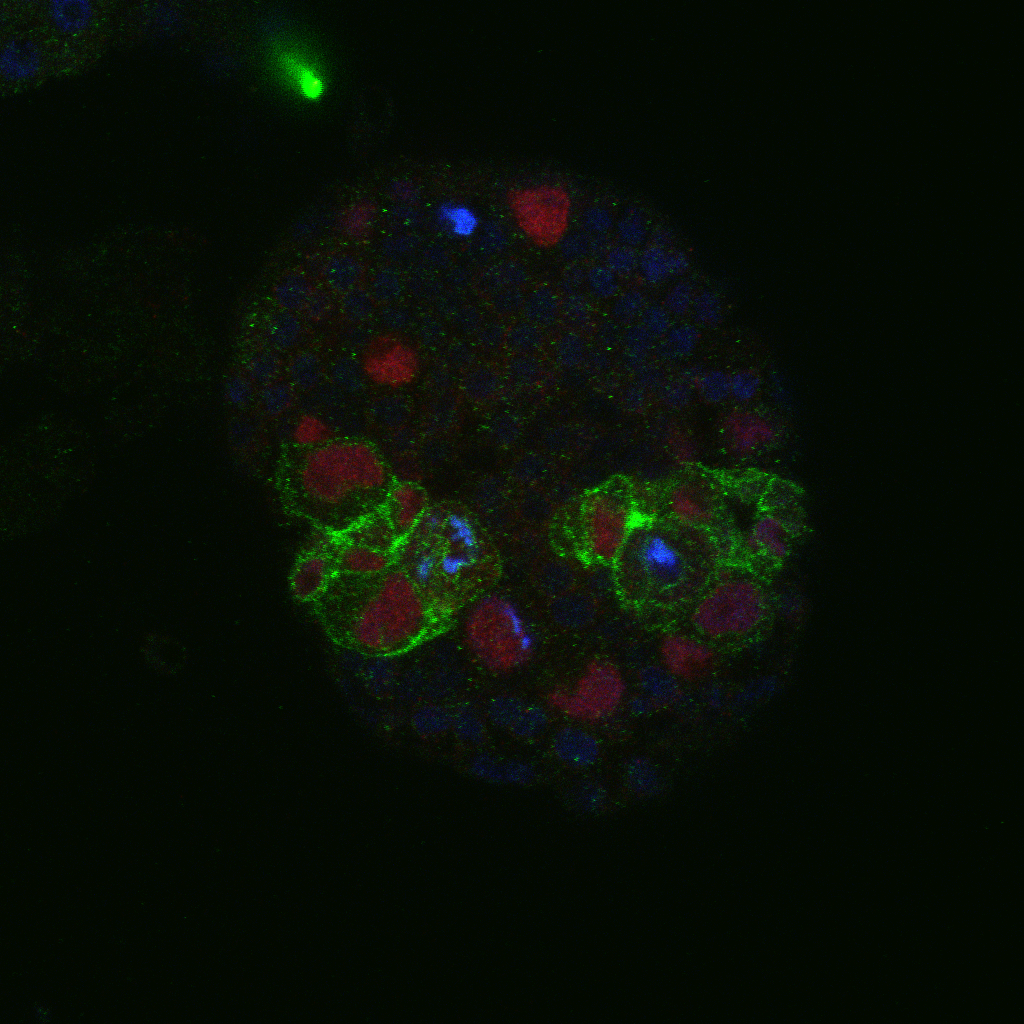

Supplement: Supplementary file 11 — Source Data Fig. 6 [file 44319_2023_17_MOESM11_ESM.zip › Fig6_source data/Fig6A_source data.tif]

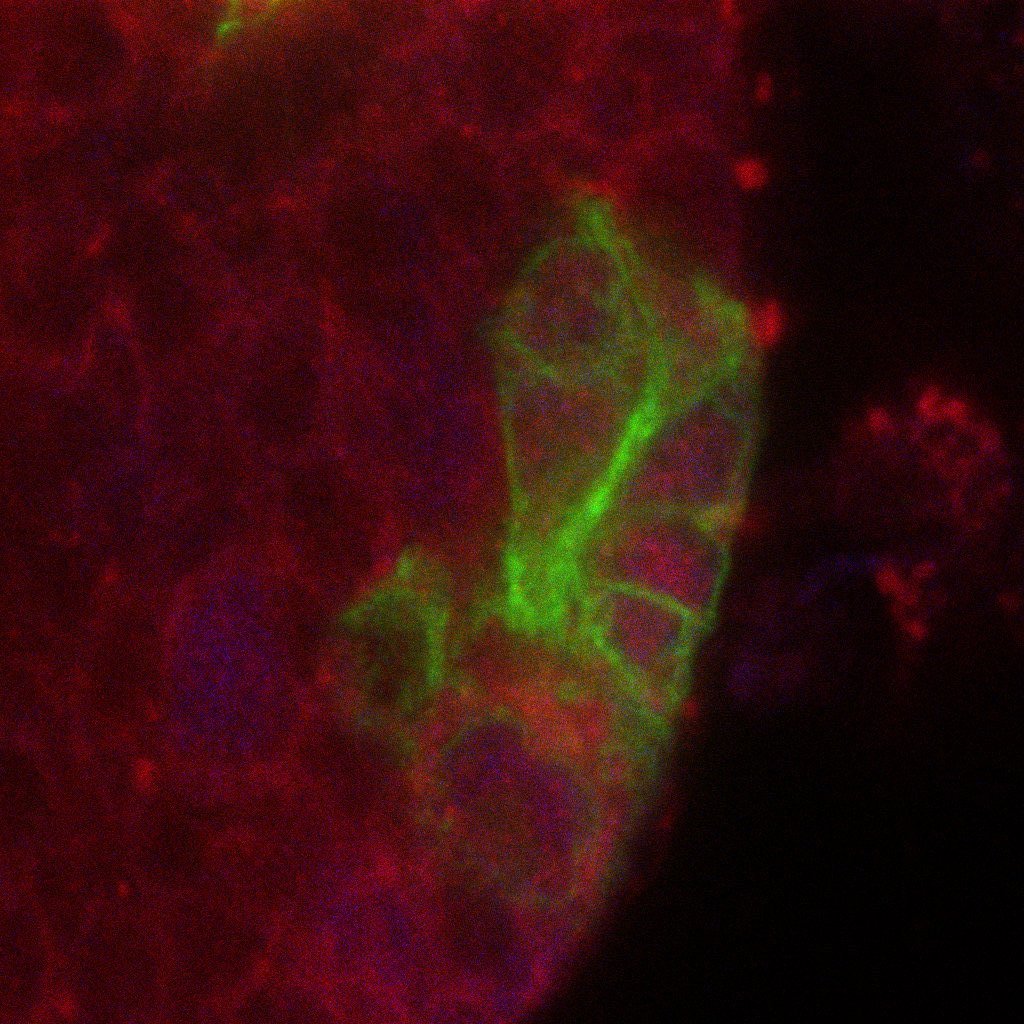

Supplement: Supplementary file 11 — Source Data Fig. 6 [file 44319_2023_17_MOESM11_ESM.zip › Fig6_source data/Fig6O_source data.jpg]

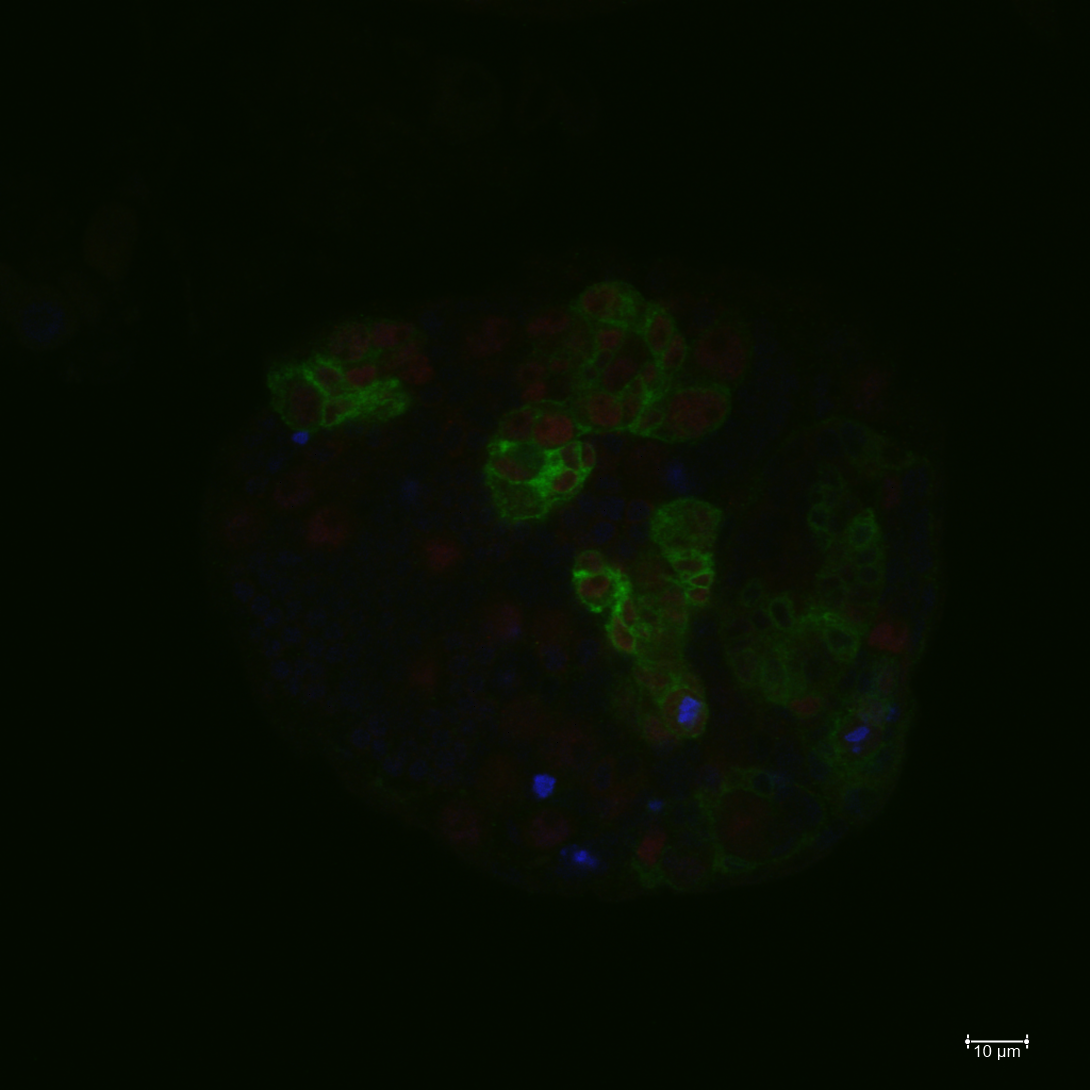

Supplement: Supplementary file 11 — Source Data Fig. 6 [file 44319_2023_17_MOESM11_ESM.zip › Fig6_source data/Fig6F_source data.tif]

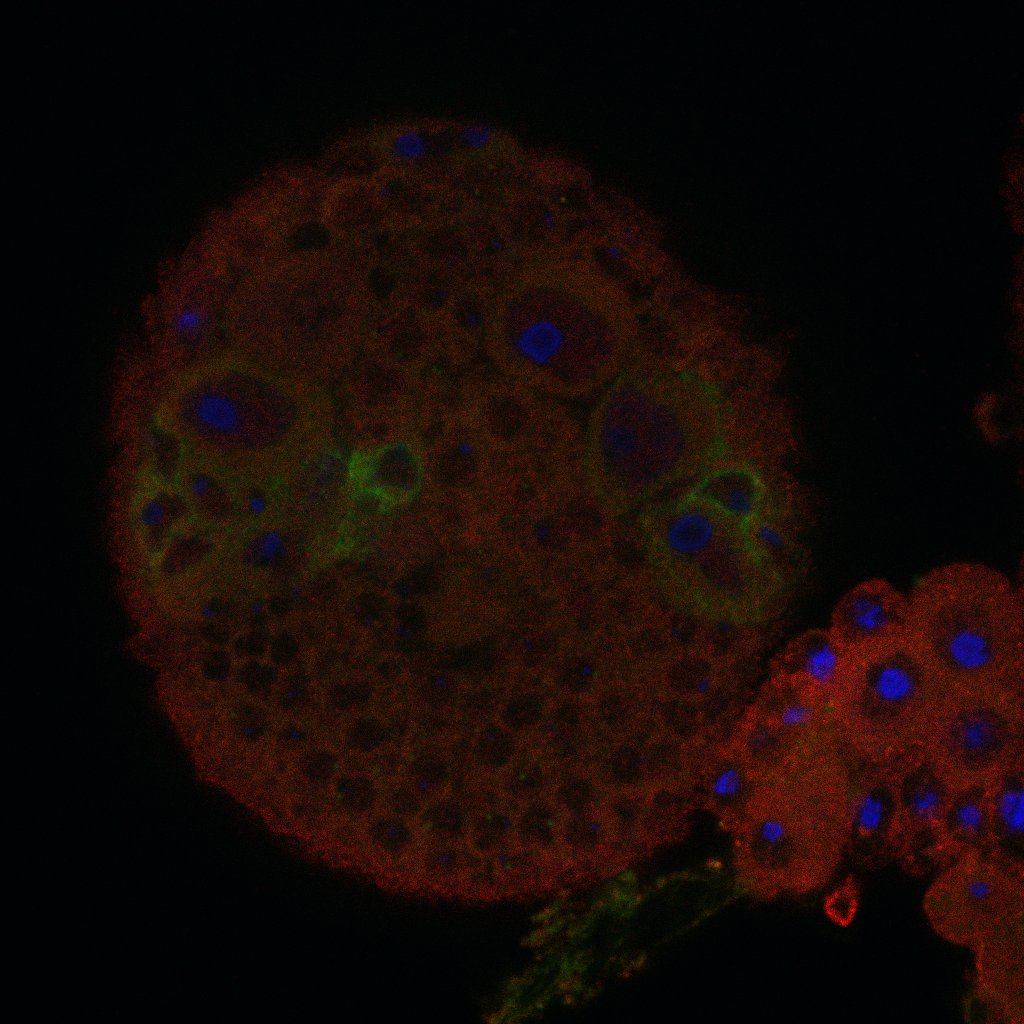

Supplement: Supplementary file 11 — Source Data Fig. 6 [file 44319_2023_17_MOESM11_ESM.zip › Fig6_source data/Fig6R_source data.jpg]

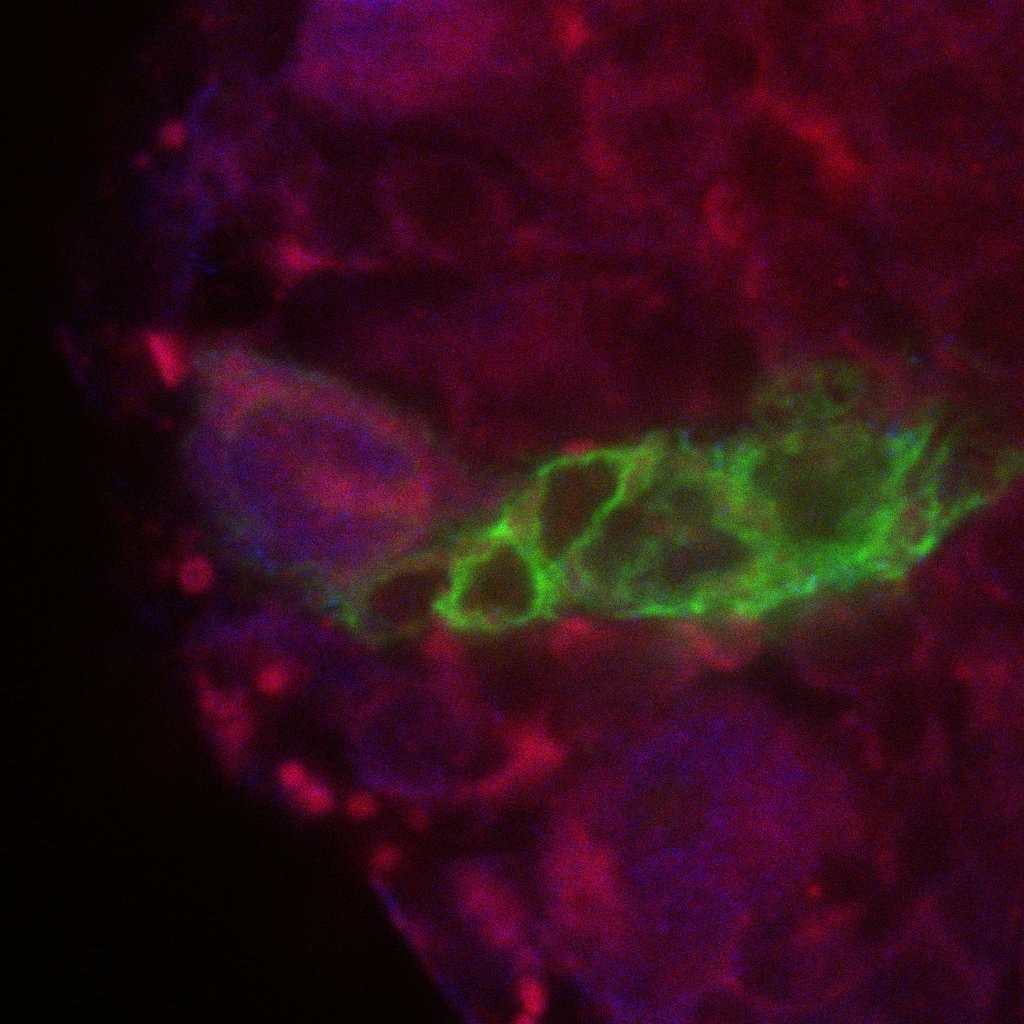

Supplement: Supplementary file 11 — Source Data Fig. 6 [file 44319_2023_17_MOESM11_ESM.zip › Fig6_source data/Fig6N_source data.jpg]

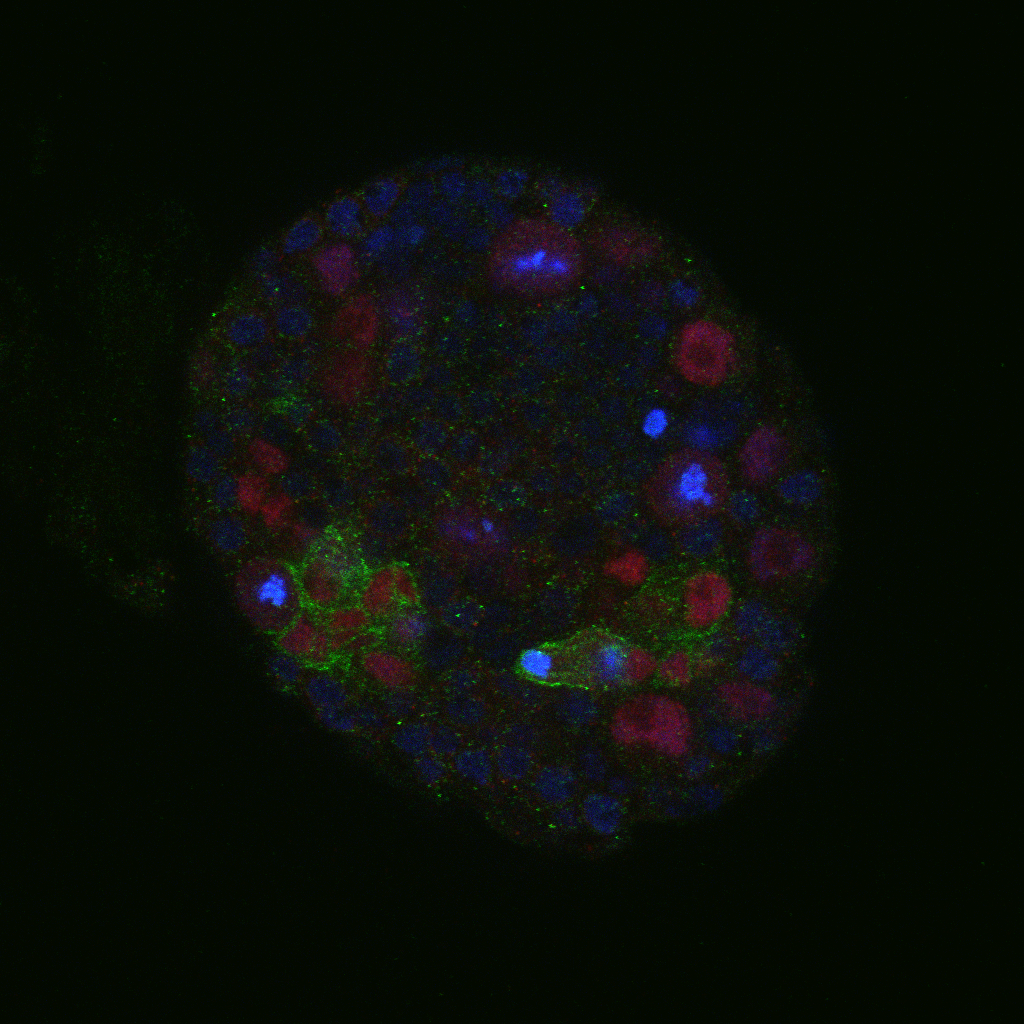

Supplement: Supplementary file 11 — Source Data Fig. 6 [file 44319_2023_17_MOESM11_ESM.zip › Fig6_source data/Fig6B_source data..tif]

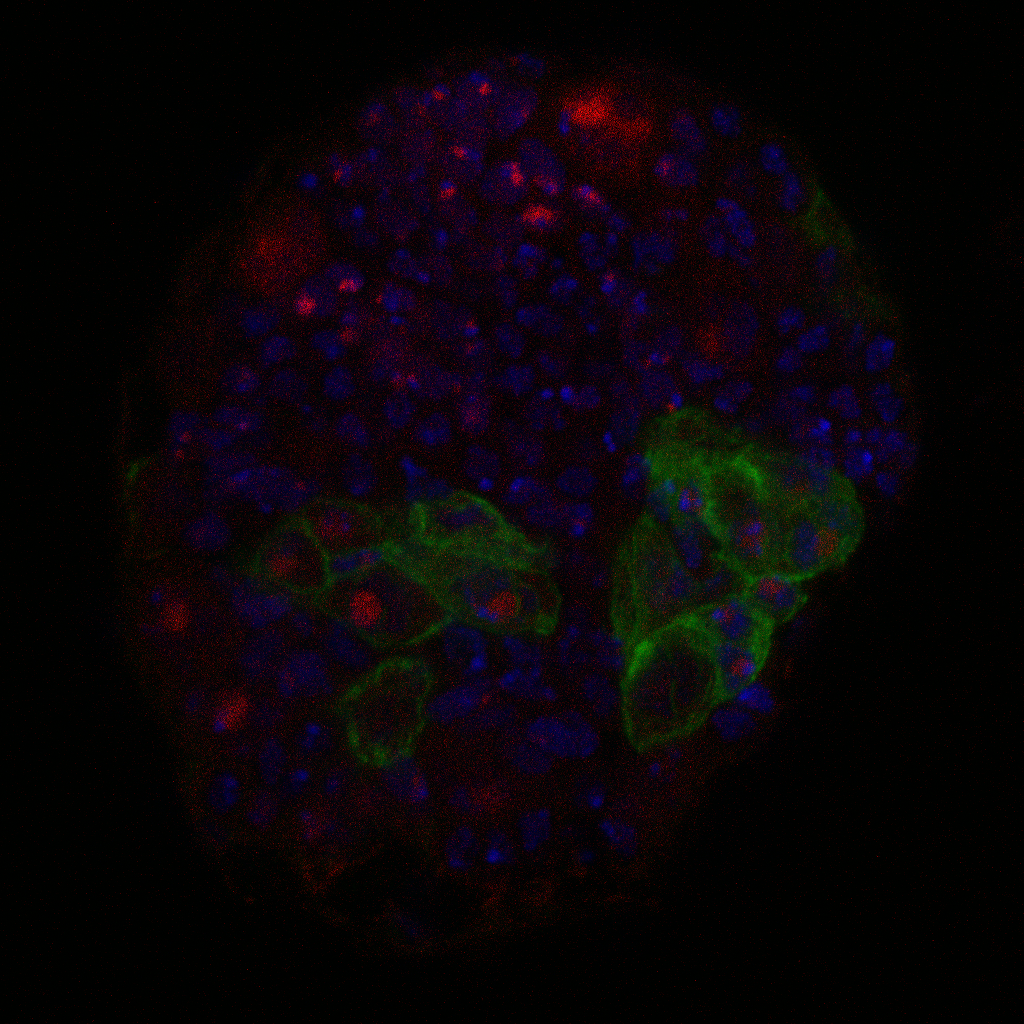

Supplement: Supplementary file 11 — Source Data Fig. 6 [file 44319_2023_17_MOESM11_ESM.zip › Fig6_source data/Fig6J_source data.tif]

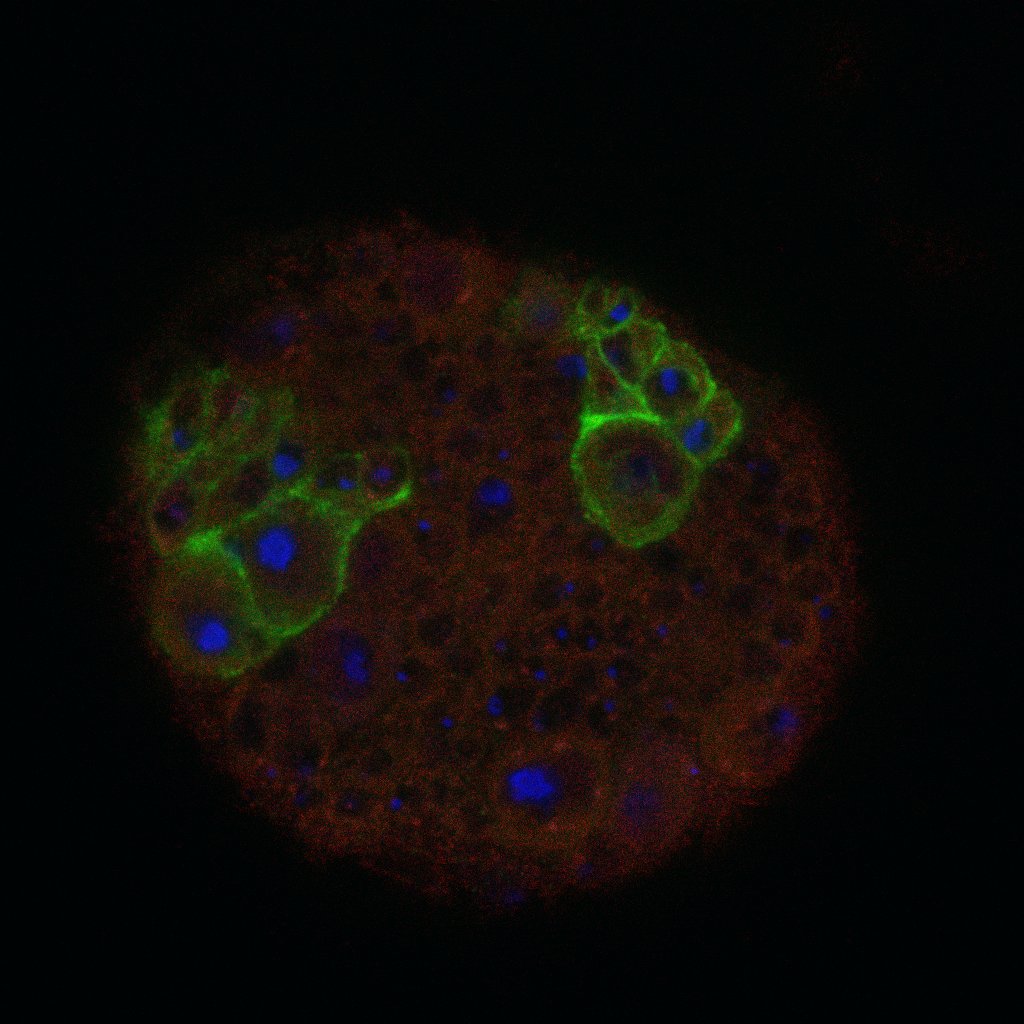

Supplement: Supplementary file 11 — Source Data Fig. 6 [file 44319_2023_17_MOESM11_ESM.zip › Fig6_source data/Fig6Q_source data.jpg]

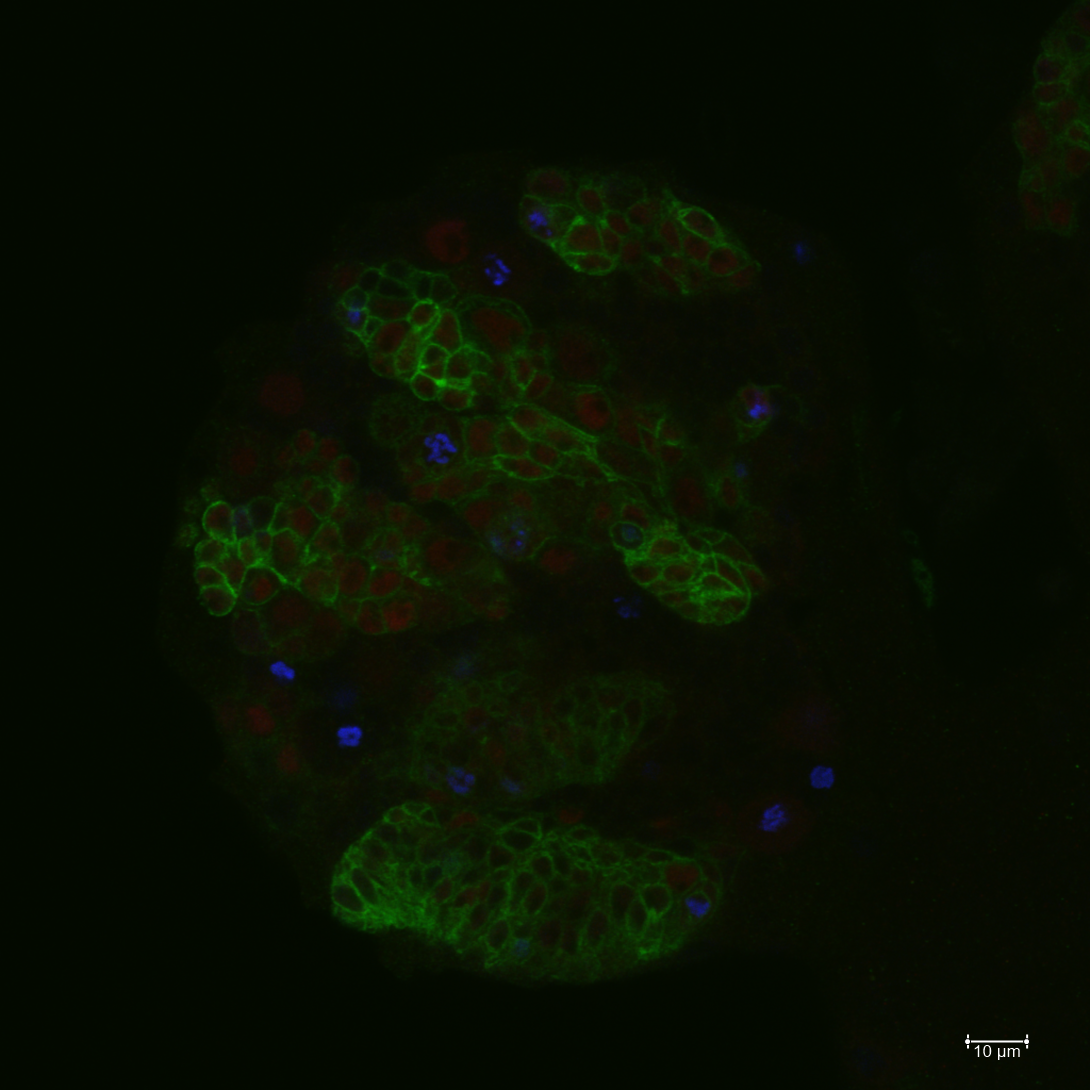

Supplement: Supplementary file 11 — Source Data Fig. 6 [file 44319_2023_17_MOESM11_ESM.zip › Fig6_source data/Fig6E_source data.tif]

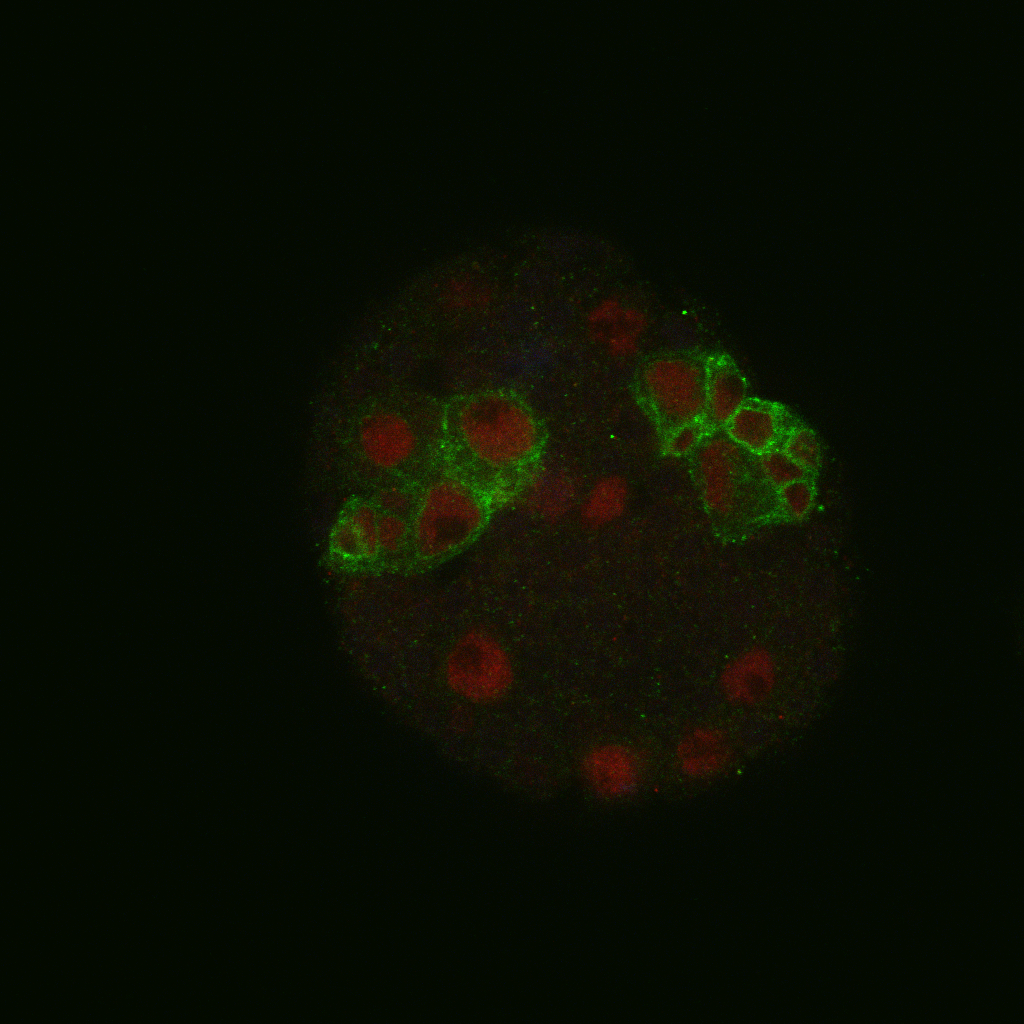

Supplement: Supplementary file 11 — Source Data Fig. 6 [file 44319_2023_17_MOESM11_ESM.zip › Fig6_source data/Fig6C_source data.tif]

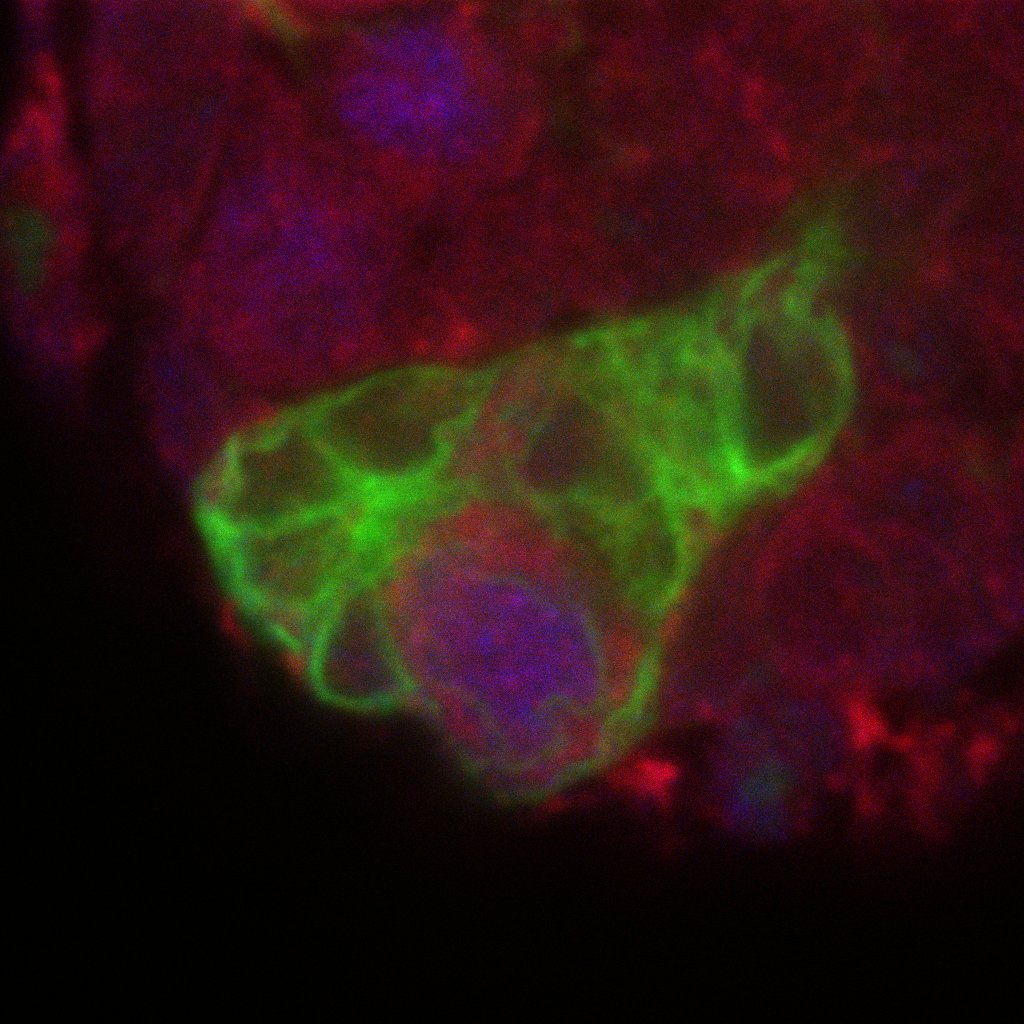

Supplement: Supplementary file 11 — Source Data Fig. 6 [file 44319_2023_17_MOESM11_ESM.zip › Fig6_source data/Fig6M_source data.jpg]

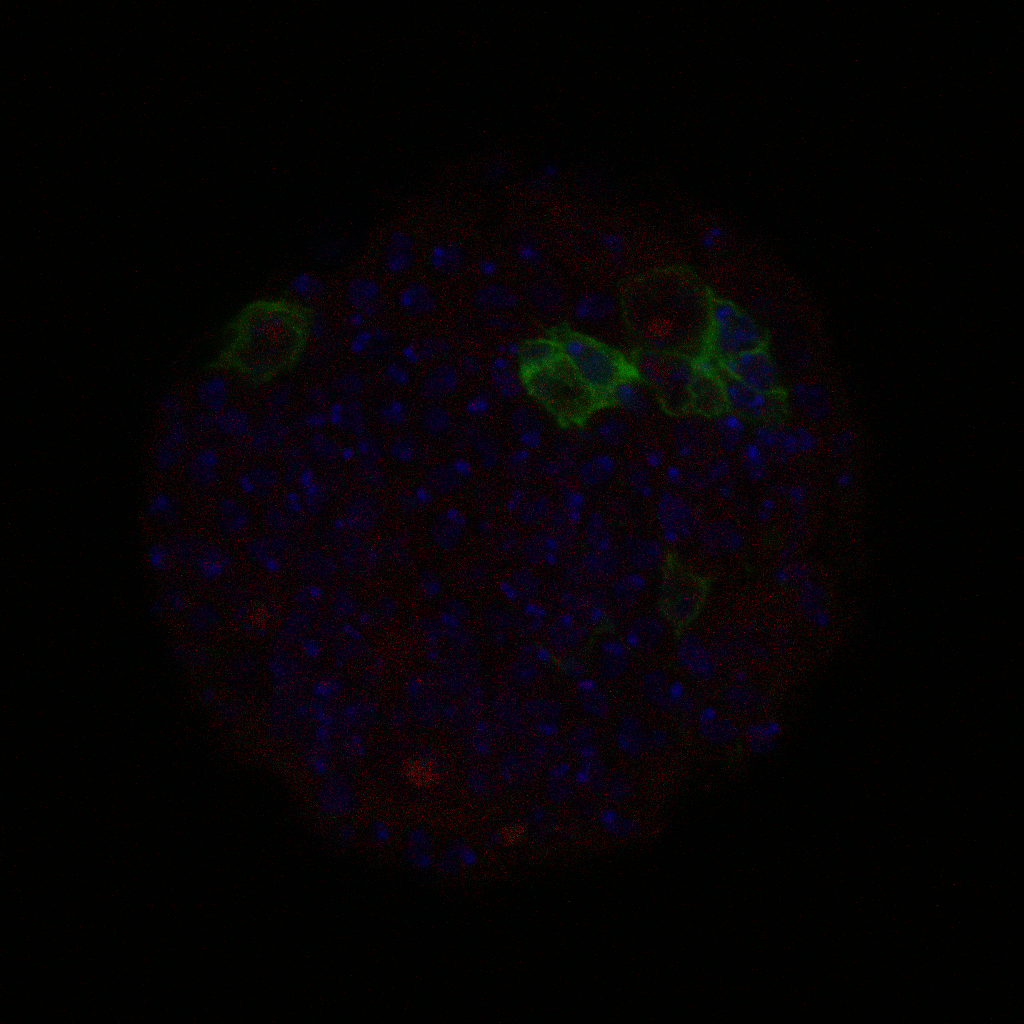

Supplement: Supplementary file 11 — Source Data Fig. 6 [file 44319_2023_17_MOESM11_ESM.zip › Fig6_source data/Fig6K_source data.tif]

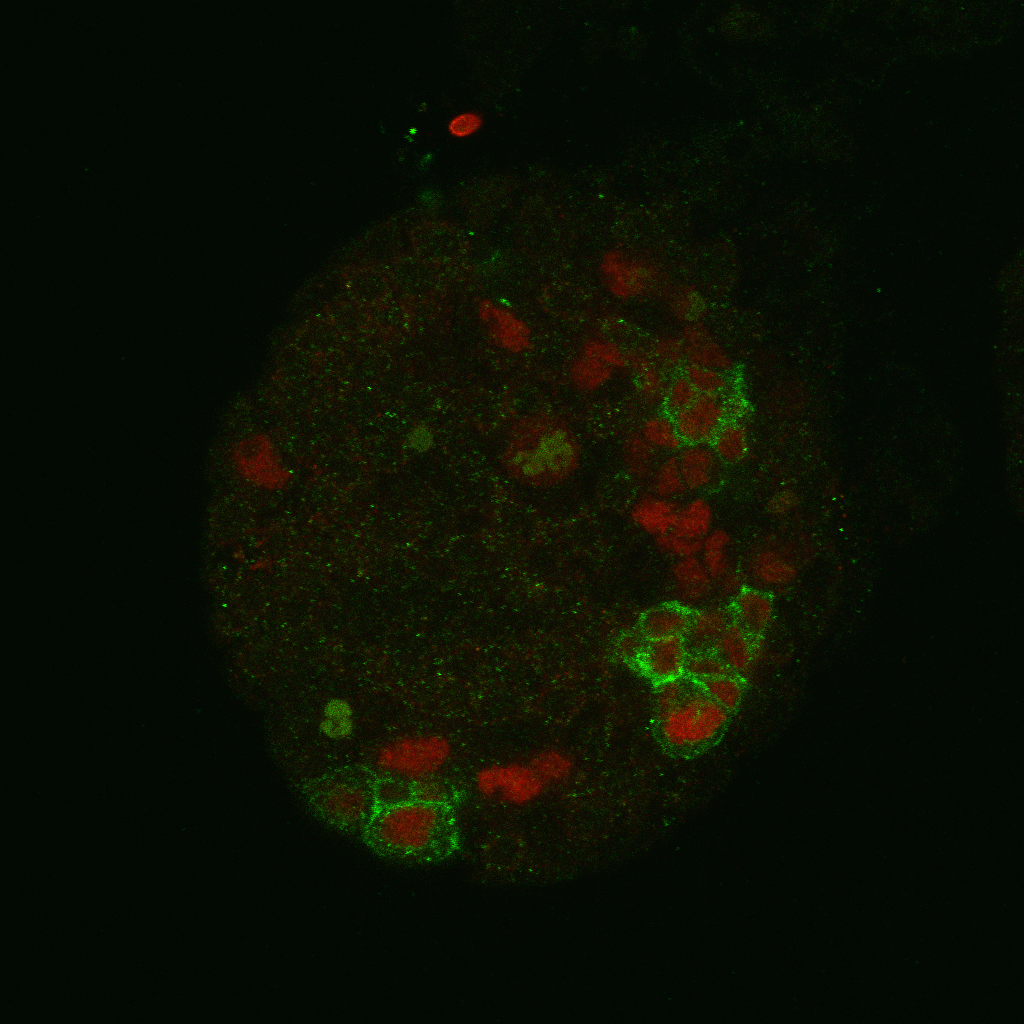

Supplement: Supplementary file 11 — Source Data Fig. 6 [file 44319_2023_17_MOESM11_ESM.zip › Fig6_source data/Fig6D_source data.tif]

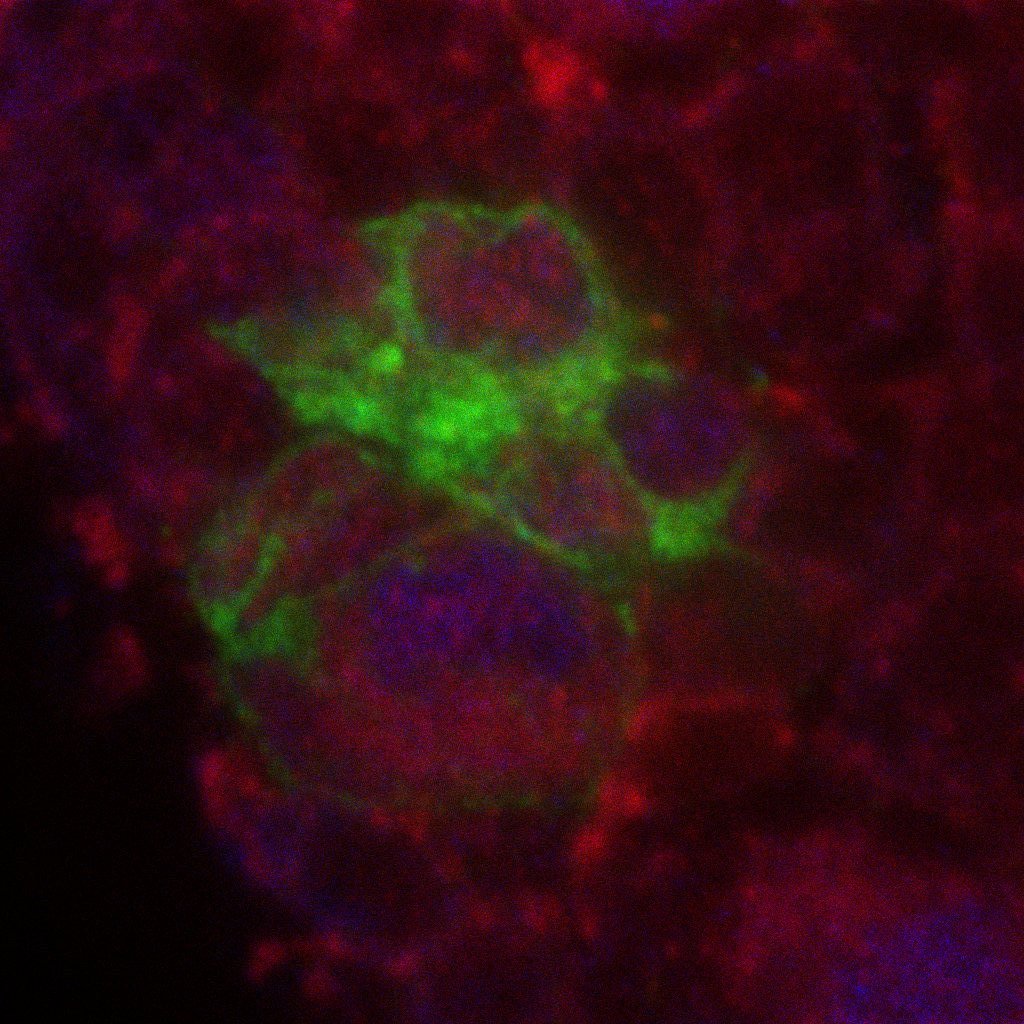

Supplement: Supplementary file 11 — Source Data Fig. 6 [file 44319_2023_17_MOESM11_ESM.zip › Fig6_source data/Fig6P_source data.jpg]

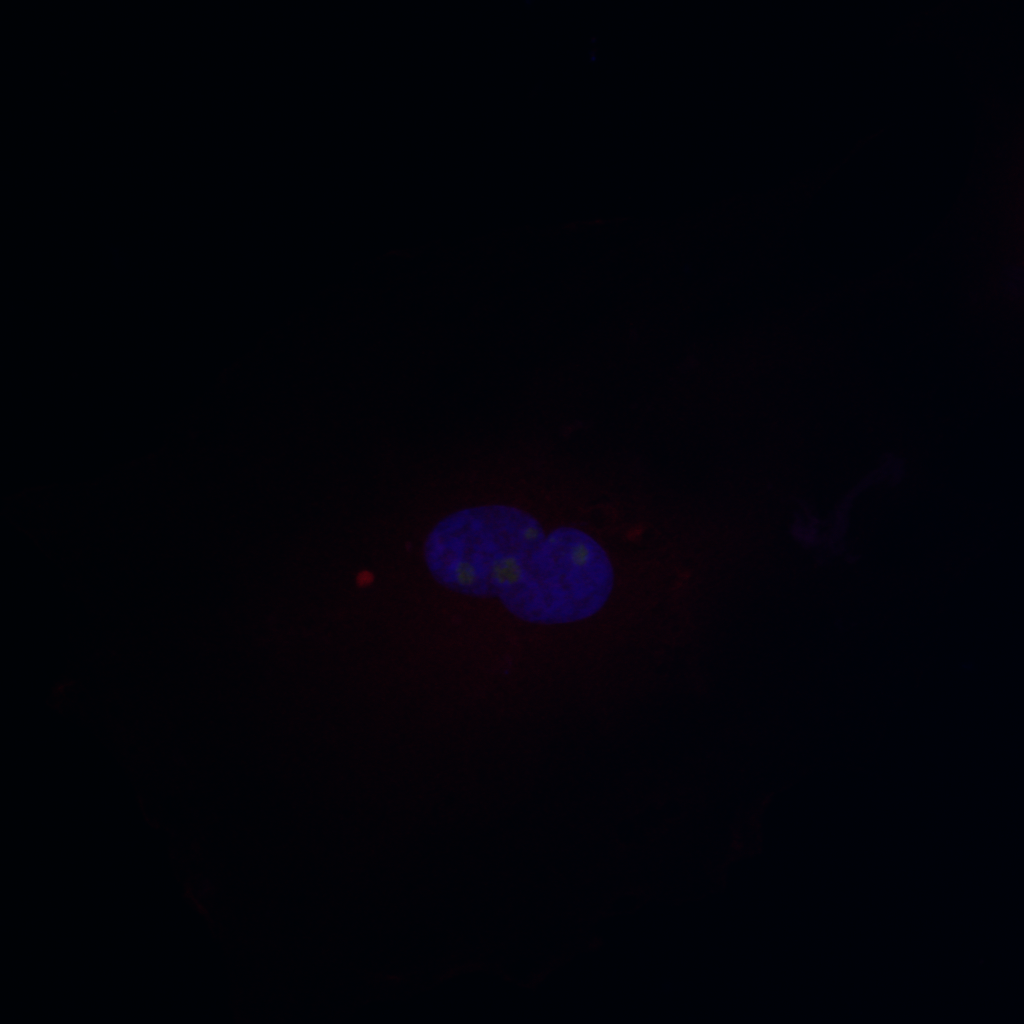

Supplement: Supplementary file 12 — Source Data Fig. 7 [file 44319_2023_17_MOESM12_ESM.zip › 44319_2023_17_MOESM12_ESM/Fig7_source data/Fig7A_source data.tif]

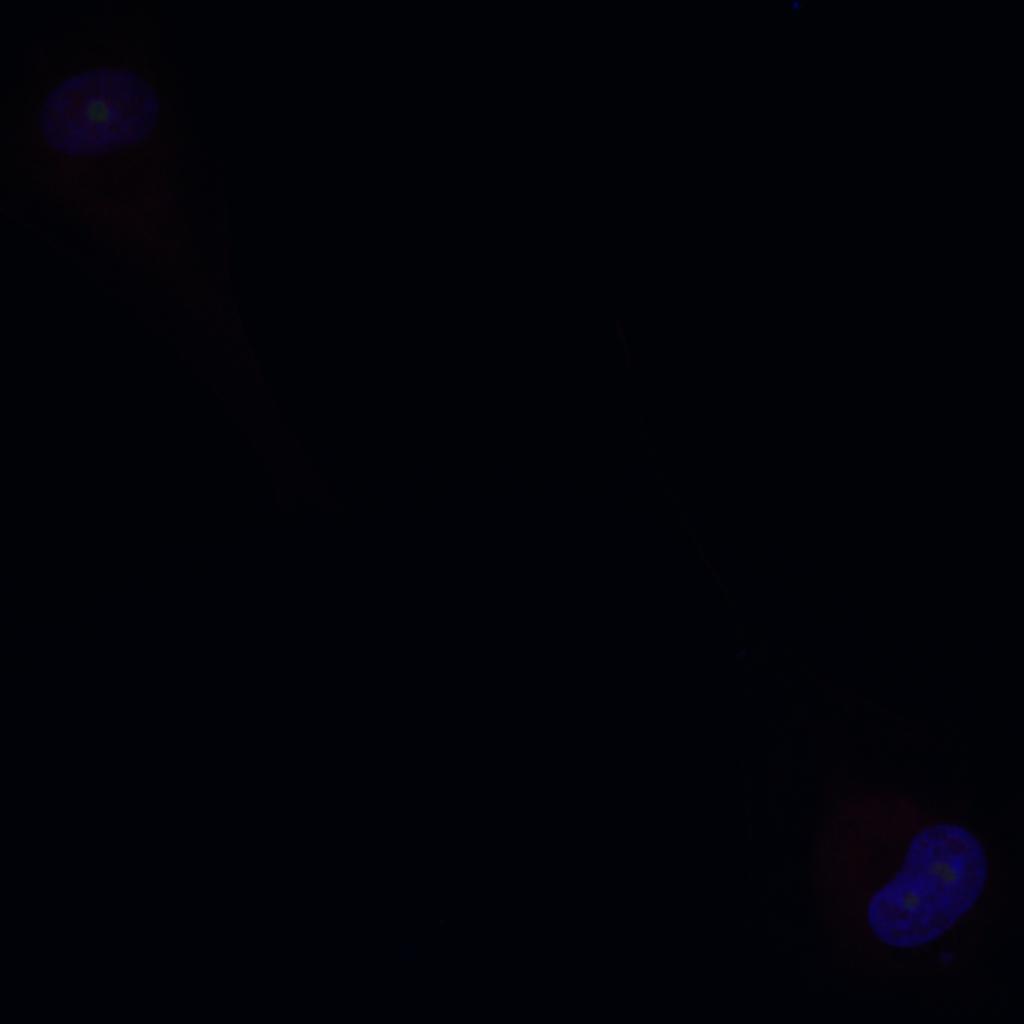

Supplement: Supplementary file 12 — Source Data Fig. 7 [file 44319_2023_17_MOESM12_ESM.zip › 44319_2023_17_MOESM12_ESM/Fig7_source data/Fig7B_source data.tif]

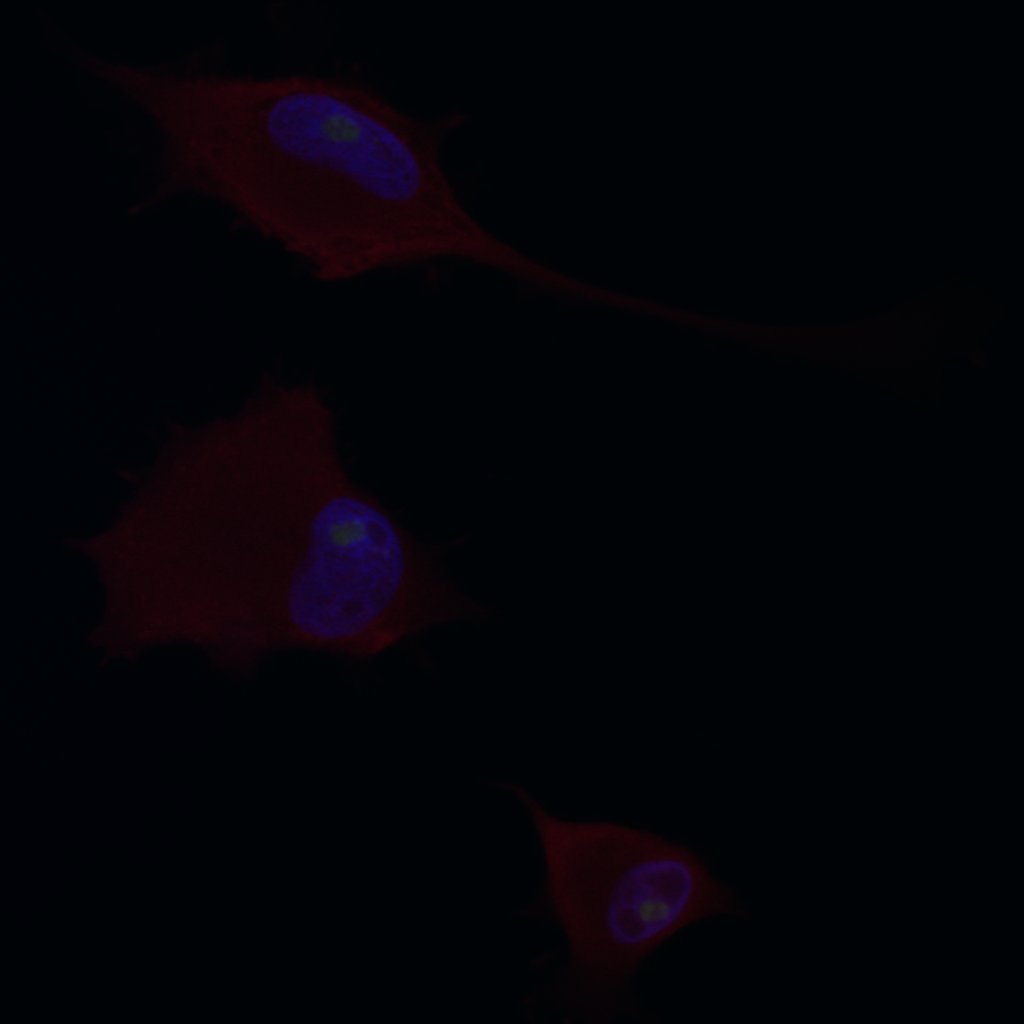

Supplement: Supplementary file 12 — Source Data Fig. 7 [file 44319_2023_17_MOESM12_ESM.zip › 44319_2023_17_MOESM12_ESM/Fig7_source data/Fig7C_source data.tif]

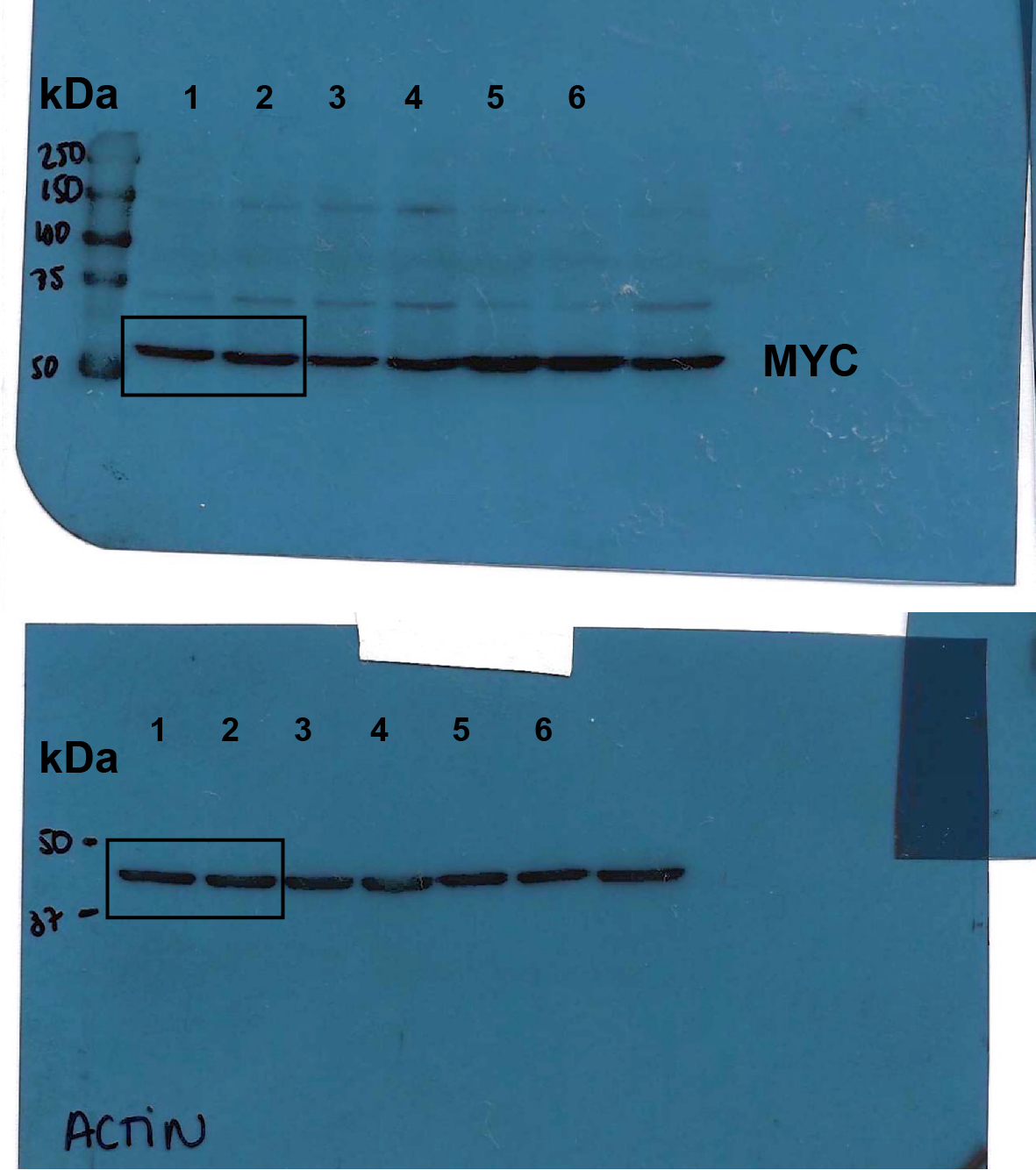

Supplement: Supplementary file 12 — Source Data Fig. 7 [file 44319_2023_17_MOESM12_ESM.zip › 44319_2023_17_MOESM12_ESM/Fig7_source data/Fig7E_source data/Fig7E_MYC+loadingcontrol_source data.jpg]

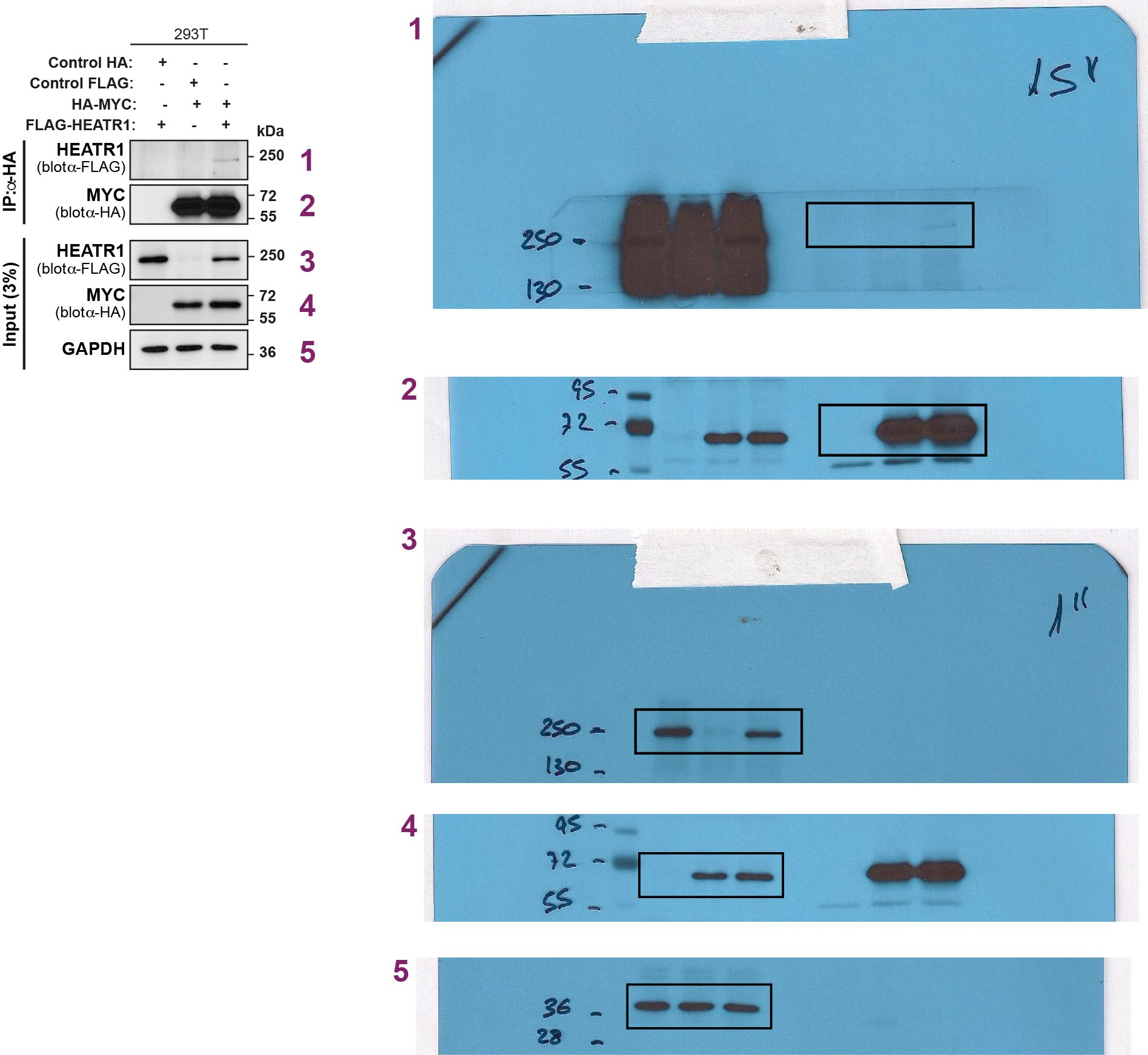

Supplement: Supplementary file 12 — Source Data Fig. 7 [file 44319_2023_17_MOESM12_ESM.zip › 44319_2023_17_MOESM12_ESM/Fig7_source data/Fig7I_source data/Fig7I_left panel_source data.jpg]

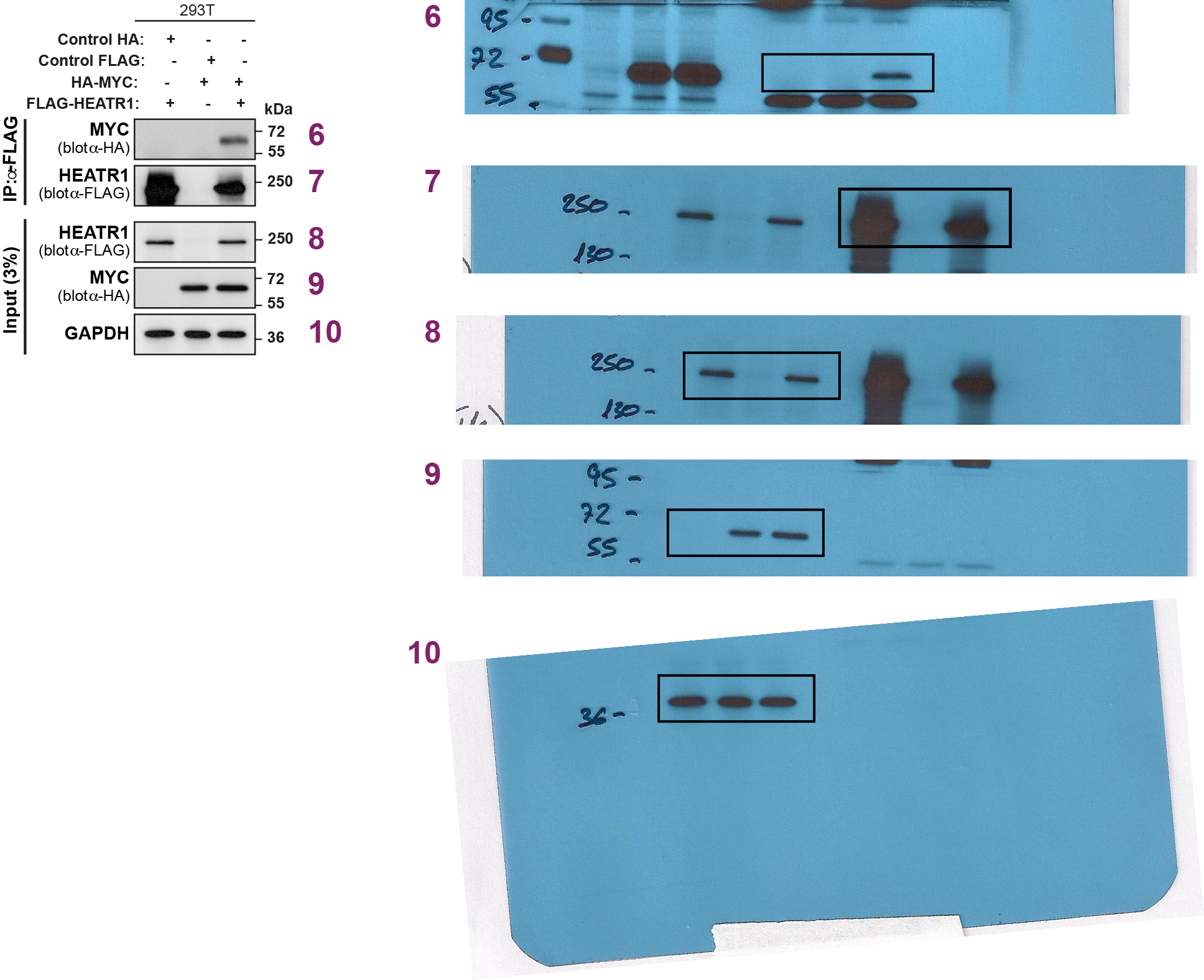

Supplement: Supplementary file 12 — Source Data Fig. 7 [file 44319_2023_17_MOESM12_ESM.zip › 44319_2023_17_MOESM12_ESM/Fig7_source data/Fig7I_source data/Fig7I_right panel_source data.jpg]

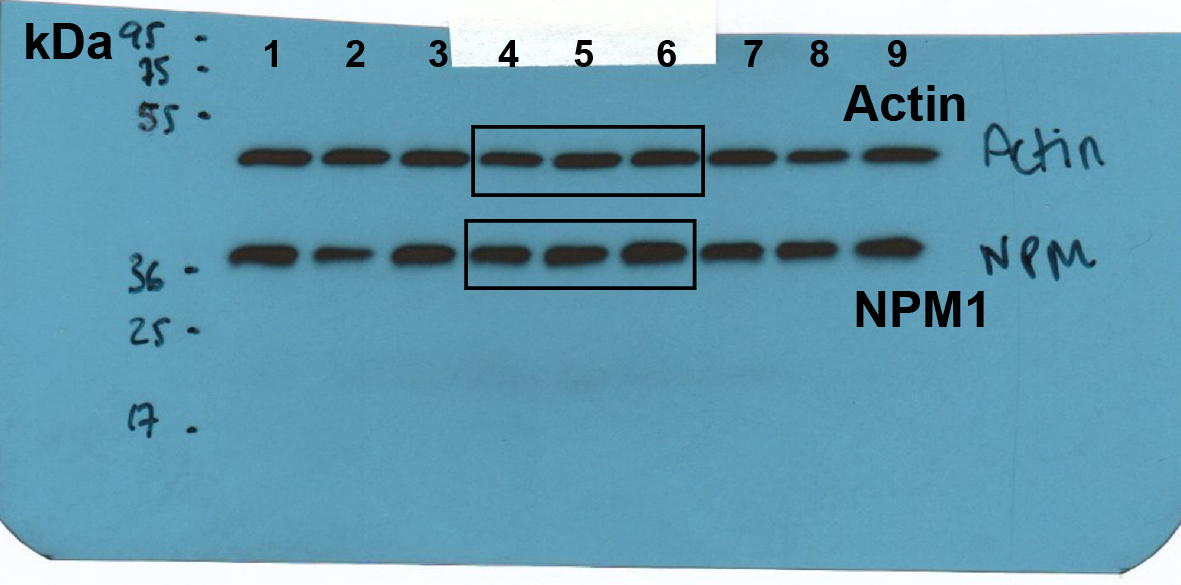

Supplement: Supplementary file 13 — Appendix Figures Source Data [file 44319_2023_17_MOESM13_ESM.zip › 44319_2023_17_MOESM13_ESM/Appendix Fig S4I_source data/Appendix FigS4I_NPM1+loading control_source data.jpg]

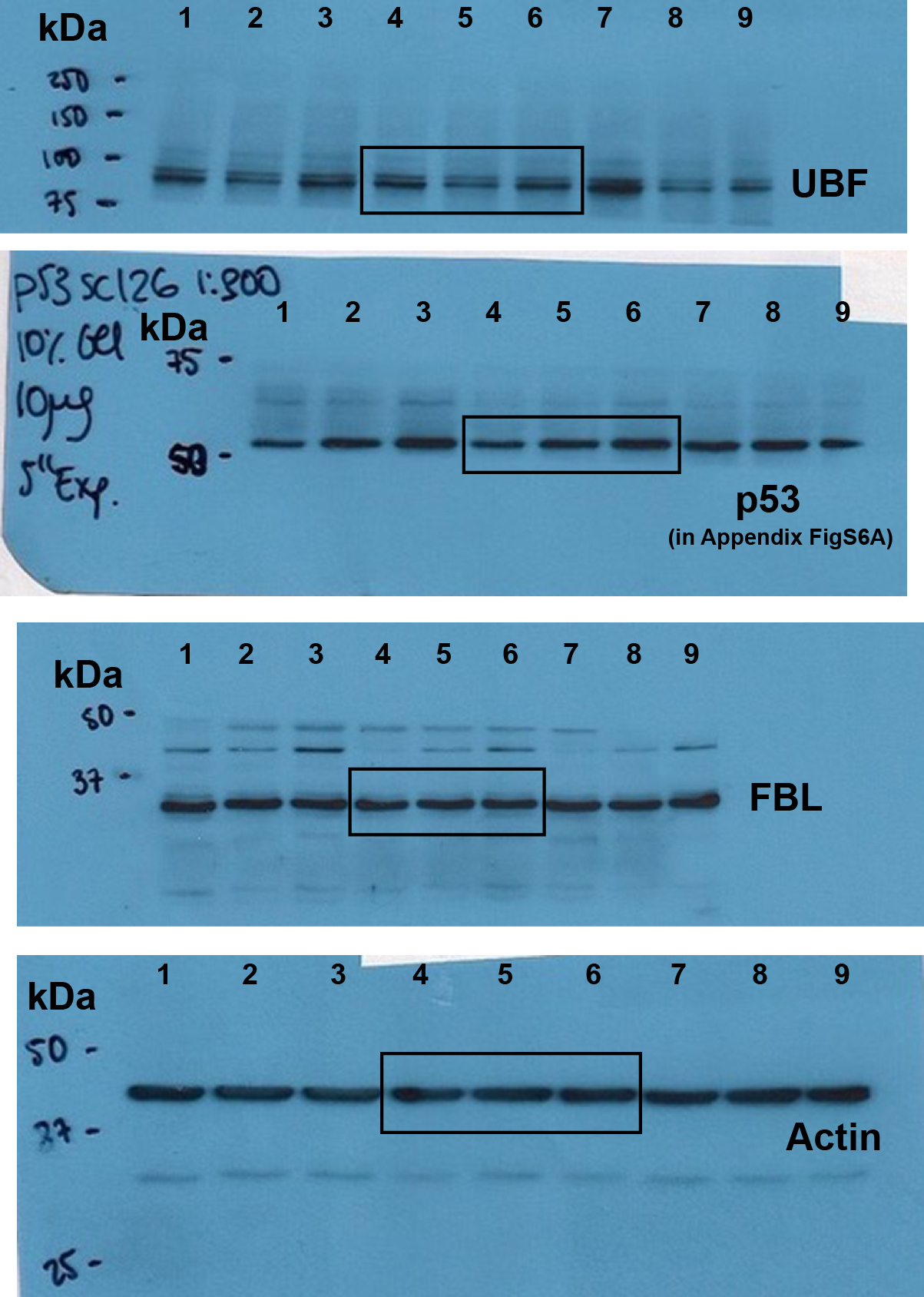

Supplement: Supplementary file 13 — Appendix Figures Source Data [file 44319_2023_17_MOESM13_ESM.zip › 44319_2023_17_MOESM13_ESM/Appendix Fig S4I_source data/Appendix FigS4I_UBF FBL+loading control_source data.jpg]

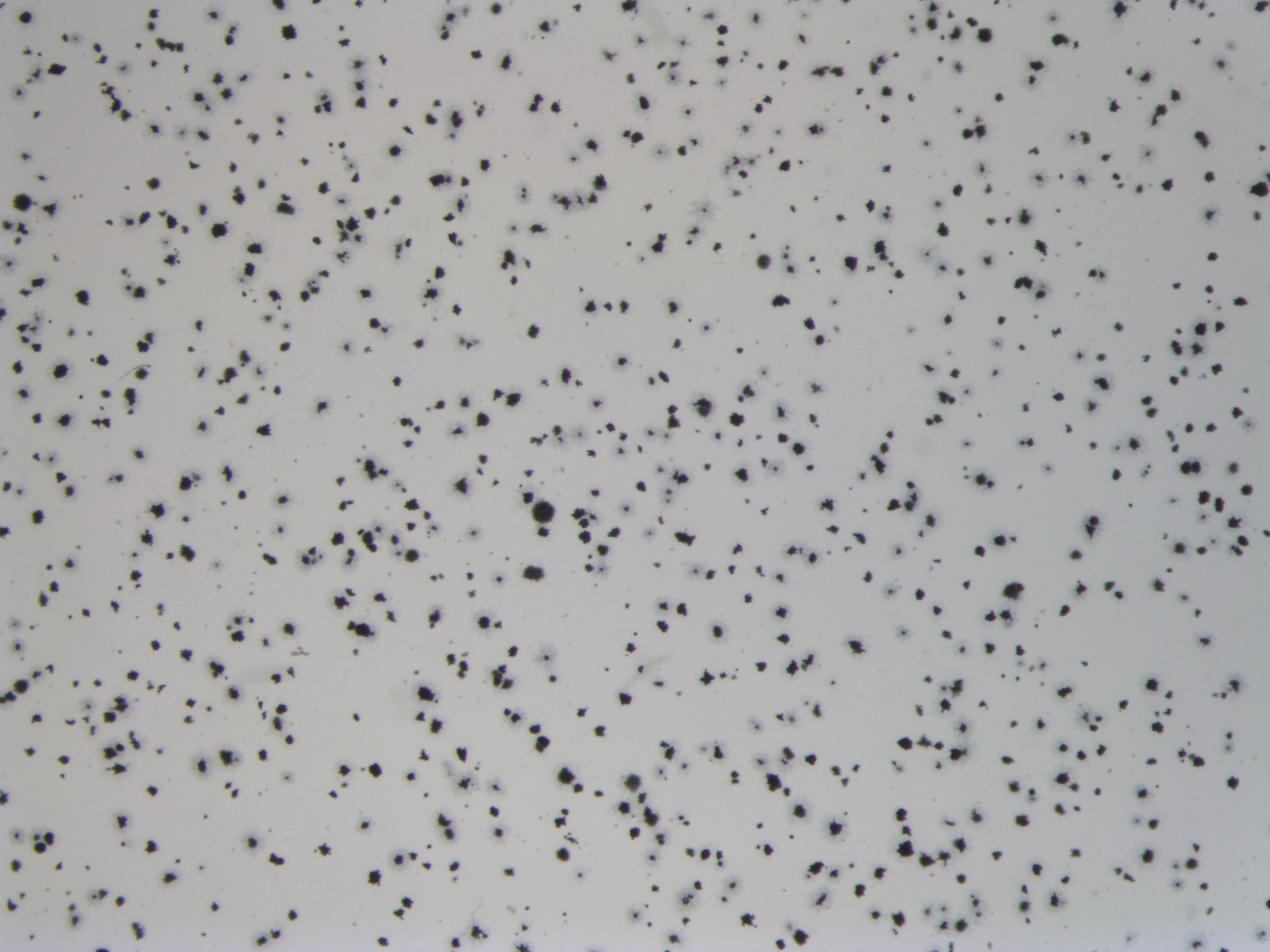

Supplement: Supplementary file 13 — Appendix Figures Source Data [file 44319_2023_17_MOESM13_ESM.zip › 44319_2023_17_MOESM13_ESM/Appendix Fig S5_source data/Appendix FigS5A_source data.tif]

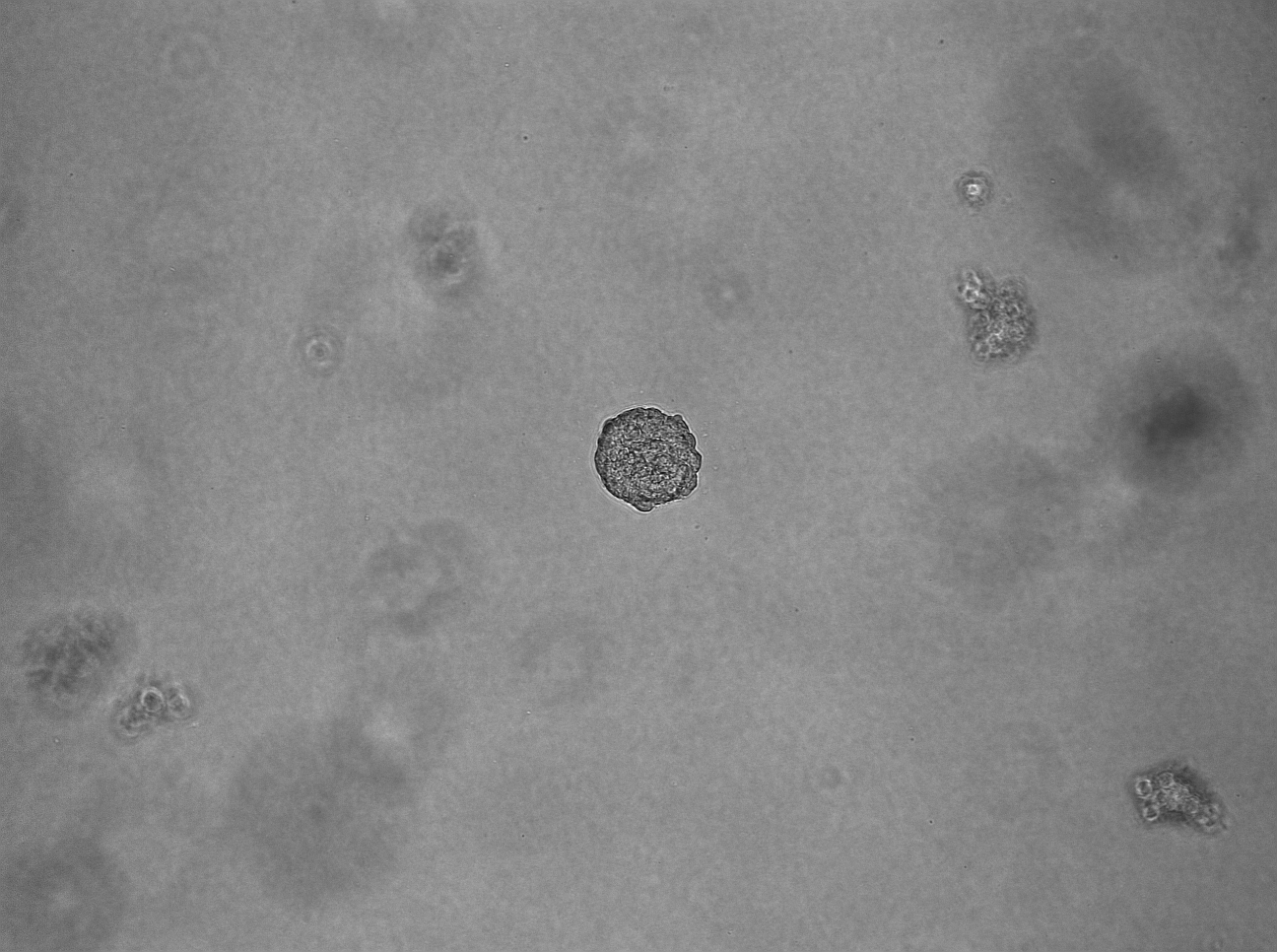

Supplement: Supplementary file 13 — Appendix Figures Source Data [file 44319_2023_17_MOESM13_ESM.zip › 44319_2023_17_MOESM13_ESM/Appendix Fig S5_source data/Appendix FigS5B_source data.tif]

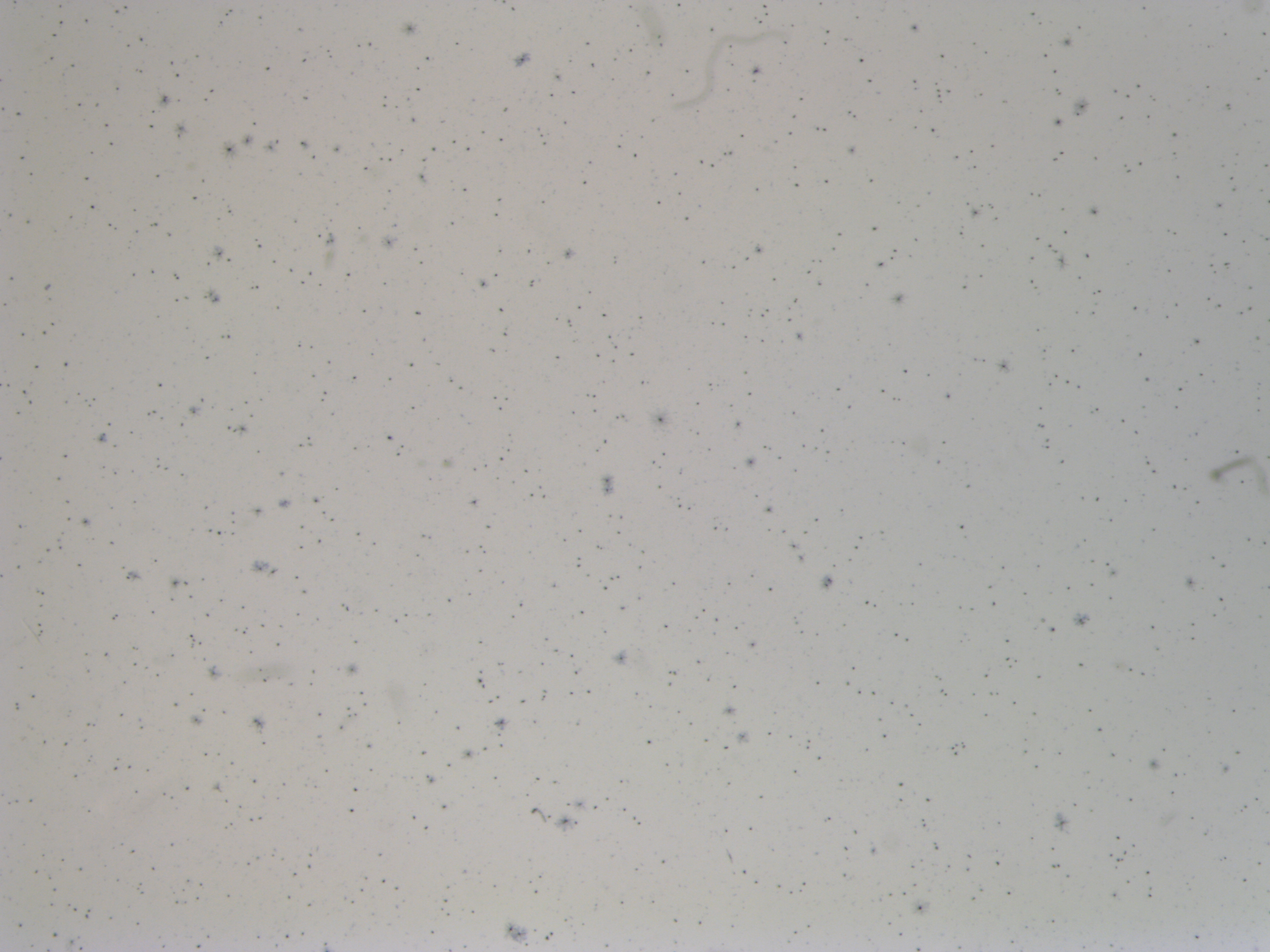

Supplement: Supplementary file 13 — Appendix Figures Source Data [file 44319_2023_17_MOESM13_ESM.zip › 44319_2023_17_MOESM13_ESM/Appendix Fig S5_source data/Appendix FigS5C_source data.tif]

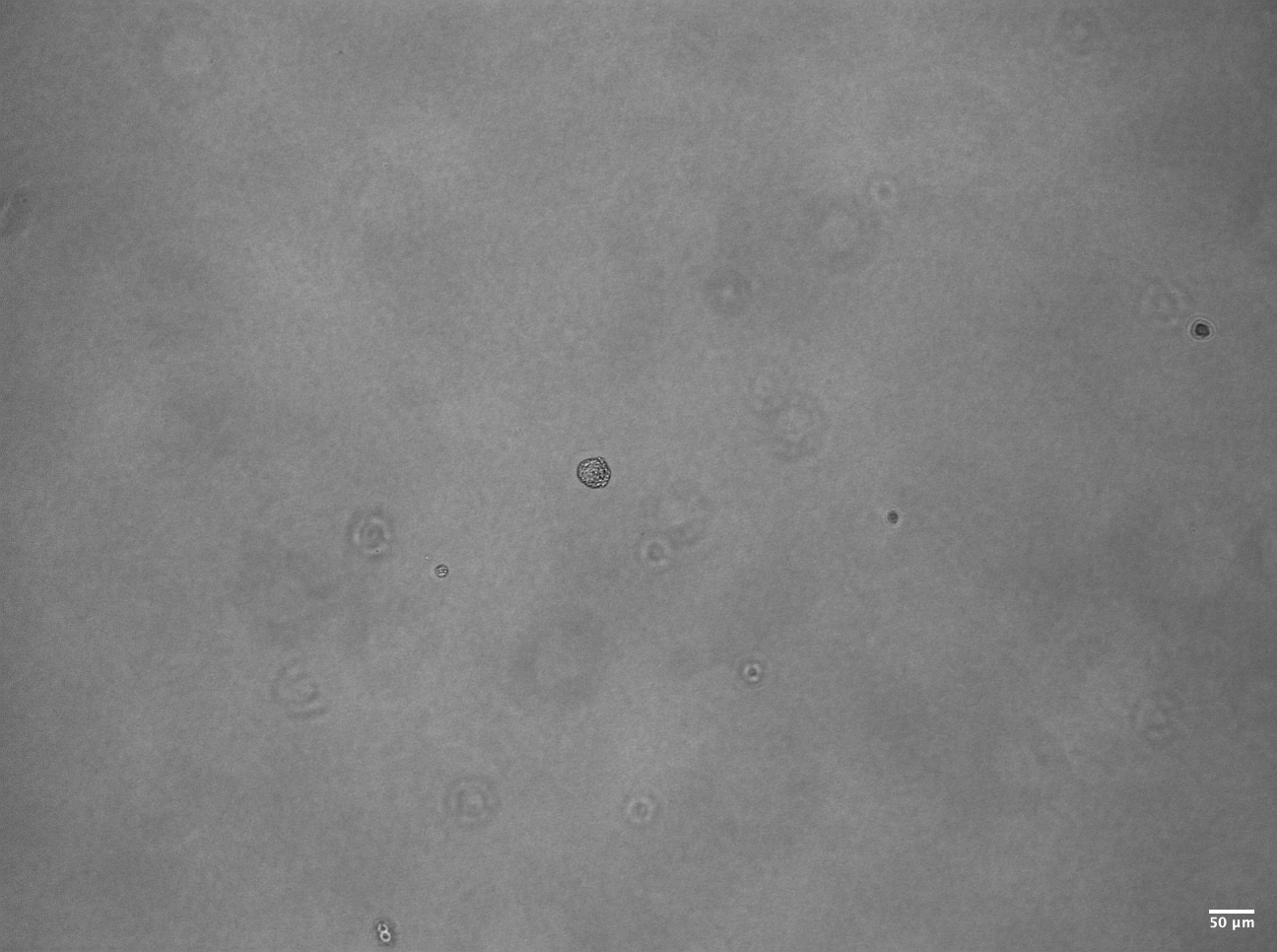

Supplement: Supplementary file 13 — Appendix Figures Source Data [file 44319_2023_17_MOESM13_ESM.zip › 44319_2023_17_MOESM13_ESM/Appendix Fig S5_source data/Appendix FigS5D_source data.tif]

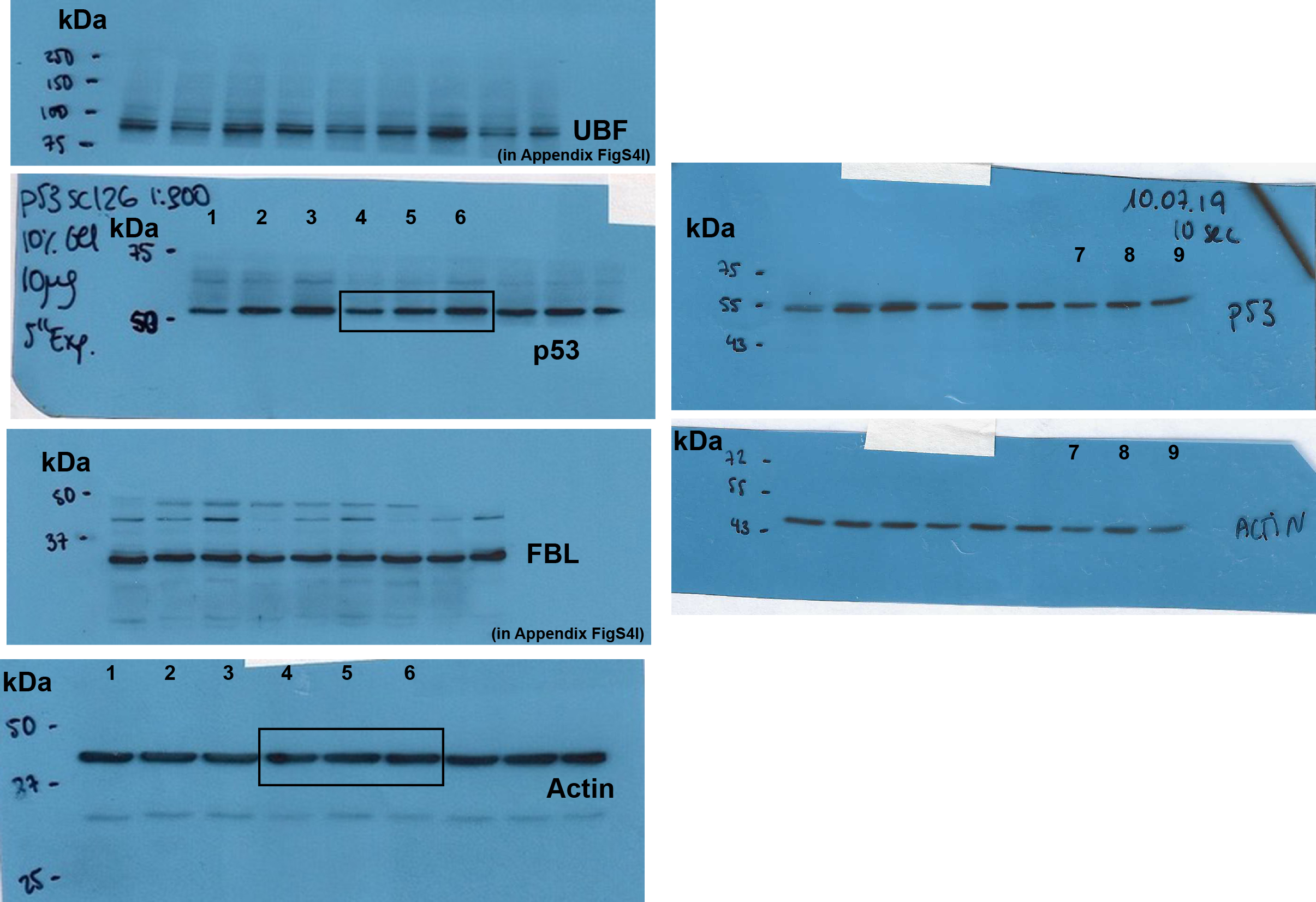

Supplement: Supplementary file 13 — Appendix Figures Source Data [file 44319_2023_17_MOESM13_ESM.zip › 44319_2023_17_MOESM13_ESM/Appendix FigS6A_source data/Appendix FigS6A_source data.jpg]
