# Supplementary material for: Ribogenesis boosts controlled by HEATR1-MYC interplay promote transition into brain tumour growth
Source: EMBO Rep. 2024 Jan 15;25(1):14. doi: 10.1038/s44319-023-00017-1 (PMC10897169; doi:10.1038/s44319-023-00017-1)
Supplement: Supplementary file 9 — Source Data Fig. 4 [file 44319_2023_17_MOESM9_ESM.zip › Fig4_source data/Fig4W_source data/Read-me file_Fig4W.docx]

Western blot lanes:

1, 4 and 7 – Control protein lysates

2, 5 and 8 – Protein lysates of cells transduced with viral particles expressing *HEATR1*-shRNA#22

3, 6 and 9 – Protein lysates of cells transduced with viral particles expressing *HEATR1*-shRNA#97
