# Supplementary material for: Ribogenesis boosts controlled by HEATR1-MYC interplay promote transition into brain tumour growth
Source: EMBO Rep. 2024 Jan 15;25(1):14. doi: 10.1038/s44319-023-00017-1 (PMC10897169; doi:10.1038/s44319-023-00017-1)
Supplement: Supplementary file 12 — Source Data Fig. 7 [file 44319_2023_17_MOESM12_ESM.zip › 44319_2023_17_MOESM12_ESM/Fig7_source data/Fig7E_source data/Read-me file_Fig7E.docx]

Western blot lanes:

1, 3 and 5 – Control protein lysates

2, 4 and 6 – Protein lysates of cells transfected with *HEATR1*-esiRNA
