# Supplementary material for: Ribogenesis boosts controlled by HEATR1-MYC interplay promote transition into brain tumour growth
Source: EMBO Rep. 2024 Jan 15;25(1):14. doi: 10.1038/s44319-023-00017-1 (PMC10897169; doi:10.1038/s44319-023-00017-1)
Supplement: Supplementary file 14 — Expanded View Figures [file 44319_2023_17_MOESM14_ESM.pdf]

Expanded View Figures

Figure EV1. Expression and ontology analysis of human orthologues of identified genes in *brat* TIC transcriptomics.

(**A**) Percentage of grade II, III and IV gliomas with *TRIM3* homozygous or hemizygous deletions. Biological replicates: 226 (grade II); 244 (grade III); 510 (grade IV). (**B**) RT-qPCR analysis of *TRIM3* in grade II Diffuse Astrocytoma (DA), GBM and non-tumoral control brain samples. Biological replicates: 5–19. Unpaired two-tailed t-tests. Box plot represent 25th and 75th percentiles, central black bands indicate medians, central red bands specify means, whiskers indicate 10th and 90th percentiles. (**C**) Immunoblots of TRIM3 in GSCs (GSC-5). Signal in mouse cerebellum shown as positive control. β-Actin: loading control. (**D**) RT-qPCRs of a subset of identified genes in grade II DA and GBM (upper panel), or in GSCs (lower panel), versus non-tumoral brain tissue (Fold Change, FC). Error bars: s.e.m. Biological replicates: 5–19 (upper panel), 3–9 (lower panel). Technical replicates: 1–3 (lower panel). Unpaired two-tailed t-tests followed by Holm correction. (**E**) GSEA of human orthologues of genes differentially expressed in *brat* TICs versus control iINPs (single best matches of dataset FDR < 0.1; DIOPT score ≥ 5) using as ranked list the gene dataset from the GSC genome-wide CRISPR screening ordered by Bayes Factor (BF) (MacLeod et al, 2019). Higher BF values indicate greater confidence in GSC fitness reduction after CRISPR-Cas9 gene knockout. NES, normalized enrichment score. Empirical p-value estimated based on 10^7 random permutations. (**F**) Overrepresented KEGG pathways in identified human orthologues dataset (DIOPT score ≥ 5). Pathways also grouped in broader categories. Data information: ***$p \leq 0.001$; **$p \leq 0.01$; *$p \leq 0.05$; $p > 0.05$, ns (non-significant).

© The Author(s)

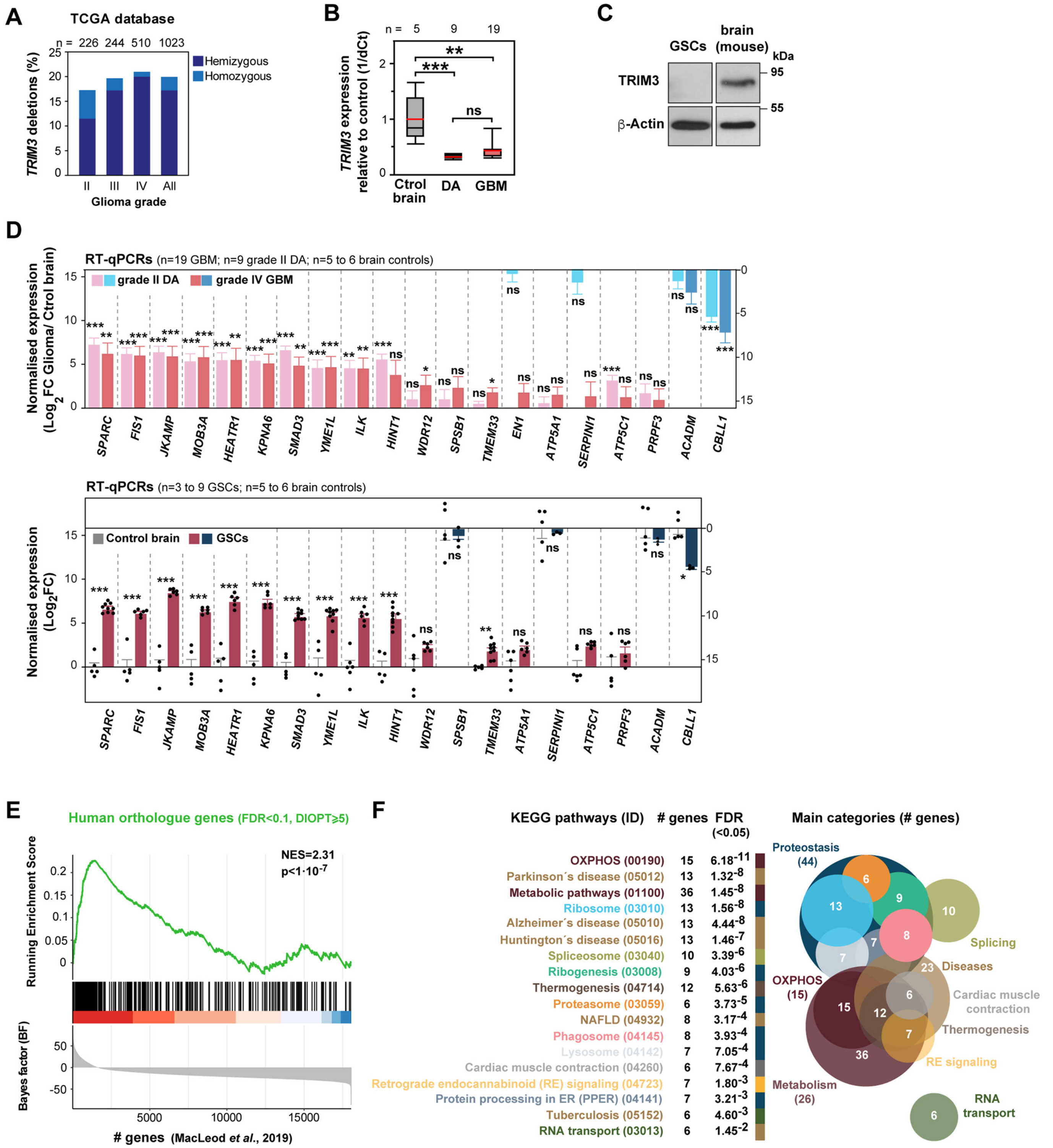
A
TCGA database
TRIM3 deletions (%)
Hemizygous
Homozygous
Glioma grade
B
TRIM3 expression relative to control (1/dCt)
Ctrol brain
DA
GBM
C
GSCs
brain (mouse)
TRIM3
β-Actin
kDa
D
RT-qPCRs (n=19 GBM; n=9 grade II DA; n=5 to 6 brain controls)
grade II DA
grade IV GBM
Normalised expression (Log2FC Glioma/ Ctrol brain)
RT-qPCRs (n=3 to 9 GSCs; n=5 to 6 brain controls)
Control brain
GSCs
Normalised expression (Log2FC)
E
Human orthologue genes (FDR<0.1, DIOPT≥5)
Running Enrichment Score
NES=2.31
p<1·10-7
Bayes factor (BF)
genes (MacLeod et al., 2019)
F
KEGG pathways (ID)
genes
FDR (<0.05)
Main categories (# genes)
OXPHOS (00190) 15 6.18-11
Parkinson´s disease (05012) 13 1.32-8
Metabolic pathways (01100) 36 1.45-8
Ribosome (03010) 13 1.56-8
Alzheimer´s disease (05010) 13 4.44-8
Huntington´s disease (05016) 13 1.46-7
Spliceosome (03040) 10 3.39-6
Ribogenesis (03008) 9 4.03-6
Thermogenesis (04714) 12 5.63-6
Proteasome (03059) 6 3.73-5
NAFLD (04932) 8 3.17-4
Phagosome (04145) 8 3.93-4
Lysosome (04142) 7 7.05-4
Cardiac muscle contraction (04260) 6 7.67-4
Retrograde endocannabinoid (RE) signaling (04723) 7 1.80-3
Protein processing in ER (PPER) (04141) 7 3.21-3
Tuberculosis (05152) 6 4.60-3
RNA transport (03013) 6 1.45-2
Proteostasis (44)
Splicing
Diseases
OXPHOS (15)
Cardiac muscle contraction
Thermogenesis
RE signaling
Metabolism (26)
RNA transport

 © The Author(s)

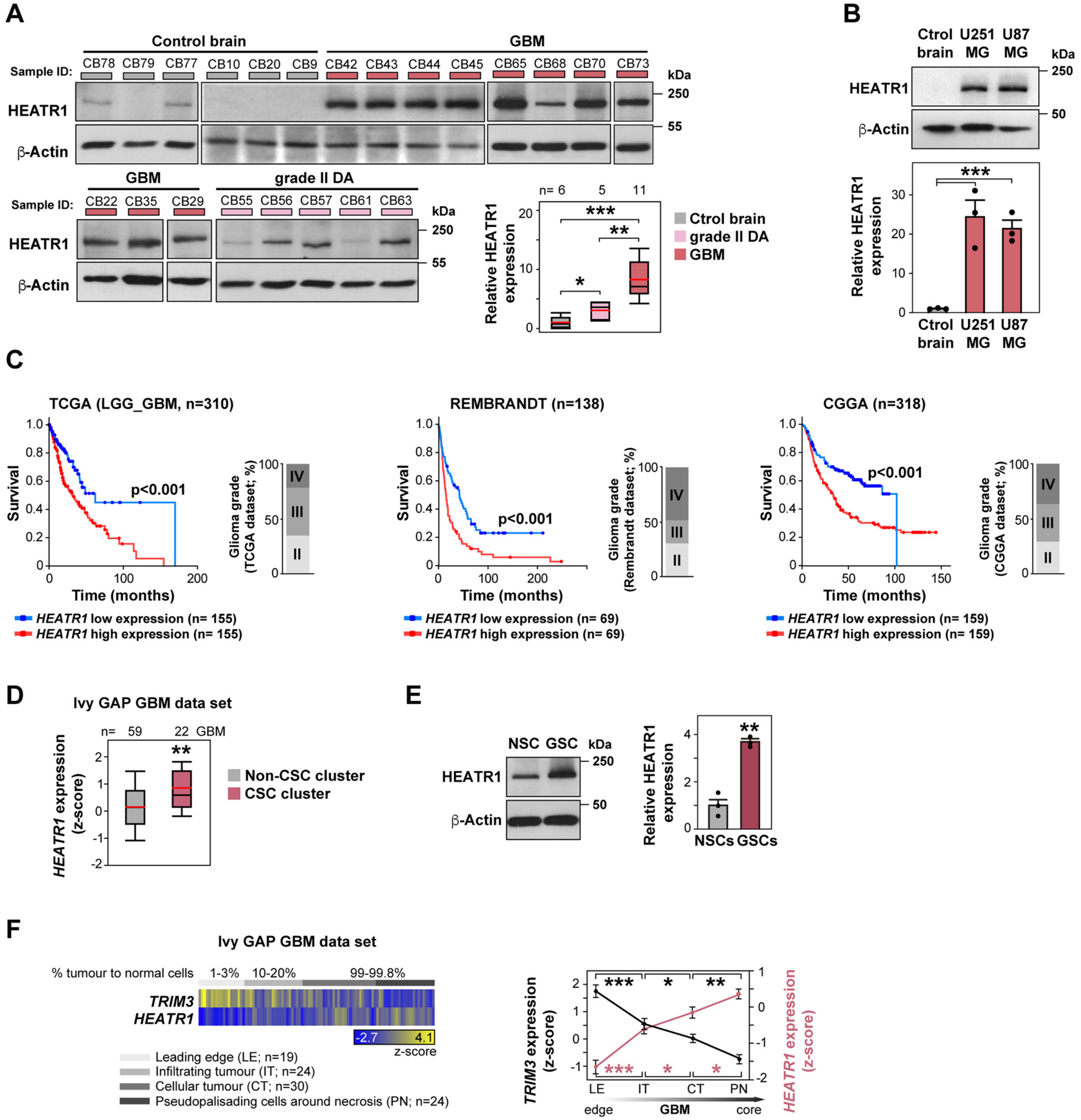

Figure EV2. *HEATR1* expression is increased in glioma and inversely correlates with patient survival.

(**A**, **B**) Immunoblots of HEATR1 in grade II DA, GBM and non-tumoral control (Ctrol) brain samples (**A**; biological replicates: 5–11), as well as in U251MG, U87MG GBM cell lines and control brain (**B**; biological replicates: 3). β-Actin: loading control. Error bars: s.e.m. Quantification of HEATR1 signal. Unpaired two-tailed t-tests. (**C**) Relative patient survival with the 25% higher (red) or 25% lower (blue) *HEATR1* expression in glioma (grades II to IV). Biological replicates: 310 (TCGA); 138 (REMBRANDT); 318 (CGGA). Log-Rank *p*-values. (**D**) *HEATR1* expression in putative cancer stem cell (CSC) and non-CSC clusters in GBM. Clusters identified by expression of 17 reference probes via in situ hybridization, Ivy GAP. Biological replicates: 22–59. Unpaired two-tailed t-test. (**E**) HEATR1 expression analysis by immunoblotting in GSCs (GSC-5) versus NSCs (biological replicates: 3). β-Actin: loading control; Error bars: s.e.m.; Unpaired two-tailed t-tests. (**F**) *TRIM3* and *HEATR1* relative expression levels in four GBM regions indicated. Tumour to normal cell ratios (%). Sample numbers for each region: Leading edge, 19; Infiltrating tumour, 24; Cellular tumour, 30; Pseudopalisading cells around necrosis, 24. RNAseq data, IVY GAP. Unpaired two-tailed t-tests. Error bars: s.e.m. Data information: Box plots represent 25th and 75th percentiles, central black bands indicate medians, central red bands specify means, whiskers indicate 10th and 90th percentiles. ***$p \leq 0.001$; **$p \leq 0.01$; *$p \leq 0.05$; $p > 0.05$.

© The Author(s)

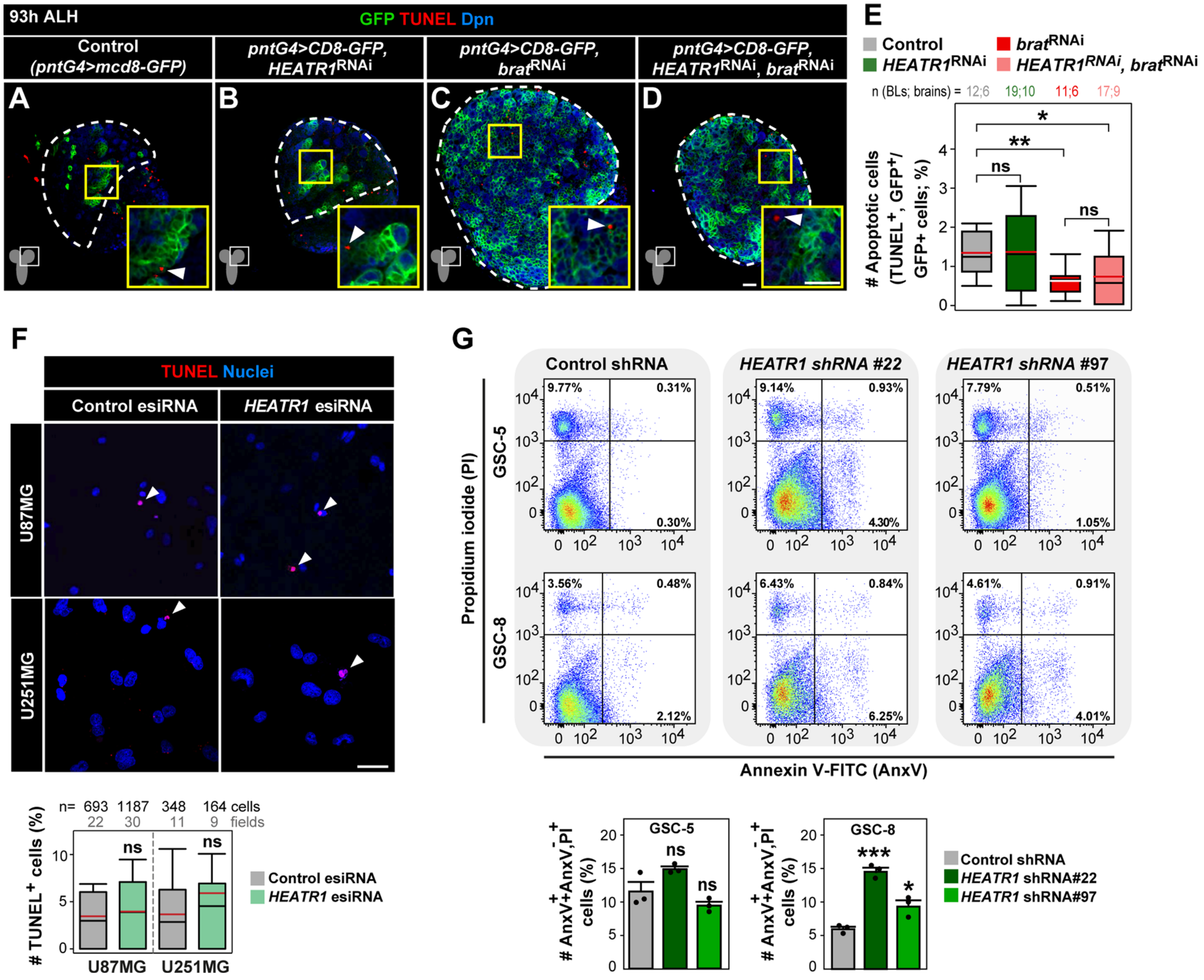

Figure EV3. ***HEATR1*** **inhibition has no impact in** ***brat*****-deficient tumour, control brain or GBM cell death but exerts a mild effect on GSCs.**

(**A–E**) TUNEL labelling and immunostainings of GFP and Dpn in type II NSC lineages expressing *CD8-GFP* (no-tumour control) and *HEATR1*^{RNAi}, *brat*^{RNAi} (tumour) or *HEATR1*^{RNAi}, *brat*^{RNAi} (*HEATR1*-deficient tumour) at 93 h ALH. TUNEL^{+}GFP^{+} quantification (% of GFP^{+} cells, biological replicates: 11–19, unpaired two-tailed t-tests; **E**). Dashed lines: central brain region; Insets: higher magnifications. White arrowheads: TUNEL^{+} cells. Scale bars: 10 μm. (**F**) TUNEL labelling of U87MG and U251MG cells 48 hpt with *HEATR1*-esiRNA or control *GFP*-esiRNA. Nuclei (DAPI). Scale bar: 50 μm. Arrowheads: TUNEL^{+} cells. TUNEL^{+} cell quantification: % of DAPI^{+} cells; 22–30 (U87MG) and 9–11 cell images (fields) from 3 biological replicates; unpaired two-tailed t-tests. (**G**) Annexin-V-FITC (AnxV) and red fluorescent Propidium Iodide (PI) labelling in GSCs (GSC-5; GSC-8) by flow cytometry. Representative dot plots (% cells in gated subpopulations). Quantification of cell subpopulations: 12,000–50,000 cells per condition from 3 biological replicates, error bars: s.e.m., unpaired two-tailed t-tests. Data information: Box plots represent 25th and 75th percentiles, central black bands indicate medians, central red or white bands specify means, whiskers indicate 10th and 90th percentiles. ***$p \leq 0.001$; **$p \leq 0.01$; *$p \leq 0.05$; $p > 0.05$, ns (non-significant).

 © The Author(s)

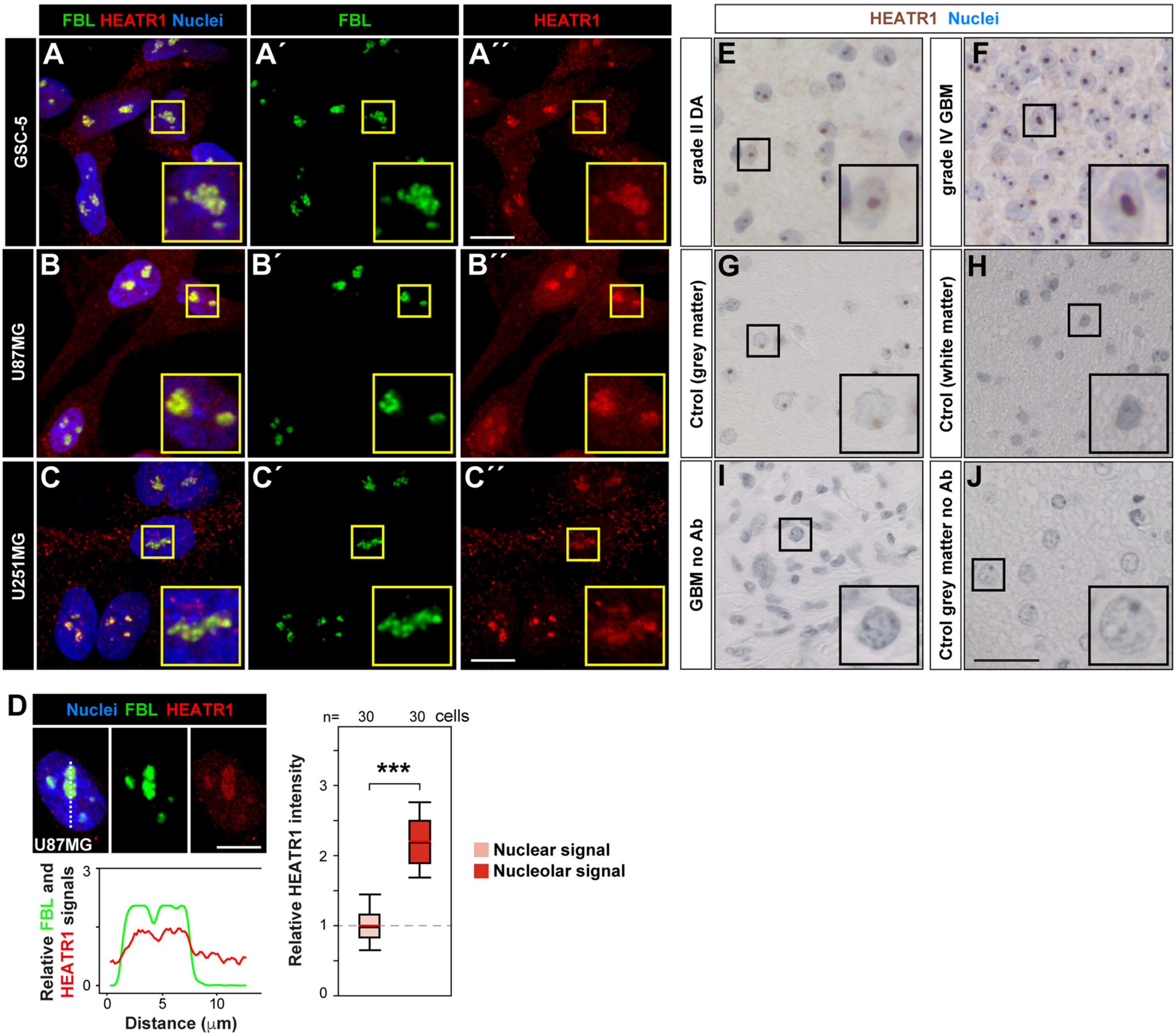

Figure EV4. HEATR1 localises predominantly in nucleoli of glioma cells.

(**A–C″**) Immunostainings of HEATR1 and FBL in GSCs (GSC-5; **A–A″**) and GBM cell lines (U87MG, **B–B″**; U251MG, **C–C″**). Nuclei (DAPI). FBL (**A′–C′**) and HEATR1 (**A″–C″**) single channels in green and red, respectively. Insets: higher magnification. (**D**) Representative image and intensity profile plot for FBL and HEATR1 relative signals in nucleus and nucleoli regions (dashed line; left panels) of U87MG cells. Relative nucleolar to nuclei HEATR1 signal quantification, 30 cells scored from 3 biological replicates, unpaired two-tailed t-test (right panel). Box plot represent 25th and 75th percentiles, central black bands indicate medians, central red bands specify means, whiskers indicate 10th and 90th percentiles. ***$p \leq 0.001$. (**E–J**) Immunohistochemistry of HEATR1 in grade II DA ($n = 9$; **E**), GBM ($n = 9$; **F**) and control non-tumour brain samples in grey (**G**) and white (**H**) matter regions ($n = 10$). No primary antibody controls (**I**, **J**). Nuclei: haematoxylin counterstain. See Fig. EV2 for HEATR1 protein quantifications. Data information: Scale bars: 10 μm.

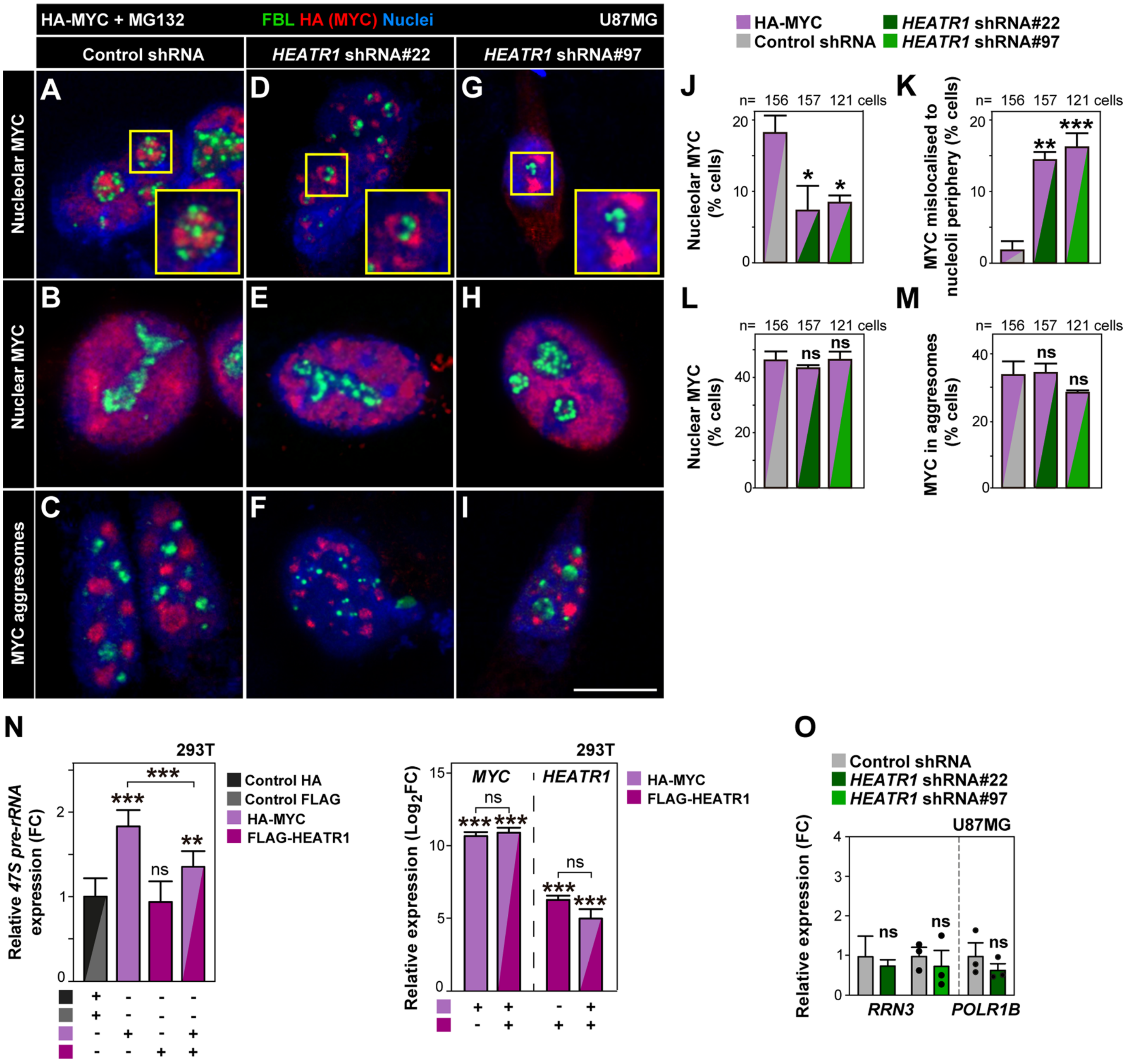

Figure EV5. HEATR1 is required for exogenous MYC localisation in nucleoli.

(**A–M**) Immunostainings of FBL and HA-MYC in U87MG GBM cells 168 hpi with *HEATR1*-shRNAs (**D–I**) or control shRNAs (**A–C**) and 6 h after transfection with HA-tagged full-length MYC (HA-MYC) plus MG132 treatment. Nuclei (DAPI). Insets: higher magnification. Scale bar: 10 μm. Quantification of cells (% of DAPI$^+$) showing nucleolar MYC (**J**), mislocalised MYC into nucleolar periphery (**K**), nuclear MYC (**L**) and MYC in nuclear aggregates (aggresomes; M). Error bars: s.e.m. 121–157 cells scored from 3–4 biological replicates. Unpaired two-tailed t-tests. (**N**) RT-qPCR analysis of *47S* pre-RNA (left panel) in 293T cells transfected with HA-tagged MYC (HA-MYC) or control HA vector, and Flag-tagged HEATR1 (Flag-HEATR1) or control Flag vector. RT-qPCR analysis of *MYC* and *HEATR1* (right panel) in 293T cells expressing Flag-HEATR1 and/or HA-MYC versus controls. Fold change (FC). Biological replicates: 3, technical replicates: 3. Error bars: s.e.m. Unpaired two-tailed t-tests except Mann–Whitney test in *HEATR1* analysis on double HEATR1-FLAG, HA-MYC expressing samples. (**O**) RT-qPCR analysis of *RRN3* and *POLR1B* (fold change, FC) in U87MG cells 168 hpi with *HEATR1*-shRNAs versus control shRNAs. Fold change (FC). Biological replicates: 3; technical replicates: 2 (*RRN3* upon *HEATR1* shRNA# 22 versus control). Error bars: s.e.m. Unpaired two-tailed t-tests except *RRN3* analysis upon *HEATR1* shRNA# 22 versus control (Mann–Whitney test). Data information: ***$p \leq 0.001$; **$p \leq 0.01$; *$p \leq 0.05$; $p > 0.05$, ns (non-significant).

 © The Author(s)
